# Lithium achieves sequence selective ring-opening terpolymerisation (ROTERP) of ternary monomer mixtures†

**DOI:** 10.1039/d2sc01776h

**Published:** 2022-05-05

**Authors:** Susanne Rupf, Patrick Pröhm, Alex J. Plajer

**Affiliations:** Intitut für Chemie und Biochemie, Freie Universität Berlin Fabeckstraße 34-36 14195 Berlin Germany plajer@zedat.fu-berlin.de

## Abstract

Heteroatom-containing degradable polymers have strong potential as sustainable replacements for petrochemically derived materials. However, to accelerate and broaden their uptake greater structural diversity and new synthetic methodologies are required. Here we report a sequence selective ring-opening terpolymerisation (ROTERP), in which three monomers (A, B, C) are selectively enchained into an (ABA′C)_*n*_ sequence by a simple lithium catalyst. Degradable poly(ester-*alt*-ester-*alt*-trithiocarbonate)s are obtained in a *M*_n_ range from 2.35 to 111.20 kDa which are not easily accessible *via* other polymerisation methodologies. The choice of alkali metal is key to achieve high activity and to control the terpolymer sequence. ROTERP is mechanistically compatible with ring-opening polymerisation (ROP) allowing switchable catalysis for blockpolymer synthesis. The ROTERP demonstrated in this study could be the first example of an entirely new family of sequence selective terpolymerisations.

## Abstract 2

A sequence selective ring-opening terpolymerisation of epoxides with CS_2_ and phthalic thioanhydride yielding poly(ester-*alt*-ester-*alt*-trithiocarbonates) is reported.

## Graphical abstract

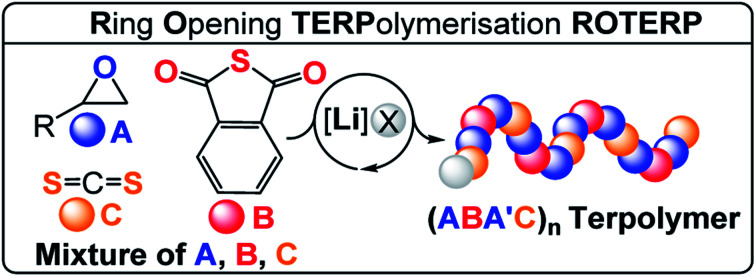

Synthetic polymers are now more in demand than ever before and looking at their annually increasing production, a polymer free society is at best a vague memory rather than a vision for the future.^1^ As most commodity polymers are based on chemically inert aliphatic –C–C– backbones, most polymer waste shows unappreciable degradation with respect to the polymer's time in application.^2,3^ In answer to the ever-increasing amount of plastic pollution, much effort focuses on the exploration of new heteroatom containing polymers which, because of their more polar bonds making up the backbone, are more susceptible to degradation *via* physical, chemical and biochemical pathways and even facilitate new chemical recycling methods.^4–8^ Besides, there is also a constant demand for entirely new materials to enable technological innovation making new methodologies to synthesise heteroatom containing polymers necessary.

Arguably one of the most popular methods to make such polymers is the ring-opening polymerisation (ROP) of a heterocycle A.^9–11^ These polymerise under release of their associated ring strain energy to make polymers (A)_*n*_ such as poly(thio)ethers, poly(thio)ester and poly(thio)carbonates, only to name a few. Early on it has been realised that in some cases the ROP of three or four-membered heterocycles A can be coupled to the insertion of typically heteroallenes or cyclic anhydrides B to generate alternating copolymers (AB)_*n*_.^12–14^ The underlying requirements for (AB)_*n*_ polymerisations are a combination of kinetic factors, *i.e.* monomer A inserting orders of magnitude faster into the active catalyst growing chain-bond than monomer B, and chemoselectivities, *i.e.* insertion of monomer A resulting in type A active catalyst growing chain-bond that does not show appreciable reactivity with A but only with B (and *vice versa*). Prominent examples of this alternating (AB)_*n*_ ring-opening copolymerisation (ROCOP) include CO_2_/epoxide ROCOP yielding polycarbonates and cyclic anhydride/epoxide ROCOP yielding polyesters.^15–17^ Sulfur analogous are also accessible such as polythiocarbonate from CS_2_/(epoxide or thiirane) and polythioesters from thioanhydride/(epoxide or thiirane) ROCOP.^18–31^ Such sulfur rich polymers are attractive high-refractive index materials that can show improved crystallinity and degradability over their all-oxygen analogues and in some cases enable chemical polymer to monomer recycling.^32–39^

Most relevant to this study is a report by *Werner* and coworkers on lithium alkoxide catalysed CS_2_/epoxide ROCOP yielding poly(monothio-*alt*-trithiocarbonate)s featuring R–O–C(=S)–O–R and R–S–C(=S)–S–R links (Fig. 1).^40,41^ Such a polymer sequence is unexpected, as the formal product of alternating insertion would be a poly(dithiocarbonate) with R–O–C(=S)–S–R links. Furthermore, the polymer shows an unusual “head-to-head-*alt*-tail-to-tail” selectivity meaning that the R–O–C(=S)–O–R links sit adjacent to the tertiary CHR_3_ positions of the ring opened epoxide (*i.e.* “head” position) and that the R–S–C(=S)–S–R links sit adjacent to the secondary CH_2_R_2_ position of the ring opened epoxide (*i.e.* “tail” position). This sequence let the authors postulate a mechanism involving tail-selective epoxide ring-opening by a dithiocarbonate chain end which is formed by CS_2_ insertion into an alkoxide intermediate. The resulting alkoxide intermediate was proposed to backbite into the adjacent dithiocarbonate link which after a rearrangement process resulted in an O/S exchange of the chain end. The rearrangement transforms the alkoxide into a thiolate chain end and the adjacent dithiocarbonate R–O–C(=S)–S–R into a monothiocarbonate R–O–C(=S)–O–R. CS_2_ insertion of the thiolate generates a trithiocarbonate R–S–C(=S)–S–R which after epoxide insertion regenerates the alkoxides. In contrast to alkoxides sitting adjacent to R–O–C(=S)–S–R links, alkoxides next to R–S–C(=S)–S–R links were not proposed to undergo backbiting and O/S exchange. Importantly the initial regioselectivity of the epoxide ring-opening was preserved throughout the rearrangement which explained the “head-to-head-*alt*-tail-to-tail” selectivity. Interestingly lithium appeared to be crucial as other alkali metal alkoxides failed to catalyse this ROCOP, while more sophisticated catalysts result in much more uncontrolled polymer sequences. Hence it appears that the Li controls which alkoxide intermediate precisely undergoes O/S exchange and to which degree this rearrangement occurs, but the reasons behind the special role of Li remains to be explored. Relatedly the ROCOP of thioanhydrides with epoxides has also been reported and similar exchange processes have been proposed as side reactions.^23^ Although not directly proven, this mechanism seemed reasonable and let us hypothesise that lithium catalysts could grant a general access to control the O/S exchange process in ROCOP and even in the polymerisation of ternary monomer mixtures. Furthermore, we reasoned that the two distinct chain ends formed *via* O/S exchange, *i.e.* alkoxide and thiolate (type A *vide supra*), could enable discrimination between two different type B monomers and enable sequence selective terpolymerisations. It should be noted that reports exist in which mixtures of A, B and C (*e.g.* epoxide A, cyclic anhydride B and CO_2_ C) either yield random terpolymers, (AB)_*n*_-*ran*-(AC)_*m*_ poly(esters-*ran*-carbonate), or block-terpolymers, (AB)_*n*_-*b*-(AC)_*m*_, polyester-*b*-polycarbonate. In this case the monomer sequence depends on catalyst selection and reaction conditions, but sequence selective terpolymers, *e.g.* (ABC)_*n*_ or (ABAC)_*n*_, are unknown.^42–48^ The hypothesis of O/S exchange here led us to discover a new type of polymerisation, sequence selective ring-opening terpolymerisation (ROTERP) that we report in this contribution. ROTERP produces poly(ester-*alt*-ester-*alt*-trithiocarbonates), *i.e.* (ABA′C)_*n*_ sequences, from a mixture of the monosubstituted epoxides propylene oxide (PO) or butylene oxide (BO) A, phthalic thioanhydride (PTA) B and CS_2_ C, employing simple lithium salts as the catalyst. Furthermore, model reactions proof the previously postulated O/S rearrangement that enable ROTERP and elucidate the role of the lithium catalyst. Finally, we employed ROTERP in so-called switchable catalysis, in which the onset of ROTERP stops the occurrence of ROP, for the construction of blockpolymers.

**Fig. 1:**
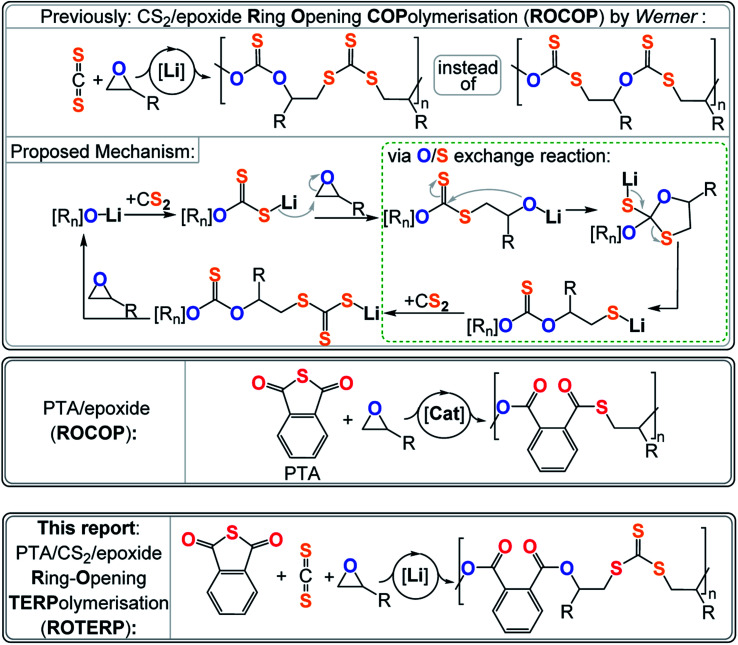
(Top) CS_2_/epoxide ROCOP and postulated mechanism involving a central O/S exchange reaction, (middle) phthalic thioanhydride (PTA)/epoxide ROCOP and (bottom) PTA/CS_2_/epoxide ROTERP reported in this study. R = Me, Et; [R_*n*_] = polymer chain.

ROTERP of mixtures of PTA, PO and CS_2_ at loadings typically employed in ROCOP catalysis with lithium hexamethyldisilazide (LiHDMS) or lithium benzyloxide (LiOBn) as the catalyst at loadings in the range of 1 eq. LiX : (6.25–500 eq.) PTA : (31.25–2500 eq.) PO : (62.5–5000 eq.) CS_2_ yield polymeric materials in 95% selectivity at 80 °C (see Table 1) alongside small amounts (5%) of cyclic dithiocarbonate (c5c).^40,47^ Spectroscopic analysis of the isolated polymer reveals surprisingly clean NMR spectra given the potential for statistical terpolymerisation of these three monomers. The ^1^H NMR spectrum (Fig. 2) shows two main aryl resonances for a symmetrically substituted terephthalate unit from ring opened PTA (*δ* = 7.72 and 7.56 ppm) as well as one main resonance for the CHMe (*δ* = 5.42 ppm; head position of the ring-opened epoxide) and CH_2_ (*δ* = 3.90–3.40 ppm; tail position of the ring opened epoxide) groups respectively stemming from the ring opened PO. Correspondingly the ^13^C{^1^H} NMR spectrum (ESI Fig. S1†) reveals the almost exclusive presence of trithiocarbonate R–S–C(=S)–S–R (*δ* = 222.8 ppm) and arylester R–C(=O)–O–R (*δ* ∼166.6 ppm) resonances (94–98%) alongside minor thioester R–C(=O)–S–R (*δ* = 192.7 ppm) resonances (2–6%). ^1^H and 2D NMR spectra (ESI Fig. S3†) show that trithiocarbonate units are positioned adjacent to CH_2_ groups while arylesters are connected to the tertiary CHMe groups. The spectra remain unchanged after multiple precipitations from DCM : MeOH or THF : pentane and DOSY NMR shows that all ^1^H NMR resonances diffuse at the same rate confirming that these are part of the same species. Furthermore, no other type of thiocarbonate R–(O/S)–C(=O/S)–(O/S)–R are part of the polymer. Linkage identity was further substantiated by the ATR-IR spectrum (ESI Fig. S5†) of these polymers showing an arylester C=O stretch at *ν̃* = 1716 cm^−1^ as well as a thiocarbonate C=S stretch at *ν̃* = 1062 cm^−1^ (ESI Fig. S5†). Accordingly, the polymers are obtained as yellow solids due the presence of the C=S chromophore (*λ*_abs_ = 435 nm, ESI Fig. S6†). MALDI-TOF analysis unfortunately only led to decomposition of the materials and no signals could be identified as previously reported for sulfur-rich polymers.^26,38^ Nevertheless ^1^H NMR allows some conclusions regarding the topology as both for LiHMDS or LiOBn initiation, resonances for the HMDS and OBn groups can be identified to be part of the purified polymers. Insertion of alkalimetal alkoxides and amides into CS_2_ yielding alkali dithiocarbonates and dithiocarbamates have been previously reported.^40,79^ This makes initiation *via* CS_2_ insertion likely which defines one end of the polymer and hence indicates that chains are linear rather than cyclic. As ROTERP is followed by CS_2_/epoxide coupling once all PTA is consumed (*vide infra*) we infer that chains are terminated by CS_2_ because heteroallene insertion products are generally established to be the resting states of heteroallene/heterocycle coupling reactions.^12^

**Table 1:** Data showing PTA/CS_2_/epoxide ROTERP under different conditions

Run	LiX^f^ : PTA : (PO/*BO) : CS_2_^a^	Time [min]	PTA conv.	Polym. select.^b^	Linkage select.^c^	*M*_n_ [kDa] (*Đ*)^d^	*M*_n,theo_ [kDa]
#1	1 : 6.25 : 31.25 : 62.5	<1 min	>99%	95%	98%	2.35 (1.44)	2.41
#2	1 : 12.5 : 62.5 : 125	<1 min	>99%	95%	98%	5.11 (1.41)	4.81
#3	1 : 25 : 125 : 250	1 min	>99%	95%	98%	8.90 (1.53)	9.46
#4	1 : 100 : 500 : 1000	15 min	>99%	95%	95%	24.46 (1.47)	37.34
#5^e^	1 : 300 : 1500 : 3000	60 min	98%	95%	95%	55.05 (1.60)	111.70
#6^e^	1 : 500 : 2500 : 5000	16 h	93%	95%	94%	111.20 (1.76)	186.06
#7	1 : 100 : 500* : 250	15 min	>99%	95%	91%	23.45 (1.54)	38.67
#8	1 : 100 : 500* : 500	30 min	>99%	95%	92%	24.86 (1.67)	38.67
#9	1 : 100 : 500* : 1000	120 min	>99%	95%	96%	22.90 (1.55)	38.67
#10	1 : 100 : 500* : 1500	120 min	>99%	95%	97%	24.16 (1.56)	38.67
#11	1 : 300 : 500 : 1000	120 min	90%	95%	91%	55.05(1.55)	104.20
#12	1^g^ : 100 : 500* : 1000	120 min	22%	95%	77%	4.20 (1.24)	8.51
#13	1^h^ : 100 : 500* : 1000	120 min	—	—	—	—	—
#14	1 : 0 : 500* : 1000	18 h	—	0%	—	—	—
#15	1 : 100 : 500* : 0	36 h	76%	99%	40%	7.17 (1.47)	18.03
#16^i^	1 : 100 : 500* : 1000	30 min	>99%	95%	96%	22.69 (1.55)	38.67
#17^j^	1 : 100 : 500* : 1000	10 min	>99%	95%	96%	23.73 (1.60)	38.67

a Copolymerisation at *T* = 80 °C.

b Polymer selectivity, determined by comparison of the relative integrals, in the normalised ^1^H NMR spectrum (CDCl_3_, 25 °C, 400 MHz), of tertiary CH resonances due to polymer and cyclic dithiocarbonate c5c at 20–80% PTA consumption.

c Linkage selectivity, determined by comparison of the relative integrals, in the normalised the ^1^H NMR spectrum (CDCl_3_, 25 °C, 400 MHz) of resonances due to ester and trithiocarbonate linkages relative to ester, trithiocarbonate and thioester links (for #9 proportion of terephthalate and dithioterephthalate links).

d Determined by SEC (size exclusion chromatography) measurements conducted in THF, using narrow MW polystyrene standards to calibrate the instrument.

e Longer reaction time was chosen due to high viscosity of the reaction mixture.

f X = HMDS or OBn from *in situ* reaction of LiHMDS with 1 eq. BnOH.

g NaHMDS with 1 eq. BnOH was employed as the catalyst.

h KHMDS with 1 eq. BnOH was employed as the catalyst.

i *T* = 100 °C.

j *T* = 120 °C.

**Fig. 2:**
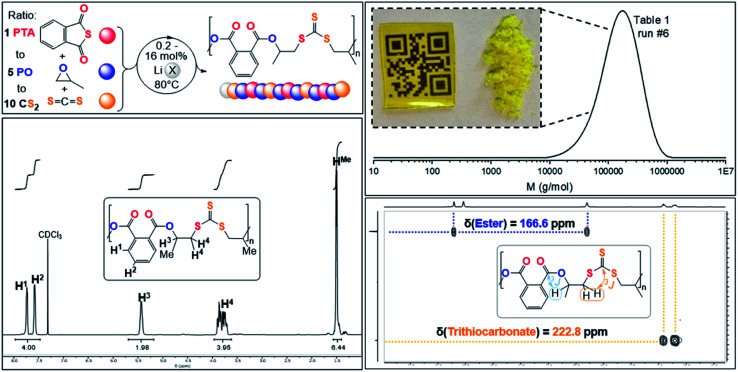
(Top left) PTA/CS_2_/PO ROTERP reaction scheme, X = HMDS, OBn; (top right) SEC trace corresponding to Table 1, run #6 and picture of polymer precipitated from the reaction mixture with MeOH as well as solvent casted film from DCM; (bottom right) ^1^H–^13^C HMBC NMR spectrum and (bottom left) ^1^H NMR (CDCl_3_, 25 °C, 500 MHz) spectrum of terpolymer corresponding to Table 1 run #3.

Analogous results are obtained with butylene oxide (BO) employed instead of PO. SEC analysis of the obtained products at various catalyst loadings shows that our new methodology can yield polymers with controllable molecular masses (*M*_n_ = 2.35–111.20 kDa; *Đ* = 1.41–1.76, ESI Fig. S25 and S26†) rendering it a useful methodology for future material synthesis.^49^ Aliquots removed at regular time intervals show a linear increase of molecular masses with PTA conversion with slightly increasing dispersity (ESI Fig. S27†) which points towards some transesterification processes occurring alongside propagation, and this is further indicated by the presence minor CH_2_-ester resonances.^50–52^ Aliquot analysis by ^1^H NMR (ESI Fig. S23†) shows uniformly growing polymer resonances forming in the reaction mixtures equivalent to those observed for the isolated polymer after full PTA consumption. This indicates poly(ester-*alt*-ester-*alt*-trithiocarbonate) formation throughout the reaction and no change of the respective link resonance ratios as ROTERP progresses pointing towards selective monomer enchainment rather than linkage formation through transesterification like processes. Taken together the results indicate a poly(ester-*alt*-ester-*alt*-trithiocarbonate) sequence as conveyed in Fig. 3 featuring a head-to-head connected terephthalate links and tail-to-tail connected trithiocarbonate links in alternation which is reminiscent of the results described by Werner and coworkers (*vide supra*).

**Fig. 3:**
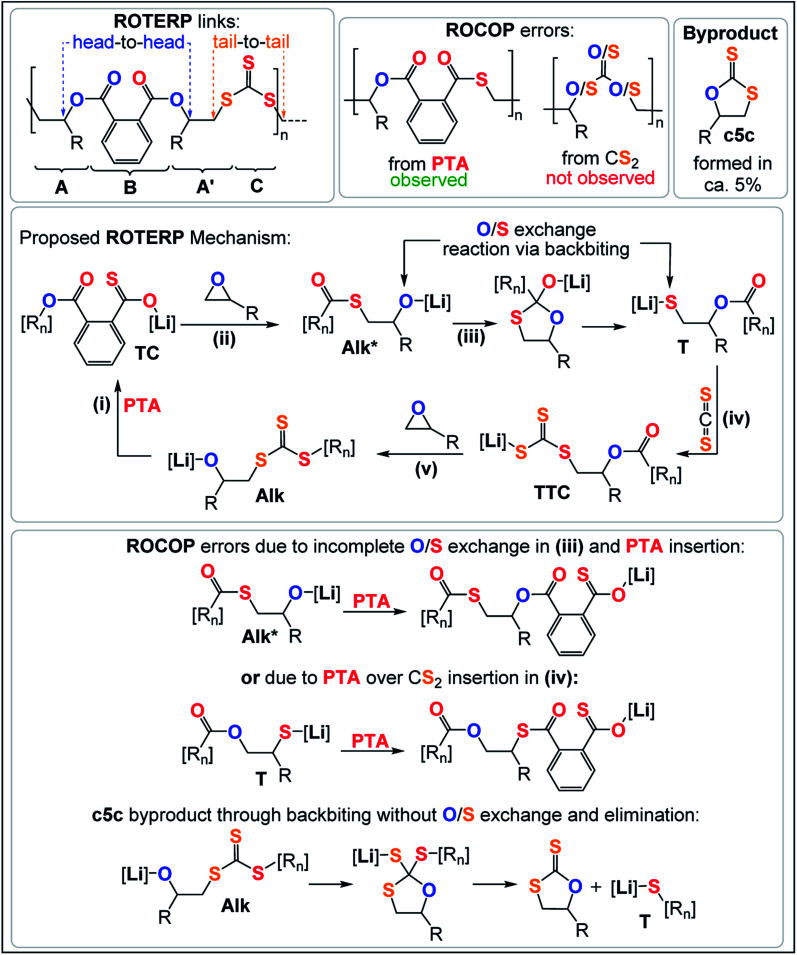
Reaction products and postulated ROTERP reaction mechanism, [R_*n*_] = polymer chain.

The related regiochemistry in addition to the fact that a similar lithium catalyst generates alternating oxygen and sulfur enriched links let us hypothesize that the ROTERP process possesses mechanistic similarities to the ROCOP process reported by Werner and this led us to propose the propagation mechanism shown in Fig. 3. Here a thiocarboxylate intermediate TC, generated from alkoxide Alk insertion into PTA in step (i), inserts into the epoxide to form a thioester appended alkoxide Alk* in step (ii). This alkoxide then rearranges in an O/S exchange process into an ester appended thiolate T in step (iii). CS_2_ insertion by T forms a trithiocarbonate intermediate TTC, which inserts into BO in step (v) to regenerate Alk. This propagation results in a (ABA′C)_*n*_ sequence with one link that derives from a ring opened epoxide A and another link from a ring opened epoxide following isomerisation (akin to a virtual thiirane) which we decided to term A′. In line with our mechanistic hypothesis, we believe that the erroneous thioester linkages are formed through incomplete O/S exchange and PTA insertion from Alk* or due to PTA insertion from the thiolate intermediate T as also shown in Fig. 3. The cyclic byproduct c5c is proposed to be formed *via* backbiting from Alk into the adjacent trithiocarbonate link where c5c elimination occurs over O/S exchange.

To explore how thioester errors are formed, we conducted a series of terpolymerisation experiments in which we changed the PTA : CS_2_ loading from 100 : 1500 eq, to 100 : 250 eq. (*versus* 1 LiHMDS eq. and 500 BO eq.) which results in an effective concentration increase of PTA while decreasing the CS_2_ concentration. This results in a gradual increase of thioester links from 3% to 9% (Table 1, runs #7–#11, ESI Fig. S14†). Increasing the PTA *vs.* CS_2_ loading from 100 : 1000 eq. to 300 : 1000 eq. likewise results in an increase in thioester links from 4 to 9%. Our results indicate kinetic competition between O/S exchange *versus* PTA insertion from Alk* and CS_2_*versus* PTA insertion from T. Furthermore, we find that the amount of thioester links remains constant when increasing the reaction temperature from 80 °C to 100 °C to 120 °C (Table 1, runs #9, #16, #17) confirming that O/S exchange is probably not in a thermally equilibrating process during ROTERP (ESI Fig. S15†). ROCOP between PTA and BO is also catalysed by LiOBn, which gave further insight into the ROTERP process. The produced polymers are colourless poly[(thio)ester]s featuring characteristic ester (*δ* = 165.8–167.5 ppm, *ν̃* = 1725 cm^−1^) and thioester (*δ* = 192.0–193.3 ppm, *ν̃* = 1670 cm^−1^) signals in NMR and IR (ESI Fig. S17–S21†). Again ^1^H–^13^C HMBC NMR spectroscopy reveals that thioesters sit adjacent to the secondary CH_2_R_2_ tail position of the ring opened BO while esters sit adjacent to the tertiary CHR_3_ head positions but in contrast to the ROTERP case no long-range order can be observed. The formed polymer features 60% monothioteraphthalate (*δ*(^13^C) = 192.1 and 166.6 ppm), 20% dithioterephthalate (*δ*(^13^C) = 192.8 ppm) and 20% terephthalate links (*δ*(^13^C) = 165.9 ppm). For this ROCOP we also propose propagation *via* alternating enchainment of PTA and BO alongside O/S exchange at the chain end. Note that if O/S exchange was quantitative (or completely absent) in PTA/BO ROCOP one would only observe the formation of monothioterephthalate links. The formation of dithioterephthalate and terephthalate links alongside monothioterephthalate links however necessitate incomplete O/S exchange and insertion of lithiumthiolates alike T into PTA (Fig. 4). This makes it likely that both these pathways also occur in ROTERP causing the formation of thioester errors (Fig. 3). Furthermore, Li catalysed PTA/BO ROCOP is also significantly slower than ROTERP (TOF(ROTERP) > 100 h^−1^ and TOF(ROCOP) = 2 h^−1^, see Table 1, runs #9 and #14) and we were also only able to prepare low molecular mass materials (*M*_n_ > 10 kg mol^−1^). Moving from ROCOP to ROTERP hence has real benefits in terms of reaction rate and product selectivity.

**Fig. 4:**
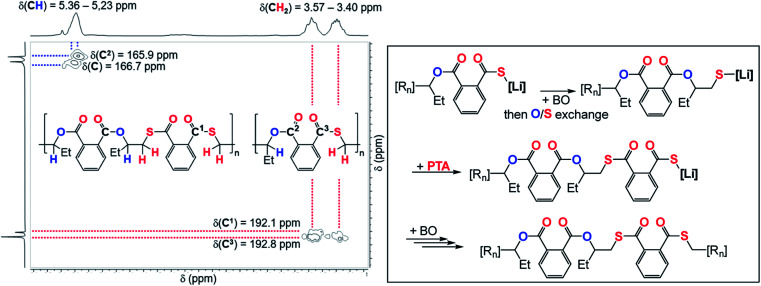
(Left) Selected region of the ^1^H–^13^C HMBC NMR spectrum (CDCl_3_, 25 °C, 500 MHz) of the polymer corresponding to Table 1, run #15. (Right) Proposed mechanism of the formation of dithioterephthalate and terephthalate links during PTA/BO ROCOP; [R_*n*_] = polymer chain.

As also seen in Table 1 there is some cyclic dithiocarbonate byproduct c5c formed during ROTERP and the amount remains constant irrespective of the reaction conditions. We infer that c5c could be formed *via* backbiting from Alk onto the adjacent trithiocarbonate links (Fig. 3) in which c5c elimination is favoured over O/S exchange. Accordingly attempted ROCOP between CS_2_ and BO at 80 °C exclusively yields c5c and no polymer (Table 1, run #14, ESI Fig. S22†). We observe the same reactivity after full PTA consumption in ROTERP where the lithium catalyst switches from terpolymer production to c5c formation (ESI Fig. S23†) and these results confirm that c5c is formed from backbiting reactions if PTA insertion does not occur. However, backbiting onto the trithiocarbonate links appears to be disfavoured in general as only small amounts of c5c (5% of the product mixture) are formed. In a related report on CS_2_ ROCOP, trithiocarbonate links have been observed to be the thermodynamic product of O/S scrambling suggesting that O/S exchange pathways that involve trithiocarbonates are thermodynamically unfavourable. The stability of the trithiocarbonate link could originate from resonance effects of the π-system which would also result in energetically less accessible π*-orbitals towards nucleophilic attack through backbiting.^27,53^

Clearly the O/S exchange reaction is crucial for the occurrence of ROTERP. To verify and further explore this isomerisation step, we synthesised a model intermediate for TC namely MTC from the stoichiometric reaction of LiO^*t*^Bu with PTA (Fig. 5) which instantaneously reacts in THF at room temperature similar to propagation step (i) in Fig. 3 (ESI Section S5†). To obtain structural insight we crystallised MTC from THF which is serving as a model donor in place of epoxides. Intriguingly single crystal X-ray analysis shows the formation of a dimer where two lithium thiocarboxylates come together to form a central Li_2_O_2_ motif *via* coordination of the Ar-C(=S)–O oxygen and coordinative saturation with two THF molecules per Li. The bimetallic nature of MTC is interesting in light of recent developments in ROCOP showing that multimetallic complexes are particularly active in this catalysis and the same could be true for ROTERP given the strong tendency for lithium salts to form aggregates in solution.^54–60^ Furthermore the sulphur centres remain uncoordinated by Li, presumably due to its comparatively high oxophilicity in the series of alkali metals.^61^ Hence the sulphur centres are sterically unencumbered which might aid propagation through nucleophilic attack by those. The yellow (C=S) chromophore is maintained in THF solution (*λ*_*Abs*_ = 350–430 nm, ESI Fig. S37†), however upon addition of excess BO gradual discolouration over the course of five minutes occurs. NMR analysis shows exclusive formation of ester containing products with no thioester resonances present (ESI Fig. S34†). ESI-MS identifies the reaction products as phthalic diester appended thioethers (ESI Fig. S36†). Our observations can be rationalised by insertion of MTC into BO *via* nucleophilic attack of the sulfur centre like step (ii) followed by O/S isomerisation as in step (iii) and consecutive insertion of the formed lithium thiolate into BO. The observed reactivity not only supports the mechanistic hypothesis outlined in Fig. 3 but also shows that lithium thiolates insert into BO which explains the formation of significant thioether links in PTA/BO ROCOP in absence of CS_2_. When MTC was reacted with substoichiometric (0.5 eq.) amounts BO to avoid thioether formation we observe clean formation of butylene thiirane and the corresponding carboxylate (Fig. 5 and ESI Fig. S32†). This reactivity also confirms step (ii) and (iii), whereas now the thiolate intermediate reacts under intramolecular nucleophilic substitution to form a thiirane and eliminates the adjacent ester link as a carboxylate. Hence, we suggest that free thiolate chain ends appended to ester groups such as T are short living intermediates during ROTERP and the fact that no thiirane is observed during ROTERP also supports this. A different outcome is observed when excess CS_2_ (10 eq.) is present during the reaction of 1 eq. BO with MTC (ESI Fig. S38 and S39†). We again only observe ester and no thioester containing products but also observe the initiation of CS_2_/BO ROCOP forming scrambled polythiocarbonate alongside c5c leaving 85% MTC unreacted. Hence the propagation steps that don't involve MTC appear to be faster than (ii) which makes this the presumably slowest propagation step of ROTERP (note that PTA reacts on the timescale of seconds with LiO^*t*^Bu while MTC insertion into BO occurs on the timescale of minutes to hours and that thiolates were found to be unstable towards thiirane elimination which also supports this notion). Furthermore, as we always observe quantitative O/S exchange, we suggest that this process is thermodynamically favoured and errors from incomplete O/S exchange during ROTERP are kinetic in origin.

**Fig. 5:**
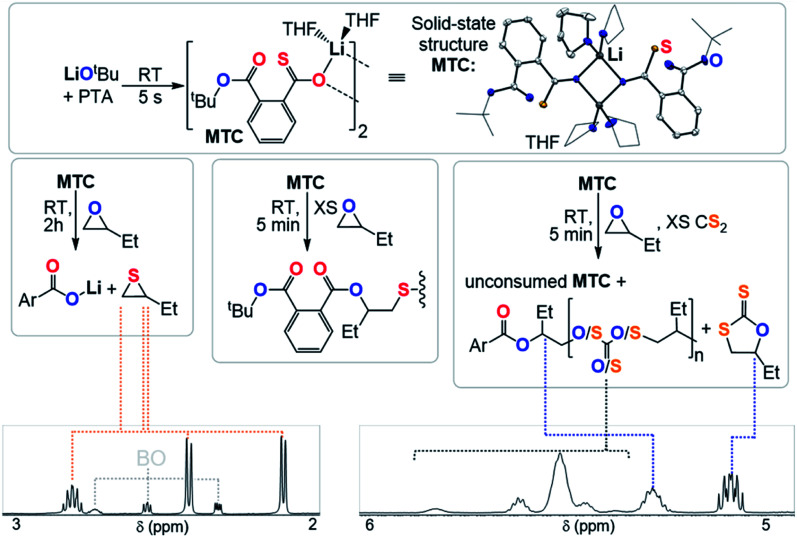
Mechanistic experiments with selected regions of the ^1^H NMR spectra (CDCl_3_, 25 °C, 400 MHz) supporting O/S exchange reaction and solid-state structure of MTC, hydrogen atoms omitted for clarity; white = C; blue = O, yellow = S, purple = alkali metal.

As outlined above the CS_2_ ROCOP literature shows that only lithium catalysts can control the O/S exchange process. To explore this for ROTERP we conducted terpolymerisation with NaOBn and KOBn in place of LiOBn (Table 1, runs #11 and #12). Intriguingly we find that NaOBn is slower (TOF 11 h^−1^*vs.* TOF > 50 h^−1^) than LiOBn and produces more thioester links (23% for Na *vs.* 4% for Li, Fig. 6 and ESI Fig. S16†). Employing KOBn results in no polymerisation at all. Intrigued by this striking difference in activity and selectivity for different alkali metals we conducted the analogous model experiments as outlined in the previous section with Na and K. Reaction of NaO^*t*^Bu and KO^*t*^Bu with PTA in THF at room temperature results in quantitative PTA ring-opening within seconds yielding the Na derivative MTC^Na^ and the K derivative MTC^K^. Both crystallise as extended networks as can be seen in Fig. 6. In contrast to the lithium derivative MTC, coordination of the thiocarboxylate sulfur centre as well as the adjacent ester carbonyl oxygen centre is also observed in the solid-state structures of MTC^Na^ and MTC^K^. While the alkali metal is four-coordinate in MTC as usually observed for Li, Na and K in MTC^Na^ and MTC^K^ are five and six coordinate. Although the precise structure in solution of these model intermediate remains to be determined, we still believe that because all structures were obtained under identical conditions (*i.e.* from THF/Pentane mixtures) our results highlight the greater tendency for the softer and larger alkali metals coordinated to the sulfur centres as well as the functionalities of the adjacent polymer chain. Hence, we propose that the rigid coordination sphere in addition to the high oxophilicity of lithium are responsible for its activity in ROTERP. We next reacted MTC^Na^ with 1 eq. BO in presence of excess CS_2_ in THF (Fig. 6, ESI Fig. S42†). In contrast to MTC which reacts within minutes with BO at room temperature, MTC^Na^ reacts with BO on the timescale of hours, and this reflects the reduced activity of Na in ROTERP compared to Li. NMR analysis of the product mixture reveals the formation of c5c (*δ*(^13^C) = 210.8 ppm) and a diester appended anionic trithiocarbonate (*δ*(^13^C) = 243.6 ppm) as the main reaction products alongside unconsumed MTC^Na^.^62,63^ As for Li, we only observe ester and no thioesters containing products also pointing towards quantitative O/S exchange for Na. In ROTERP however Na produced significantly more thioester links from incomplete O/S exchange and PTA insertion into thiolate chain ends. Hence our findings indicate that O/S exchange *versus* PTA insertion selectivity could be kinetically controlled by the metal catalyst. The observation of the anionic trithiocarbonate furthermore confirms steps (ii)–(iv) outlined in Fig. 3. Here we also proposed that c5c could be generated through backbiting reaction following BO insertion of the trithiocarbonate intermediate and this explains its formation in this model experiment. Unfortunately, MTC^K^ is only sparingly soluble in organic solvents which prevents reactivity studies. Combined our results show that LiOBn serves the role of a catalyst rather than a mere initiator and that the metal choice is crucial for controlling the O/S exchange process which likely occurs on a kinetic basis. Nevertheless, many questions remain unanswered, and a more detailed mechanistic study is currently underway.

**Fig. 6:**
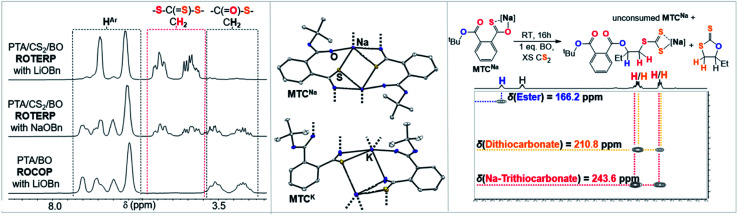
(Left) Selected regions of the ^1^H NMR spectra (CDCl_3_, 25 °C, 500 MHz) of polymers corresponding to the polymers obtained from Table 1, runs #9, #11 and #14. (Middle) Comparison of the solid-state structure of MTC^Na^ and MTC^K^. Colour code: white = C; blue = O, yellow = S, purple = alkali metal. (Right) Model reaction employing MTC^Na^ and associated ^1^H–^13^C HMBC NMR spectrum.

Next, we investigated some material properties of the new terpolymers (ESI Section S2†). The obtained materials are amorphous in nature (*T*_g_ = 33.7 °C, for Table 1 run #4; *T*_g_ = 22.6 °C, for Table 1 run #9) and show a good thermal stability (*T*_d,5%_ = 238.6 °C, for Table 1 run #4, *T*_d,5%_ = 235.4 °C, for Table 1 run #9). Note that is significantly higher than that of the of the parent CS_2_/PO ROCOP poly(thiocarbonate-*alt*-trithiocarbonate) (*T*_d,5%_ = 117.9 °C) and we suggest that this is because the heterocarbonate units in the poly(ester-*alt*-ester-*alt*-trithiocarbonate) are separate by terephthalate units which prevents the occurrence of depolymerisation *via* backbiting pathways.^64–66^ The polymers show excellent solubility in organic solvents (THF, DCM, CHCl_3_) even at high molecular mass, an attractive processing property, and the high molecular mass materials (Table 1, runs #5 and #6) can be solvent casted into self-standing films from DCM. This contrasts the behaviour of the related polymers only comprising thiocarbonate groups, poly(trithiocarbonates), which were reported to only exhibit poor solubility.^20^ The terpolymers can be formally considered polyesters with regularly distributed trithiocarbonate links and this should result in properties typical for sulfur containing polymers such as susceptibility to oxidation and photolysis.^25,36,67,68^ Indeed, we find that irradiation of the ROTERP polymer with broadband UV light or dispersion in H_2_O_2_ leads to selective cleavage of the trithiocarbonate groups (99% cleavage for 16 h UV irradiation or 5 d dispersion in aqueous H_2_O_2_) and degrades the material into oligomers with *M*_n_ < 1 kDa (ESI Section S7†). The ^1^H NMR spectra of the product mixtures after degradation show the complete disappearance of the CH_2_R_2_-trithiocarbonate groups at *ca.* 3.75 ppm while CHR_3_-ester groups at *ca.* 5.5 ppm can still be detected. To further investigate whether degradability stems from the trithiocarbonate links we prepared a related polyester (without interspersed trithiocarbonate) poly(propylene-orthoterphthalate) *via* phthalic anhydride/PO ROCOP, and this polymer shows no appreciable degradation under the same conditions. This might imply that there are some degradability benefits to ROTERP polymers over more conventional polyesters as photolysis and oxidation represent the initial breakdown pathways of polymer waste in nature.^3^ The ROTERP polymer shows furthermore a good refractive index of 1.62 which is similar to that of the parent ROCOP polymers (1.60 for PTA/PO ROCOP polymer and 1.70 for CS_2_/PO polymer) and this is also typical for sulphur containing polymers.^37^

Having established that ROTERP is a useful methodology for material synthesis we were intrigued to see whether it also allows the synthesis of more complex block polymers structures. In ROCOP, the concept of switchable catalysis has been established as a mechanistically elegant and practical tool to synthesise block-polymers with useful material properties.^69–73^ Here a suitable catalyst first mediates the ROP of for example cyclic esters with epoxides present in the mixture until the second ROCOP monomer (*e.g.* CO_2_) is added causing immediate termination of ROP and the onset of (*e.g.* CO_2_/epoxide) ROCOP to form a ROCOP block connected to the ROP polymer.^74^ As ROTERP formally derives from ROCOP we hypothesised that switchable catalysis between ROP and ROTERP might be possible.

To investigate this concept, we first had to identify a suitable ROP that is also mediated by LiOBn in epoxide solvent to then proceed to mechanistic switching. We found that ε-decalactone (εDL) smoothly undergoes living LiOBn catalysed ROP in BO as the solvent without any epoxide ring-opening occurring alongside εDL ROP (ESI Fig. S45–S48†). The polymerisation follows a first order rate law with respect to εDL consumption and an excellent initial TOF of 490 h^−1^ (at 1 eq. LiOBn : 100 eq. εDL : 500 eq. BO and 25 °C) yielding narrow (*Đ* < 1.2) poly(decalactone) (PDL). Addition of CS_2_ (500 eq. per LiOBn) and PTA (70 eq.) to polymerising εDL (50 eq) in BO (250 eq.) after 15 min at room temperature completely and immediately stops the occurrence of εDL ROP (Fig. 7). Heating to 80 °C initiates ROTERP and a poly(ester-*alt*-ester-*alt*-trithiocarbonate) block grows from the PDL-chain-end until the reaction is stopped after 30 min. Following the polymerisation progress by ^1^H NMR over time shows the formation of OBn initiated PDL which is followed by a ROTERP block forming uniformly in the second phase of the polymerisation as for the stand-alone ROTERP reactions discussed above. Under these conditions ROTERP occurs in 98% linkage selectivity with 2% erroneous thioester links.

**Fig. 7:**
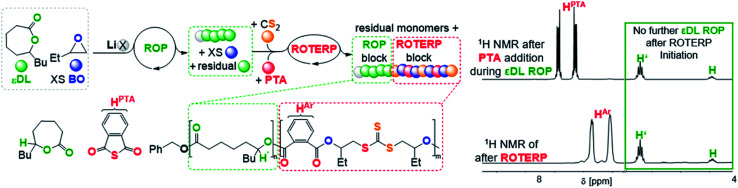
εDL ROP to ROTERP switchable catalysis sequence and ^1^H NMR spectra (CDCl_3_, 25 °C, 400 MHz) of aliquots removed at different stages of switchable catalysis. X = OBn.

Switchable catalysis and block polymer formation were established by a combination of analytical methods: (i) no εDL is consumed during ROTERP (Fig. 7) and the ^13^C{^1^H} PDL C=O resonance at *δ* = 173.2 ppm remains unaffected by the ROTERP process (ESI Fig. S53†) showing the cessation of ROP and the absence of transesterification processes between blocks; (ii) the number averaged molecular mass shifts from *M*_n_ = 8.40 to 22.69 kDa (Fig. 8), which shows the growth of existing chains rather than the initiation of new ones. This increase in *M*_n_ furthermore fulfils statistical considerations for blockpolymer formation;^75^ (iii) ^31^P end group analysis shows the consumption of all PDL end groups (ESI Fig. S55†);^76^ (iv) the composition of the resulting block-polymer remains unchanged through multiple precipitations from DCM/MeOH and THF/pentane supporting that the blocks are joint; (vi) DSC analysis shows two *T*_g_'s at −45.7 °C for the ROP block and 26.2 °C for the ROTERP block suggesting microphase separation in the solid state;^77^ (vii) TGA analysis shows a stepwise thermal decomposition profile with two *T*_d,onset_ at approximately 205.0 °C for the ROTERP block and 300 °C for the ROP block (both Fig. 8). Previously reported switchable catalyses are associated with a change in the catalytic resting state as shown *via in situ* UV-VIS and NMR.^47,55,78^ This is a prerequisite as any active alkoxide chain ends present during ROCOP would lead to the occurrence of ROP. Similarly, we find for our new switches starting from ROP that the addition of the ROTERP monomers causes the immediate emergence of VIS bands at *ca.* 440 nm prior to any polymer formation (visible as a yellow discolouration of the previously colourless mixture, ESI Fig. S56 and S58†). This band is diagnostic for the (C=S) chromophore and likely due to the formation of thiocarboxylates. Another indicator for a transformation of the chain-ends is observed in the *in situ*^7^Li NMR spectra which is sharpening and shifting by 0.2 ppm upon comonomer addition to polymerising εDL in BO (ESI Fig. S57†) and both findings substantiate a change of the catalytic resting state. Together our experiments suggest successful switchable catalysis and block-polymer formation *via* mechanistic switching from ROP to ROTERP.

**Fig. 8:**
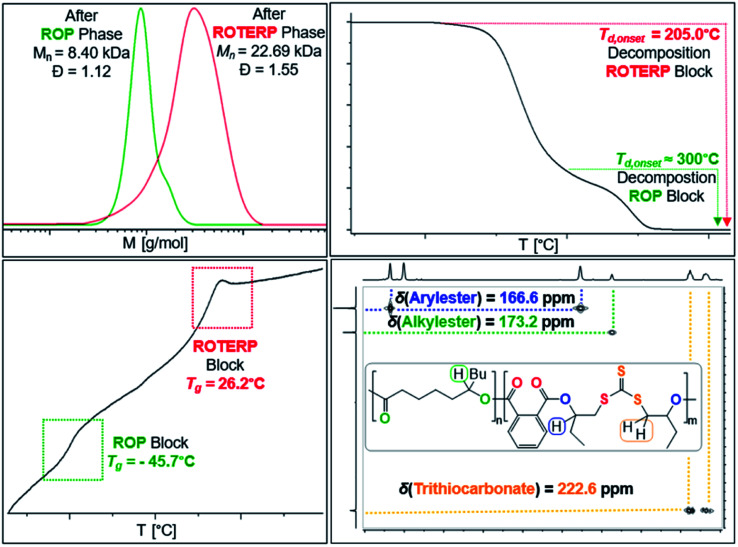
Overlayed SEC traces (top left) before and after switch as well as TGA (top right), DSC (bottom left) and ^1^H–^13^C HMBC (CDCl_3_, 25 °C, bottom right) of the obtained block-polymer.

In conclusion, we have expanded the repertoire of heteroatom containing polymerisation methodologies by sequence selective ring-opening terpolymerisation (ROTERP). Here three monomers, propylene/butylene oxide A, phthalic thioanhydride B, and CS_2_ C are enchained by a simple lithium catalyst in an (ABA′C)_*n*_ fashion. We obtained poly(ester-*alt*-ester-*alt*-trithiocarbonate)s with molecular masses of up to >10^5^ g mol^−1^ that are not easily accessible through other polymerisation methodologies. This unusual insertion selectivity is enabled by a central O/S exchange reaction at the polymer chain-end and we could confirm this hypothesis in model reactions. Lithium is key to achieving high selectivity and activity due to its' oxophilicity and rigid coordination sphere. Mechanistic experiments also indicate that ROTERP is a kinetically controlled process. With respect to the material properties, we found that incorporation of trithiocarbonate links renders these polymers oxidatively and photodegradable, while showing enhanced thermal stability and solubility compared to some of the related ROCOP polymers. Finally, we demonstrated, that ROTERP is mechanistically compatible with εDL ROP enabling mechanistic switching from ROP to ROTERP for blockpolymer synthesis. We believe that ROTERP is a generalizable methodology with many more viable monomer combinations to be discovered that lead to sequence selective rather than statistical terpolymerisation. ROTERP bears further promise as it can be more selective and faster than the respective ROCOPs. The methodology is mechanistically compatible with ROP and hence can be used for blockpolymer synthesis yielding chemically complex polymer architecture with tuneable material properties. Such materials are now more in demand than ever before given the sustainability challenges our current polymer economy is facing.

## Author contributions

A. J. P. designed the project . All authors jointly performed the experiments and wrote the manuscript.

## Conflicts of interest

There are no conflict of interests.

## Supplementary Material

SC-013-D2SC01776H-s001

SC-013-D2SC01776H-s002
