# Supplementary material for: Lithium achieves sequence selective ring-opening terpolymerisation (ROTERP) of ternary monomer mixtures
Source: Chem Sci. 2022 May 5;13(21):6355–65. doi: 10.1039/d2sc01776h (PMC9159086; doi:10.1039/d2sc01776h)
Supplement: SC-013-D2SC01776H-s001 [file SC-013-D2SC01776H-s001.pdf]

Electronic Supplementary Material (ESI) for Chemical Science.
This journal is © The Royal Society of Chemistry 2022

Lithium achieves sequence selective ring-opening terpolymerisation (ROTERP) of ternary monomer mixtures

Susanne Rupf,[a,#] Patrick Pröhm,[a,#] Alex J. Plajer [a,*]

[a] Intitut für Chemie und Biochemie, Freie Universität Berlin, Fabeckstraße 34-36, 14195 Berlin; [#] equal contribution; *email: plajer@zedat.fu-berlin.de

Table of contents

Section S1: Methods.......... 3
Section S2: ROTERP procedure and details.......... 4
Section S3: Polymer characterisation data 5
Section S4: SEC traces.......... 18
Section S5: Synthesis, characterisation and reactivity of model MTC 21
Section S6: Switchable Catalysis.......... 32
Section S7: Degradation Experiments 40
Section S8: Crystallography 43
Section S9: Bibliography 46

Section S1: Methods

Solvents and reagents were obtained from commercial sources and used as received unless stated otherwise. If “dried solvents” were used these were obtained by different procedures. Toluene, EtOH, n-hexane, n-pentane and CH_2Cl_2 were prepared by using an MBraun Solvent Purification System MB-SPS 800 filled with Al_2O_3. Et_2O was dried over Na/benzophenone and THF was dried over K/benzophenone under argon. The $CDCl_3$ was dried over CaH_2 and d_8-THF over sodium prior to vacuum transfer onto 4 Å sieves followed by three freeze pump thaw degassing cycles. ^1H, $^{13}C\{^1H\}$, and ^7Li NMR spectra were recorded by using a Jeol JNM-ECA 400II, Bruker Advance 600 and 700 MHz spectrometer. ^1H and $^{13}C\{^1H\}$ chemical shifts are referenced to the residual proton resonance of the deuterated solvents and $^7Li\{^1H\}$ chemical shifts are referenced to an internal reference capillary containing LiO_3SCF_3 in d_6-acetone (alongside a PPh_3 ^{31}P reference and PhF ^{19}F reference).

Epoxides, CS_2 and ε-DL were dried over calcium hydride at room temperature for 3 days, vacuum transferred (for CHO and ε-DL fractionally distilled under static vacuum) followed by three freeze pump thaw degassing cycles and stored inside an argon filled glovebox prior to use. Phtalic thioanhydride was synthesized according to the literature procedure and (for runs Table 1 #5 and #6) this was then purified by recrystallisation from ^{t}BuOMe followed by recrystallisation from $CHCl_3$ and two sublimation under dynamic vacuum at 90°C and was stored inside an argon filled glovebox prior to use.[1]

UV-visible spectra were collected on a Varian Cary 50 UV spectrometer.

Infrared spectra were measured using a Thermo-Nicolet Nexus 670 FTIR spectrometer with DuraSampl IR accessory in total reflection at room temperature.

The molecular mass and polydispersity of the polymers were determined by a Waters 1515 size exclusion chromatography (SEC) instrument equipped with two linear PLgel columns (Mixed-C) following a guard column and a differential refractive index detector using tetrahydrofuran as the eluent at a flow rate of 1.0 mL/min at 30 °C and a series of narrow polystyrene standards for the calibration of the columns. Each polymer sample was dissolved in HPLC-grade THF (6 mg/mL) and filtered through a 0.20 μm porous filter frit prior to analysis.

Polymer films for refractive index determination were spun from a Vacuum Spin Coater (SCE 150). 100 μL of terpolymers in CH_2Cl_2 (20 mg/mL) was dropped onto a silicon slice and the speed of the spin coater was then raised to 3000 r/min lasting for 1 min, affording a film of approximately 100 – 200 nm. Spectroscopic ellipsometry (SE) was performed on a SENpro ellipsometer (wavelength 350 – 1050 nm) and evaluated with SpectraRay 3 (Version 5.3.2.1853) software from SENTECH instruments GmbH (Berlin, Germany).

High-resolution ESI mass spectra were obtained using a Waters UPLC-Synapt G2-S HDMS.

Section S2: ROTERP procedure and details

Terpolymerization protocol: In an argon-filled glovebox, LiHMDS (an optionally BnOH) was dissolved in butylene oxide and the mixture was transferred to a flame dried vial equipped with a flame dried stirrer bar and sealed with a melamine cap containing a Teflon inlay. CS_2 and PTA were then added and the vial was brought outside the glovebox and placed in a pre-heated oil bath at 80°C for the specified time. In cases where the reaction progress was monitored, the vial was rapidly cooled to room temperature with a dry ice cooling bath and brought inside an argon-filled glovebox before the aliquot was remove. At the specified end point of the reaction, the polymer was isolated by adding the polymerisation mixture to 200 mL of acidified MeOH (20 µL concentrated HCl per 200 mL MeOH) resulting in the precipitation of the yellow product. After two further precipitations from DCM/MeOH, the polymer was isolated by filtration and dried in a vacuum oven set to 60 °C.

Section S3: Polymer characterisation data

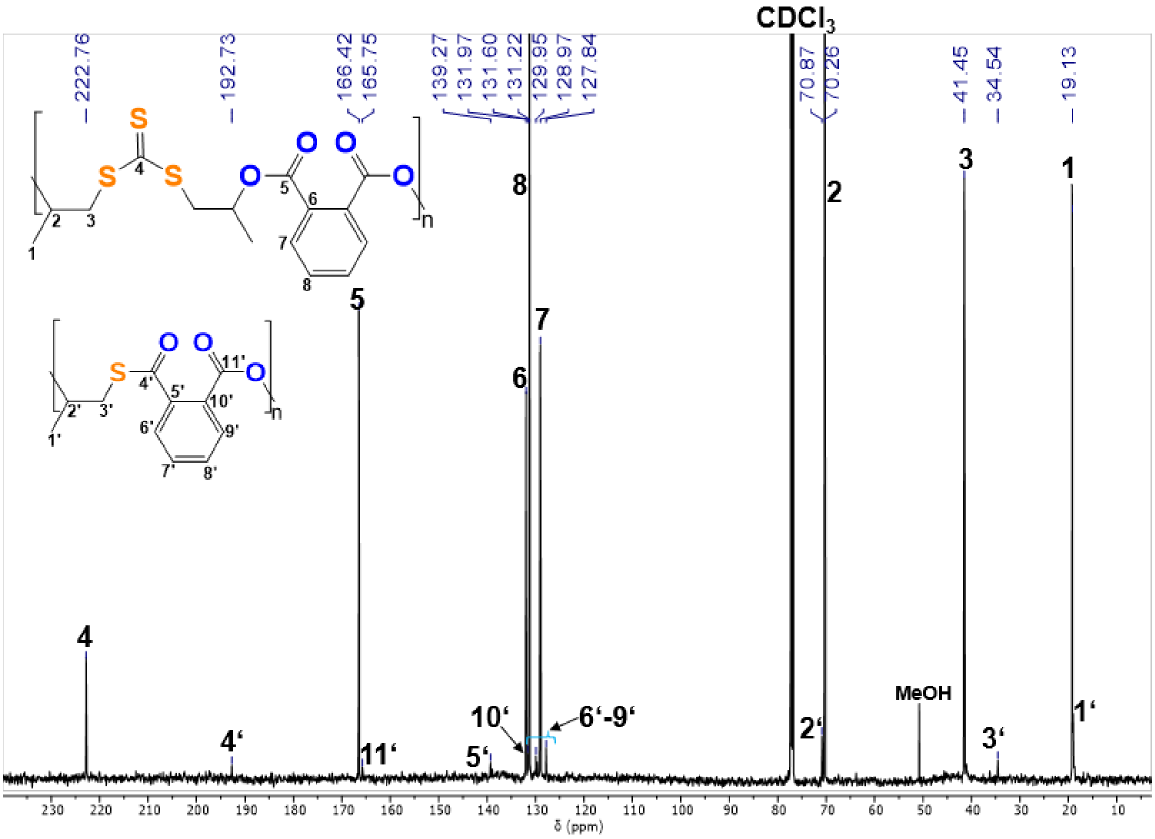

Figure S 1: $^{13}C\{^1H\}$ NMR spectrum (126 MHz, $CDCl_3$, 25°C) of terpolymer corresponding to table 1, run #3.

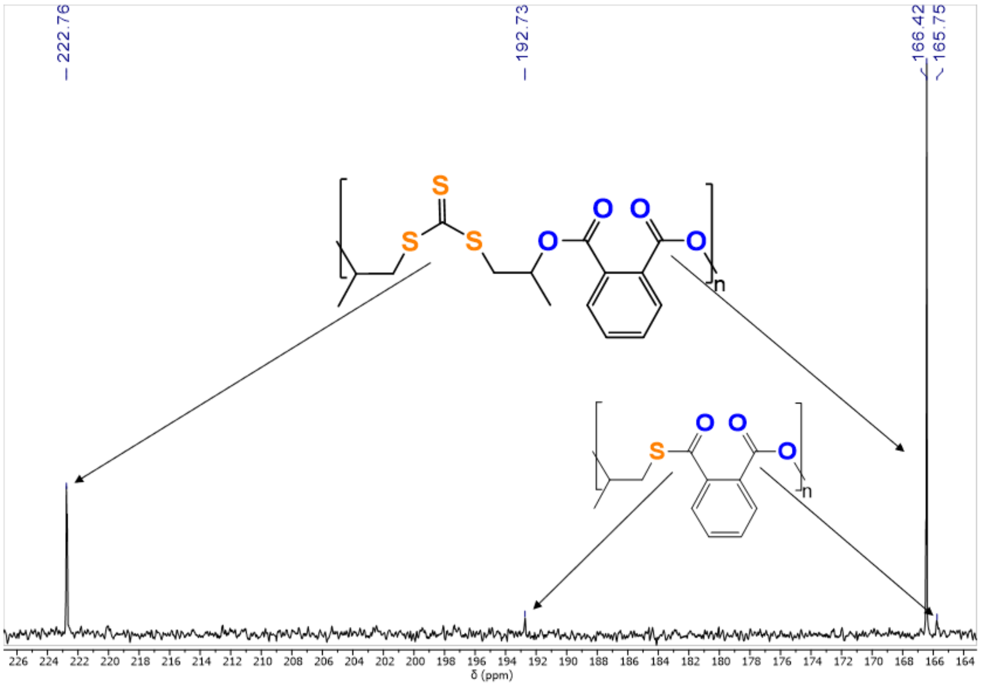

Figure S 2: Zoom into carbonyl region of the $^{13}C\{^1H\}$ NMR spectrum (126 MHz, $CDCl_3$, 25°C) of terpolymer corresponding to table 1, run #3.

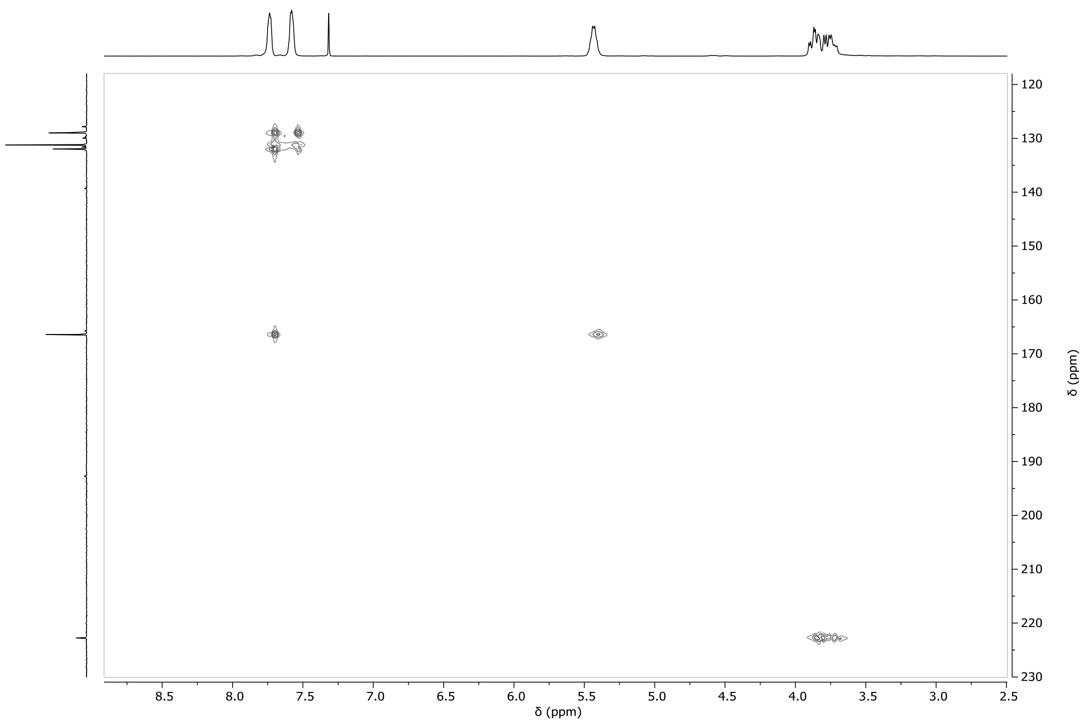

Figure S 3: ^{1}H – ^{13}C HMBC NMR spectrum ($CDCl_3$, 25°C) of terpolymer corresponding to table 1, run #3.

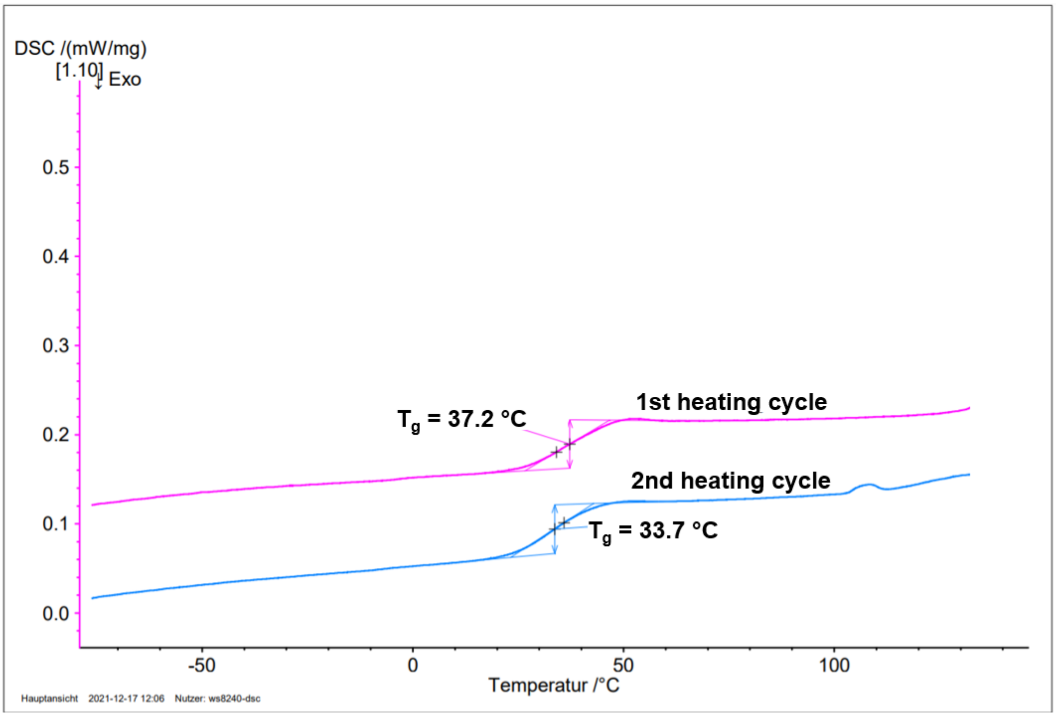

Figure S 4: DSC heating curve of terpolymer corresponding to table 1, run #4.

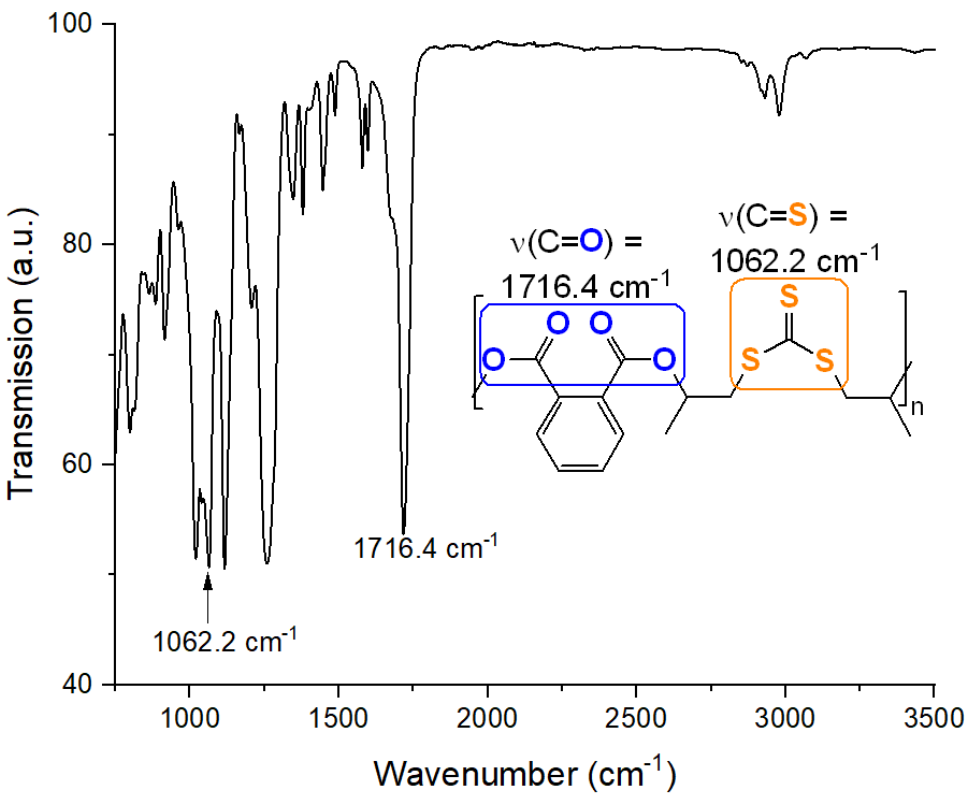

Figure S 5: FT ATRIR Spectrum of PTA/BO/CS_2 terpolymer corresponding to table 1, run #4.

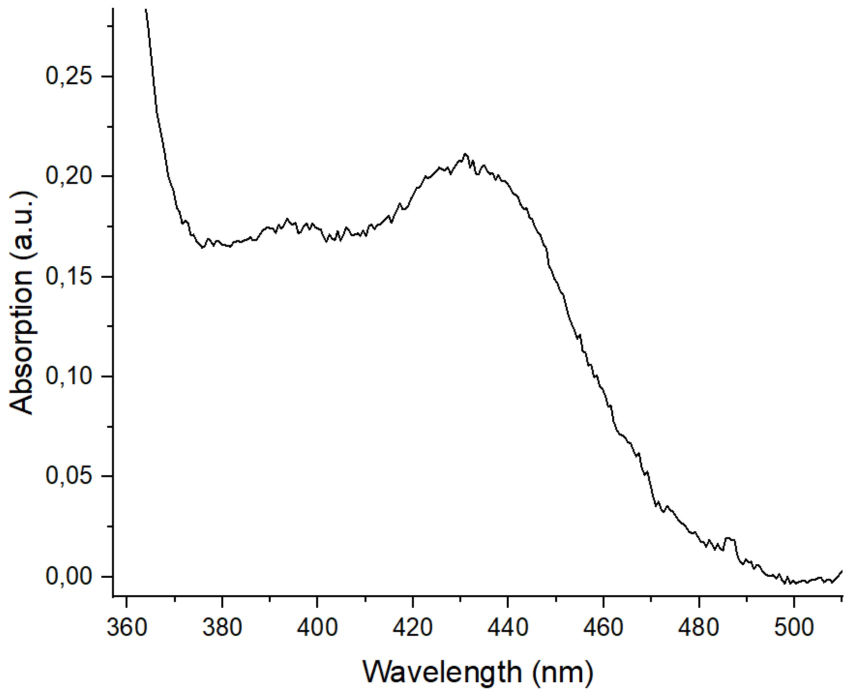

Figure S 6a: Zoom into the UVVIS spectrum (DCM, 25°C) of terpolymer corresponding to table 1, run #4.

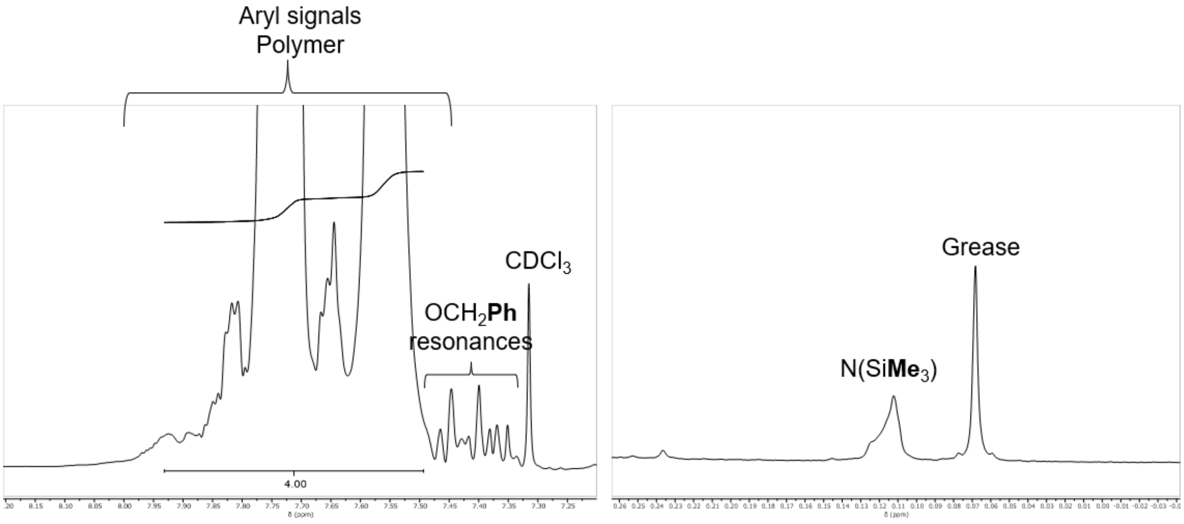

Figure S 6b: Zoom into the end-group resonances of of (left) a ROTERP reaction initiated from LiOBn and (right) initiated from LiHMDS.

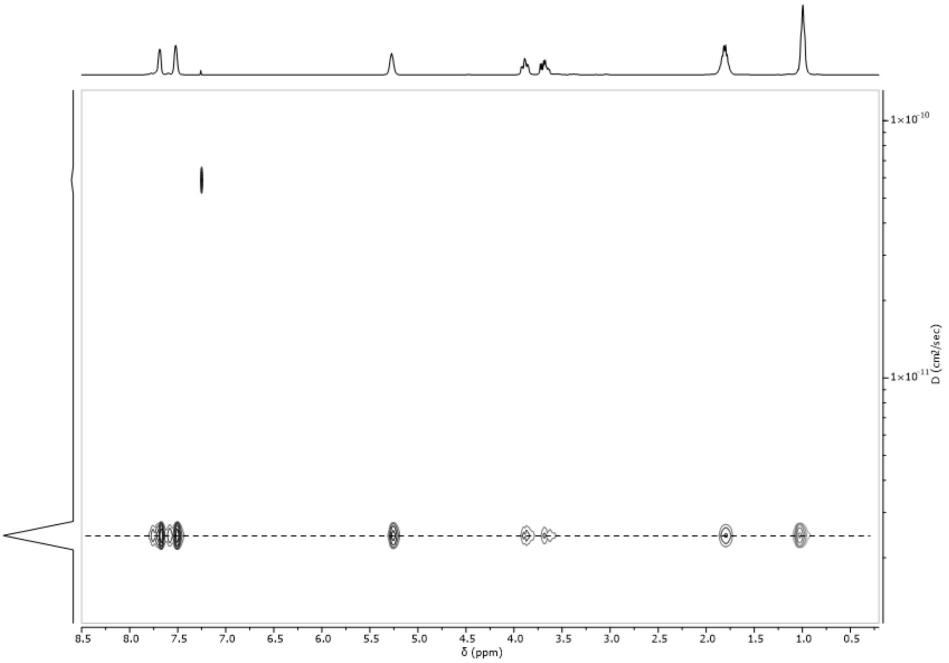

Figure S 6c: DOSY NMR spectrum (126 MHz, $CDCl_3$, 25°C) of terpolymer corresponding to table 1, run #3

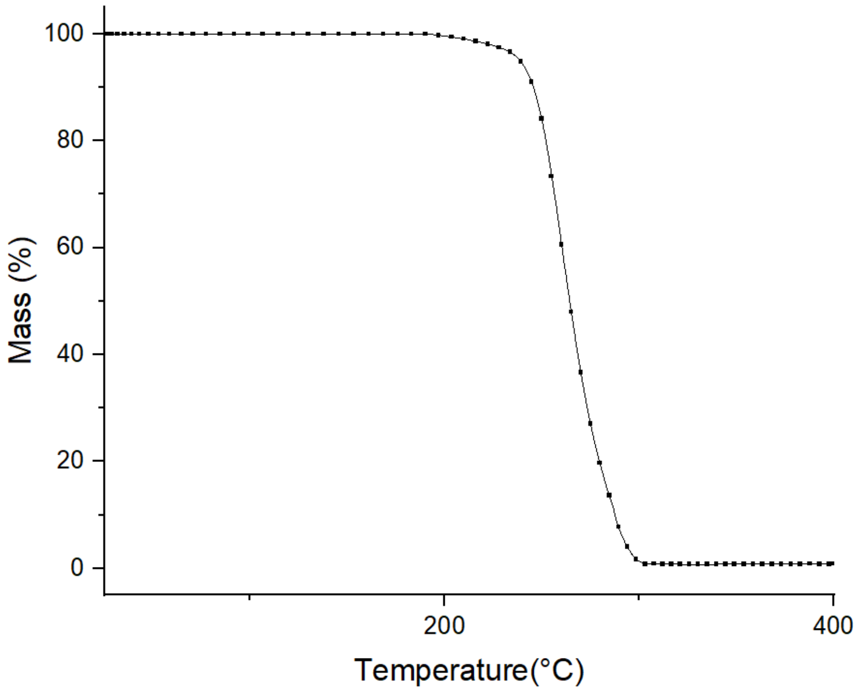

Figure S 7: TGA of terpolymer corresponding to table 1, run #4.

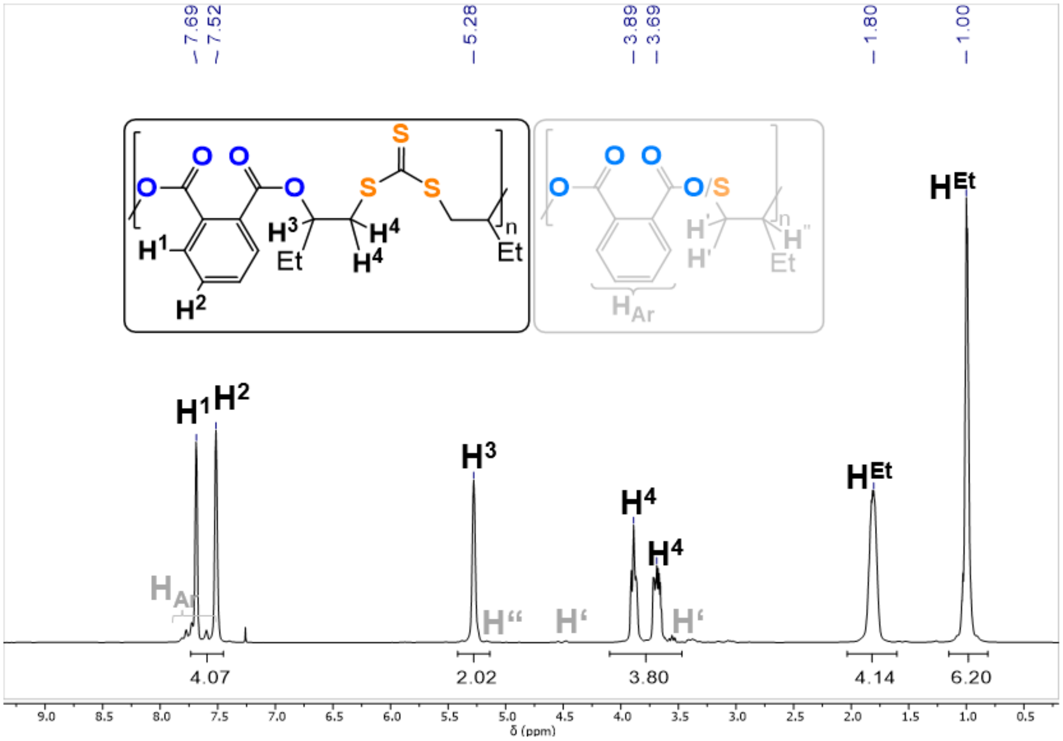

Figure S 8: ^{1}H NMR spectrum (400 MHz, $CDCl_3$, 25°C) of terpolymer corresponding to table 1, run #9.

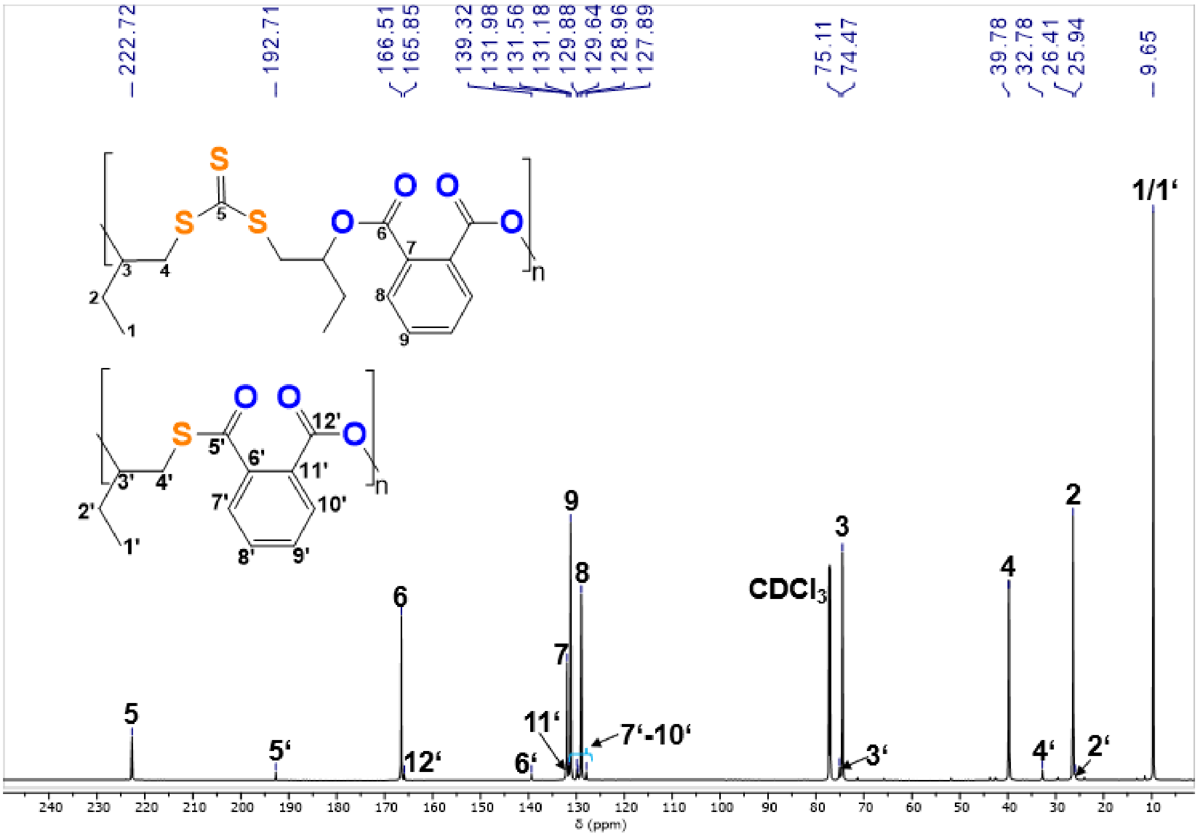

Figure S 9: $^{13}C\{^{1}H\}$ NMR spectrum (126 MHz, $CDCl_3$, 25°C) of terpolymer corresponding to table 1, run #9.

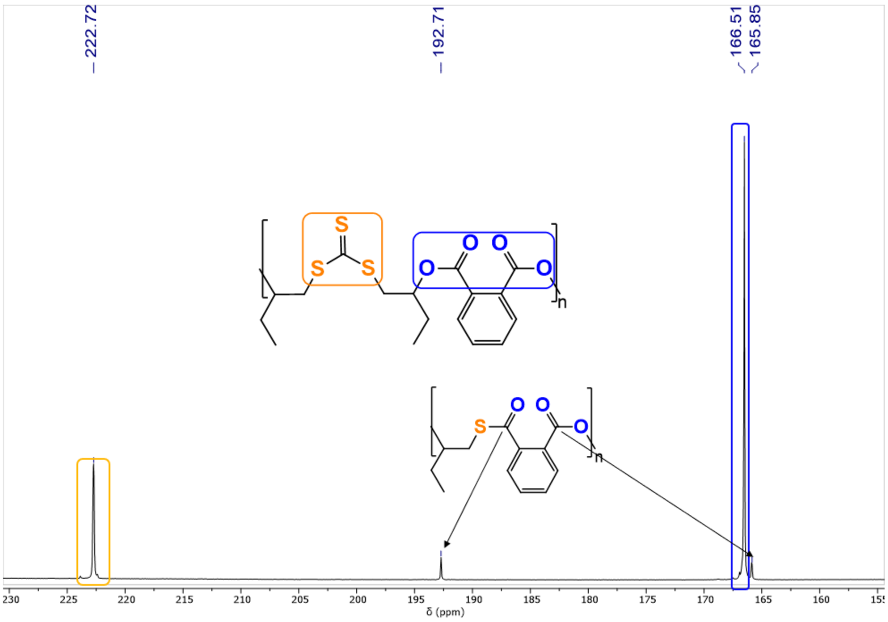

Figure S 10: Zoom into carbonyl region of the $^{13}C\{^{1}H\}$ NMR spectrum (126 MHz, $CDCl_3$, 25°C) of terpolymer corresponding to table 1, run #9.

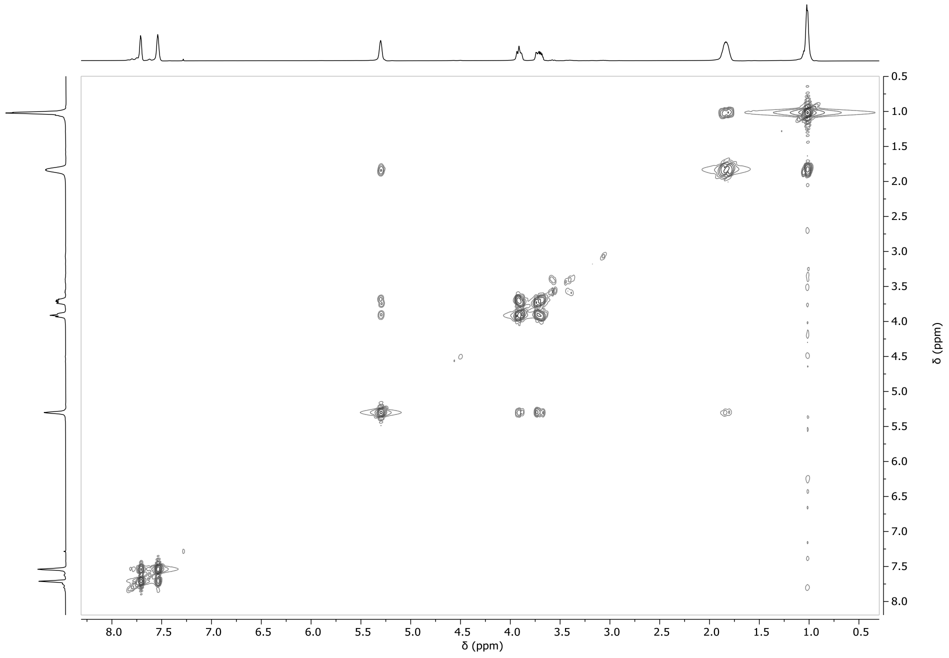

Figure S 11: ^{1}H – ^{1}H COSY NMR spectrum ($CDCl_3$, 25°C) of terpolymer corresponding to table 1, run #9.

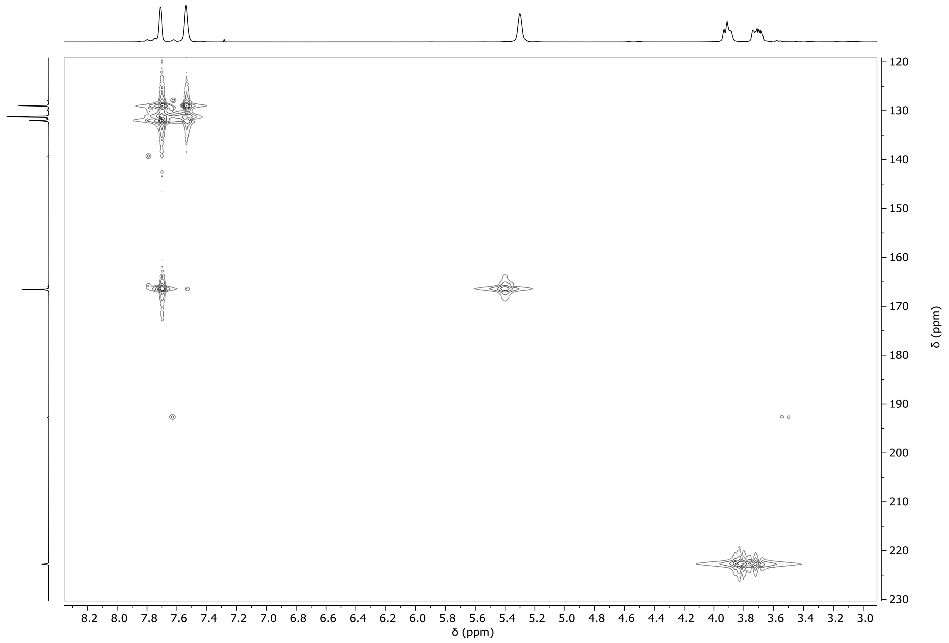

Figure S 12: ^{1}H – ^{13}C HMBC NMR spectrum ($CDCl_3$, 25°C) of terpolymer corresponding to table 1, run #9.

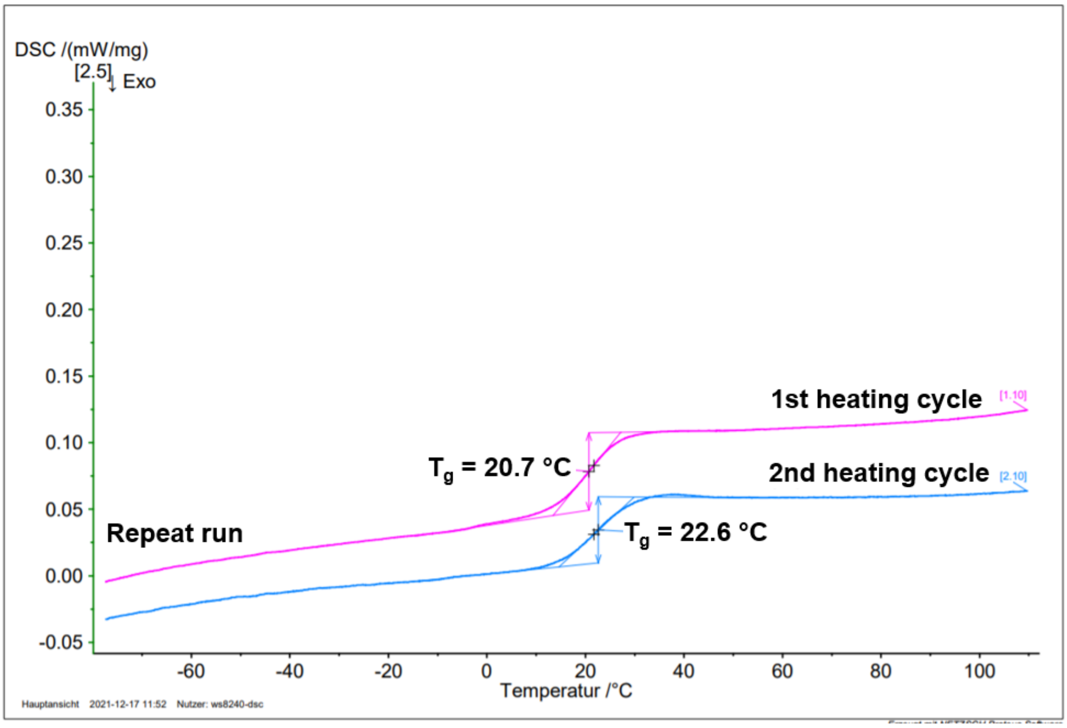

Figure S 13: DSC heating curve of terpolymer corresponding to table 1, run #9.

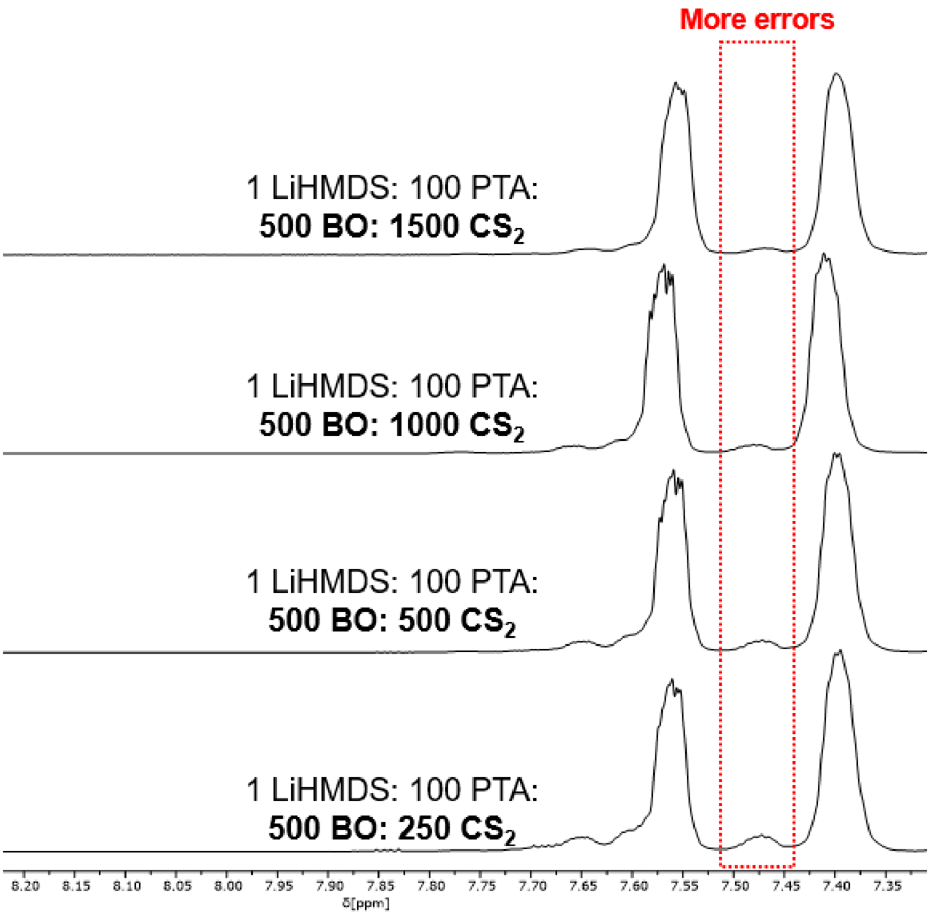

Figure S 14: Comparison of the aromatic region of the ^{1}H NMR spectra (400 MHz, $CDCl_3$, 25°C) of terpolymers corresponding to table 1, run #7 to #10.

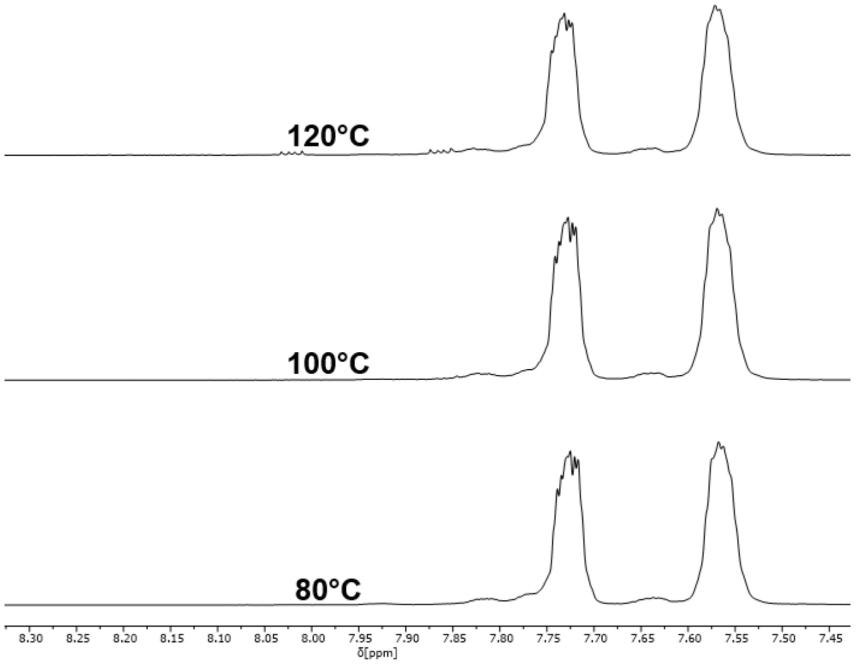

Figure S 15: Comparison of the aromatic region of the ^{1}H NMR spectra (400 MHz, $CDCl_3$, 25°C) of crude terpolymers obtained at different reaction temperatures (loading as per Table 1 run #9.

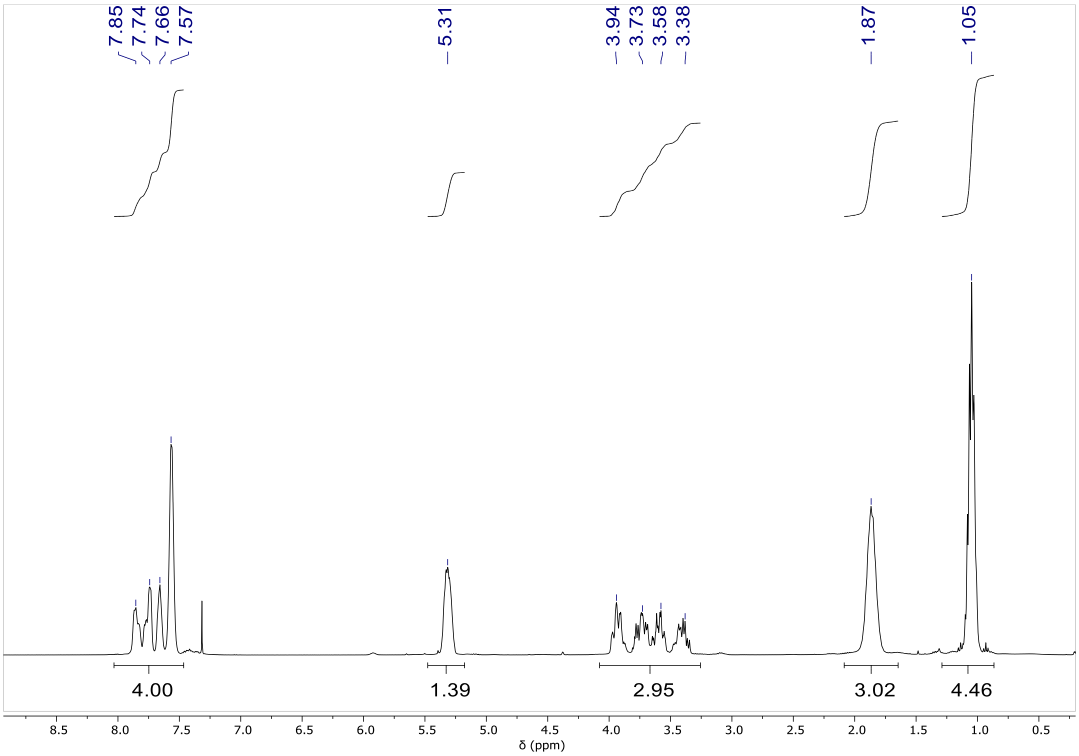

Figure S 16: ^{1}H NMR spectra (400 MHz, $CDCl_3$, 25°C) of terpolymers corresponding to Table 1 run #12.

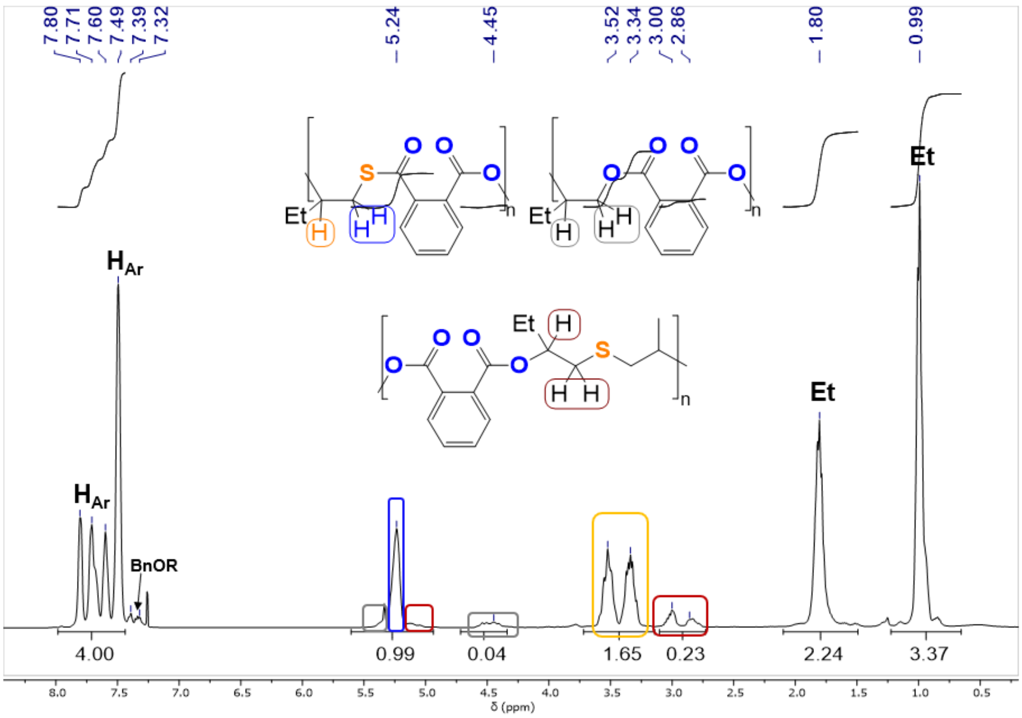

Figure S 17: ^{1}H NMR spectrum (400 MHz, $CDCl_3$, 25°C) of copolymer corresponding to table 1, run #15.

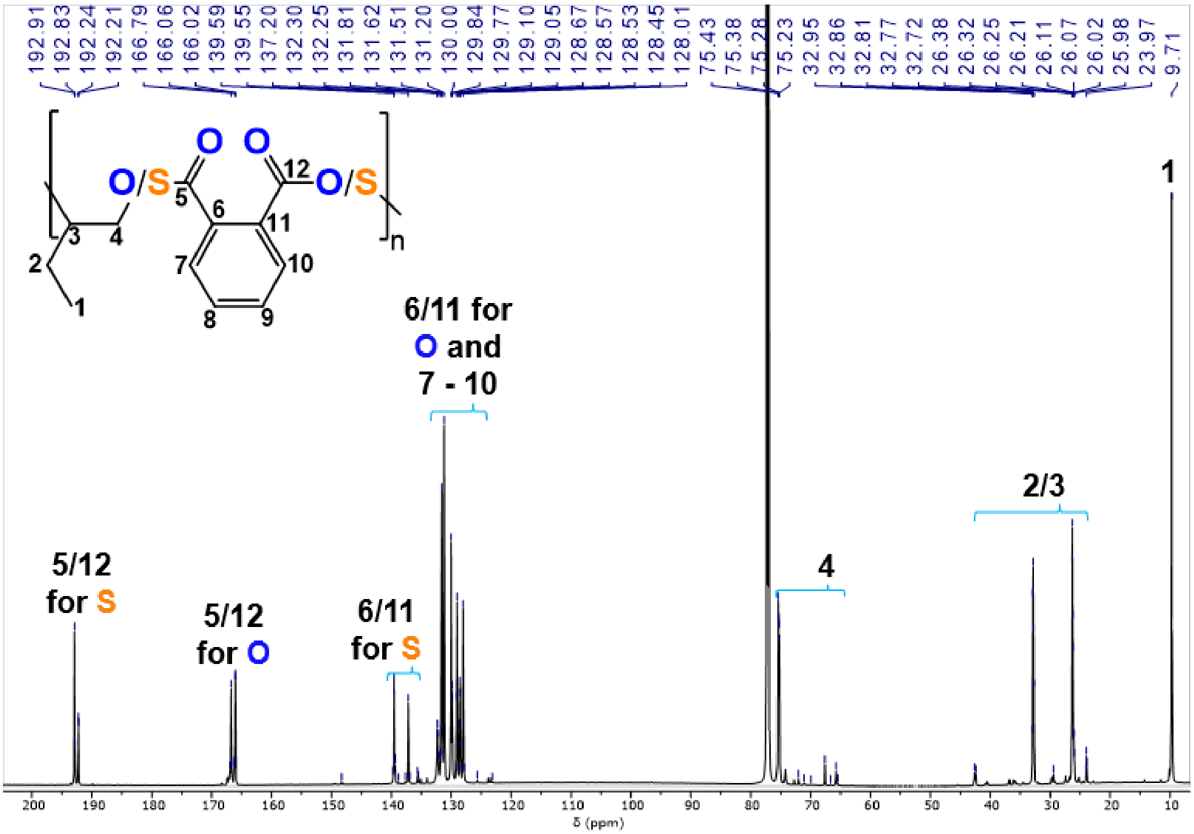

Figure S 18: $^{13}C\{^{1}H\}$ NMR spectrum (126 MHz, $CDCl_3$, 25°C) of copolymer corresponding to table 1, run #15. Due to significant transesterification and O/S

exchange process as well as the presence of ether linkages unambiguous assignment was not possible.

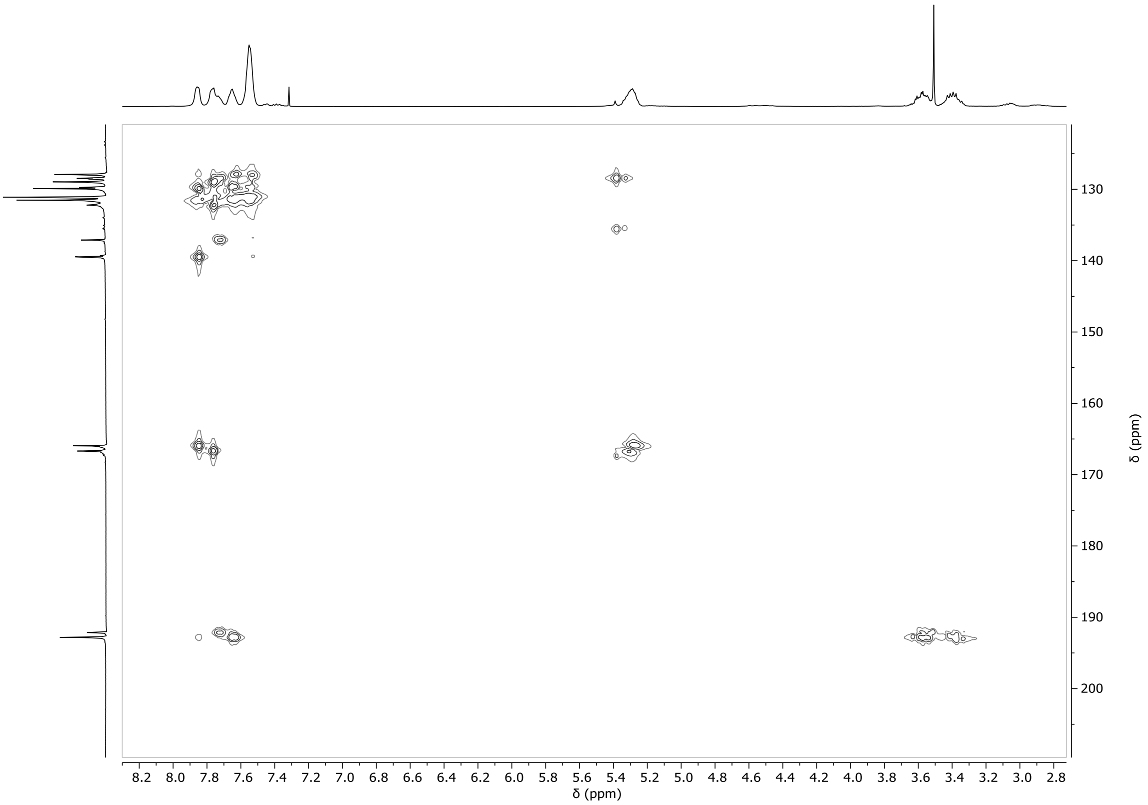

Figure S 19: ^{1}H - ^{13}C HMBC NMR spectrum ($CDCl_3$, 25°C) of copolymer corresponding to table 1, run #15.

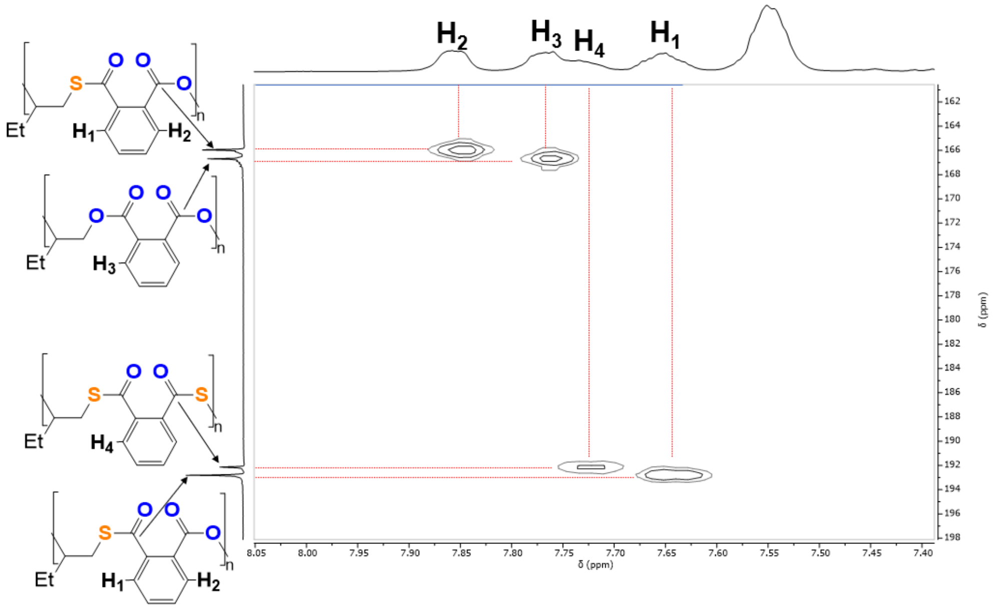

Figure S 20: Zoom into the ^{1}H - ^{13}C HMBC NMR spectrum ($CDCl_3$, 25°C) of copolymer corresponding to table 1, run #15.

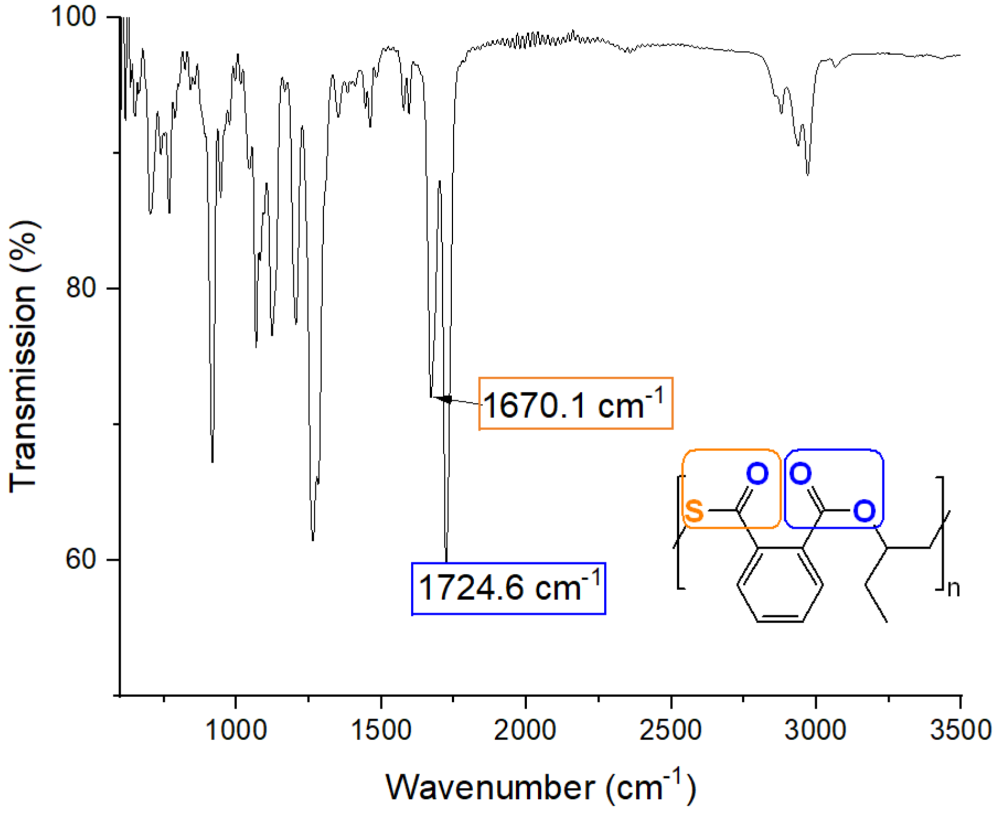

Figure S 21: FT ATR-IR spectrum of copolymer corresponding to table 1, run #9.

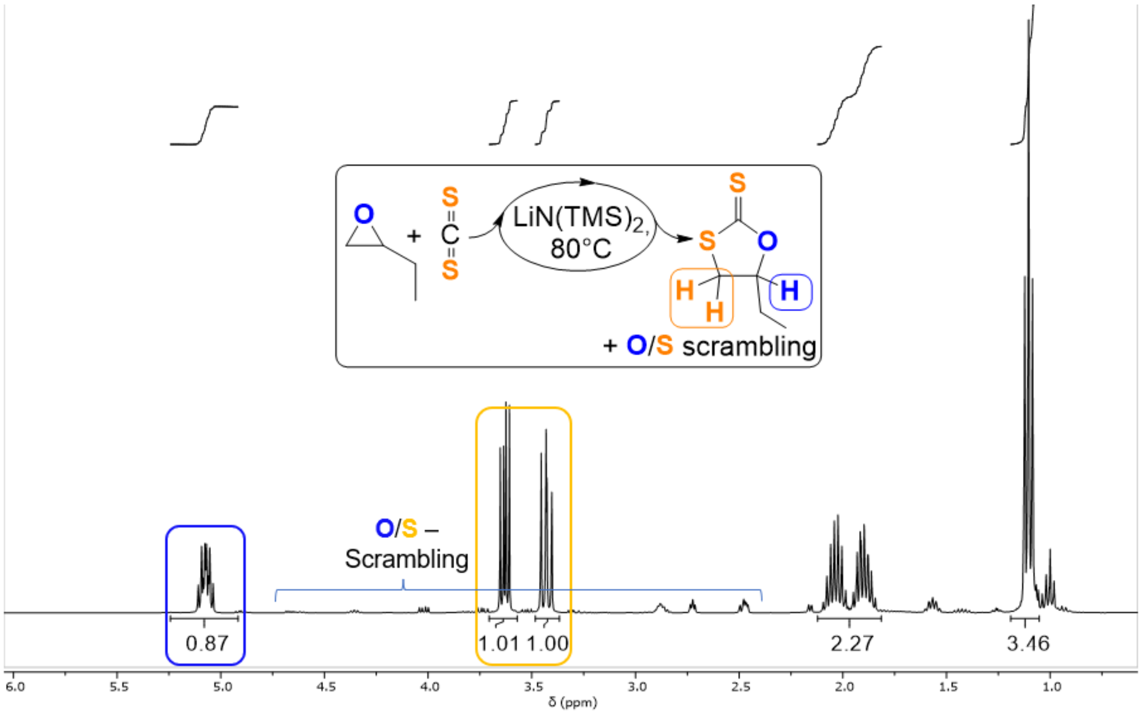

Figure S 22: ^{1}H NMR spectrum (400 MHz, $CDCl_3$, 25°C) of reaction mixture produced according to Table 1, #14.

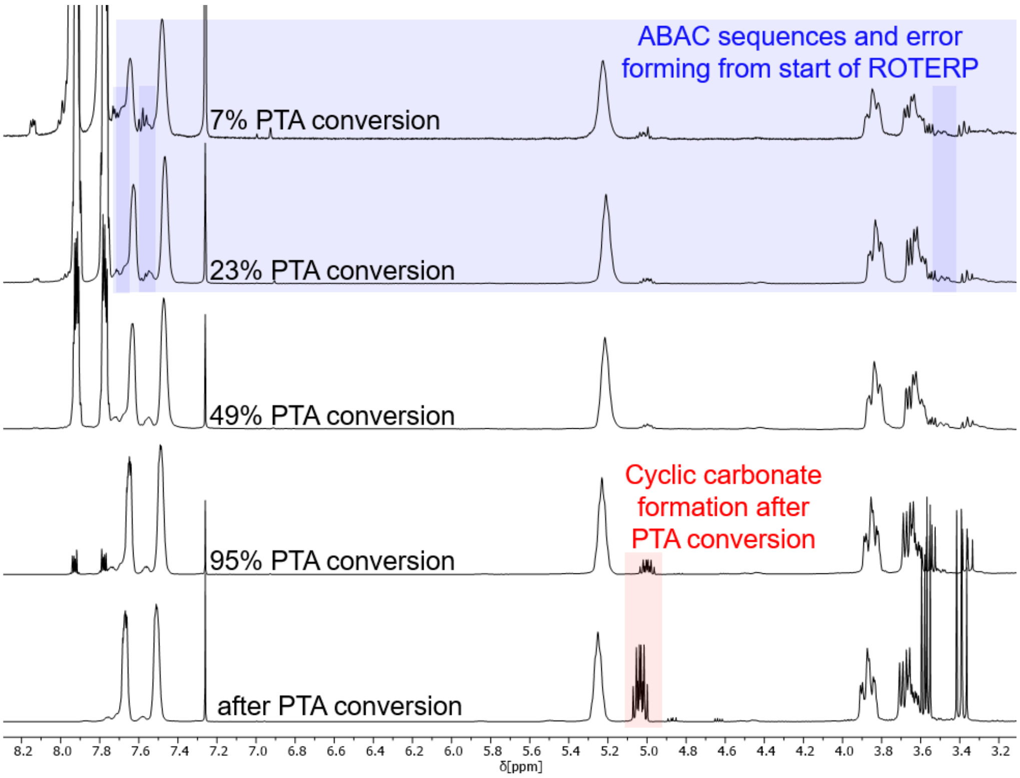

Figure S 23: ^{1}H NMR spectrum (400 MHz, $CDCl_3$, 25°C) of aliquots removed at different stages of ROTERP corresponding to table 1, run #9.

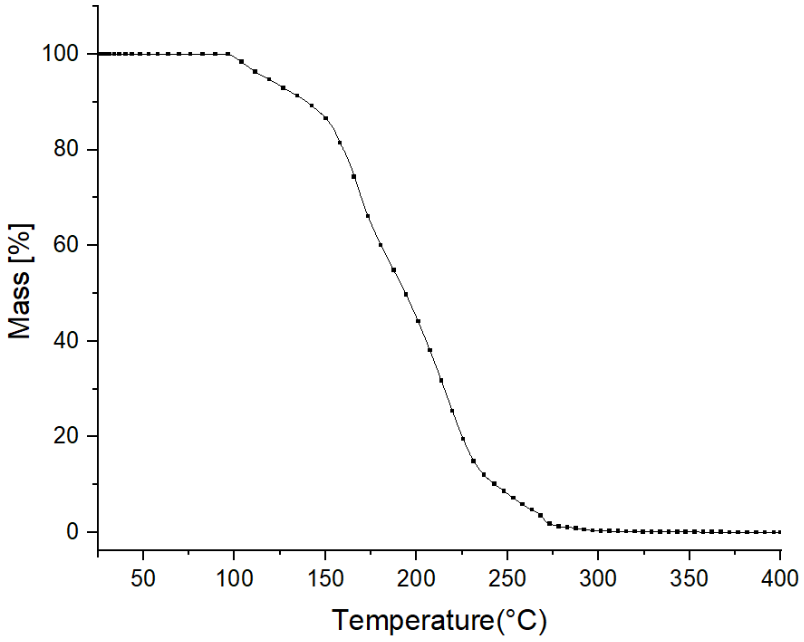

Figure S 24: TGA of CS_2/PO copolymer reported in this study obtained from the reaction of 1 LiHMDS: 500 PO : 1000 CS_2 at 25°C for 16h.

Section S4: SEC traces

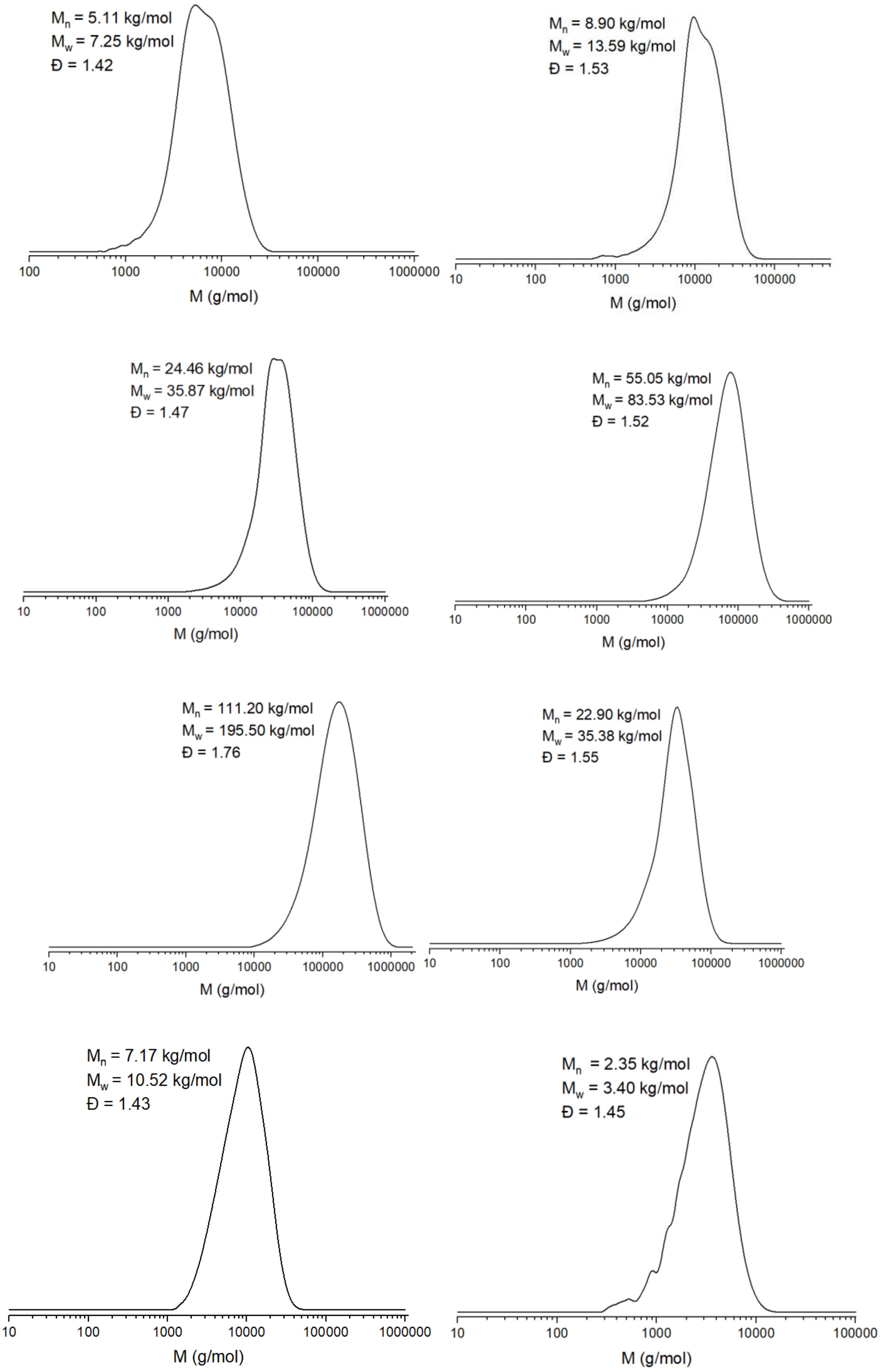

Figure S 25: SEC traces of polymers reported in this study.

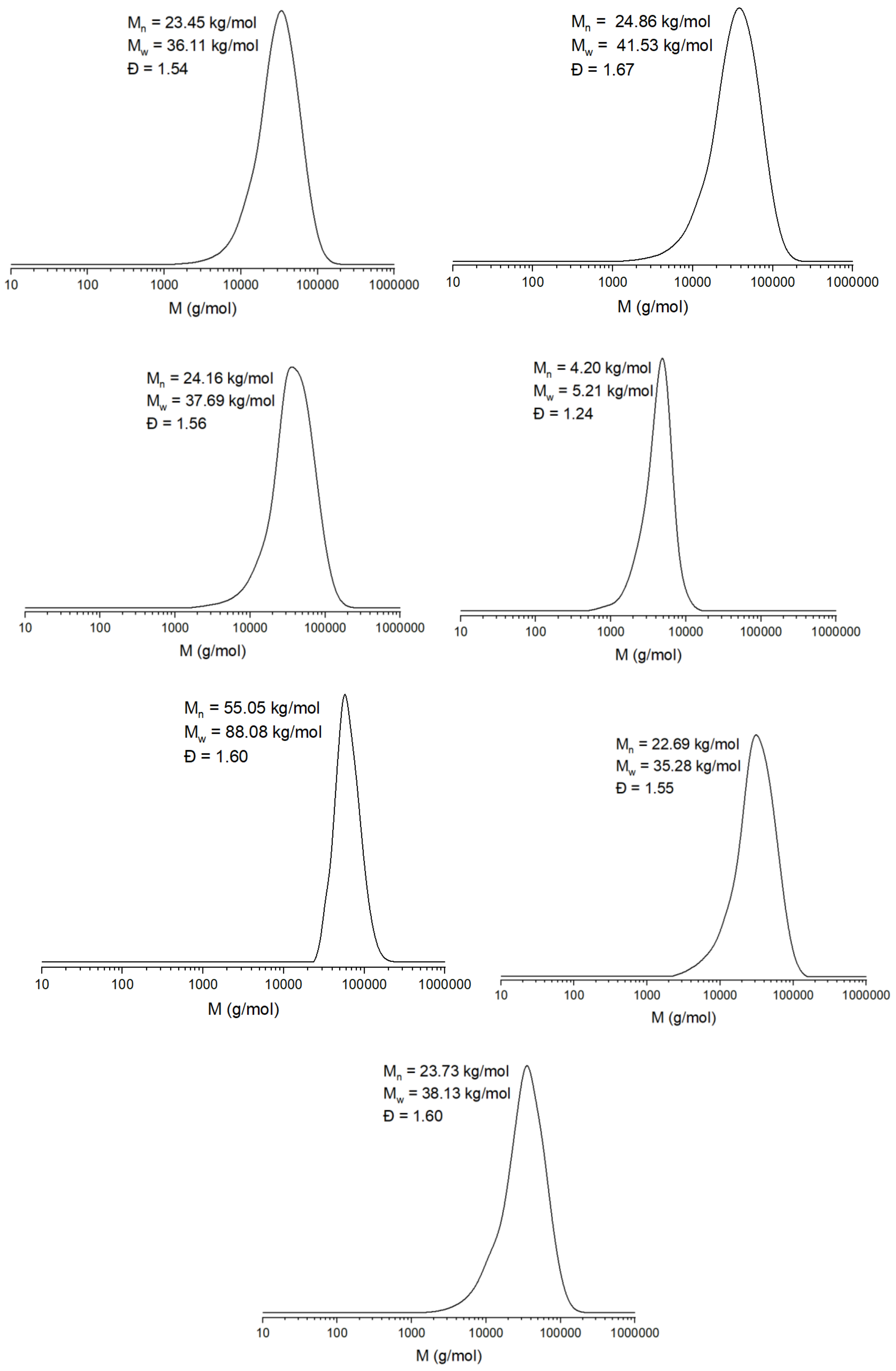

Figure S 26: SEC traces of polymers reported in this study.

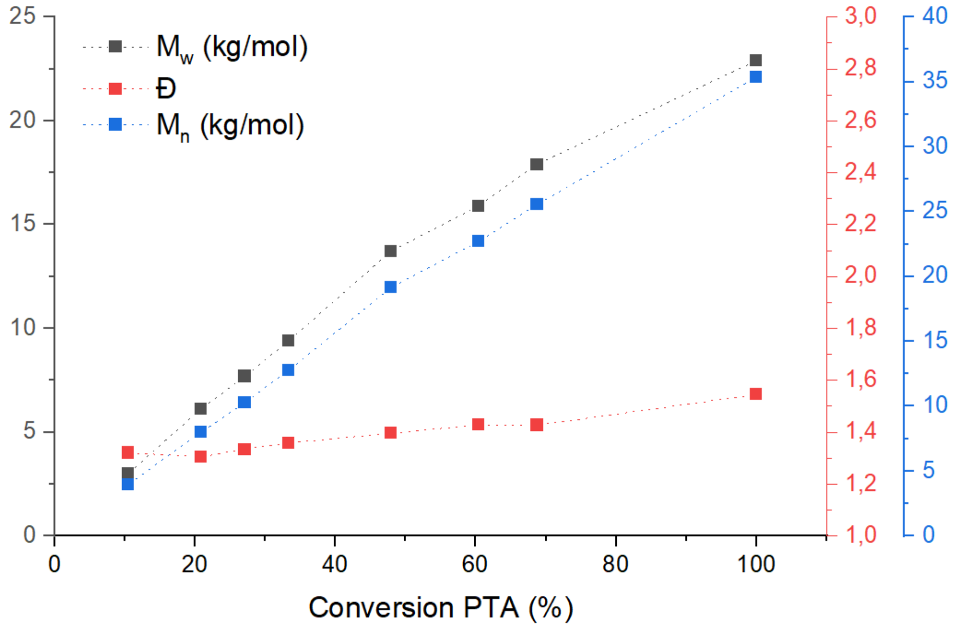

Figure S 27: Development of molecular masses and polydispersity of terpolymer corresponding to table 1, run #9.

Section S5: Synthesis, characterisation and reactivity of model MTC

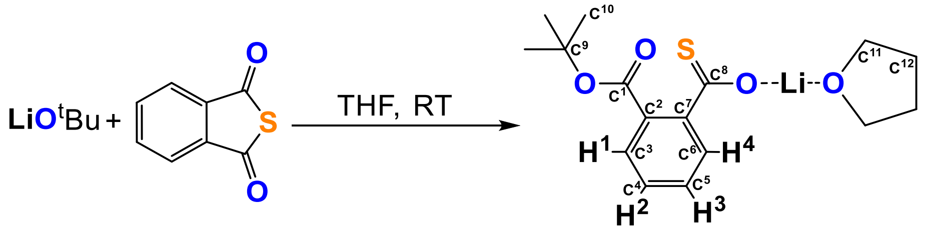

Synthesis of MTC·THF**:** Inside an Argon filled glovebox, phtalic thioanhydride (164.2 mg, 1.00 mmol) and LiO^{t}Bu (80.0 mg, 1.00 mmol) were loaded into an oven dried Schkenk fask, brought outside the glovebox and connected to an Schlenkline. Dry THF (25mL) was added and the mixture was stirred overnight giving an orange solution. All volatiles were removed in vacuum yielding **MTC**·THF as a yellow solid (300.0 mg, 0.95 mmol, 95%). XRD quality crystals were grown by layering a saturated THF solution with Pentane.

^{1}H NMR (400 MHz, d$_8$-THF, 25°C); δ(ppm): 7.77 (dd, *J* = 7.5, 1.1 Hz, 1H, H^4), 7.35 (dd, *J* = 7.5, 1.1 Hz, 1H, H^1), 7.25 (td, *J* = 7.5, 1.4 Hz, 1H, H^2), 7.16 (td, *J* = 7.5, 1.3 Hz, 1H, H^3), 3.65 – 3.53 (m, 4H, THF), 1.86 – 1.71 (m, 4H, THF), 1.52 (s, 6H, ^{t}Bu).

^{13}C{^{1}H} NMR (101 MHz, d$_8$-THF, 25°C); δ(ppm): 218.63 (C8), 169.33 (C1), 149.04 (C7), 131.31 (C2), 129.61 (C3), 129.47 (C5), 128.06 (C4), 127.24 (C6), 80.81 (C11), 68.02 (C9), 28.08 (C12), 26.13 (C10).

^{7}Li NMR (156 MHz, d$_8$-THF, 25°C); δ(ppm): 0.63.

FTIR [cm^{-1}]: 1685.1 (Ester C=O), 1450.8 (Thiocarboxylate C=O)

HRESI-MS (negative mode): m/z = calculated [M - Li]$^-$ 237.0585. Found 237.0650

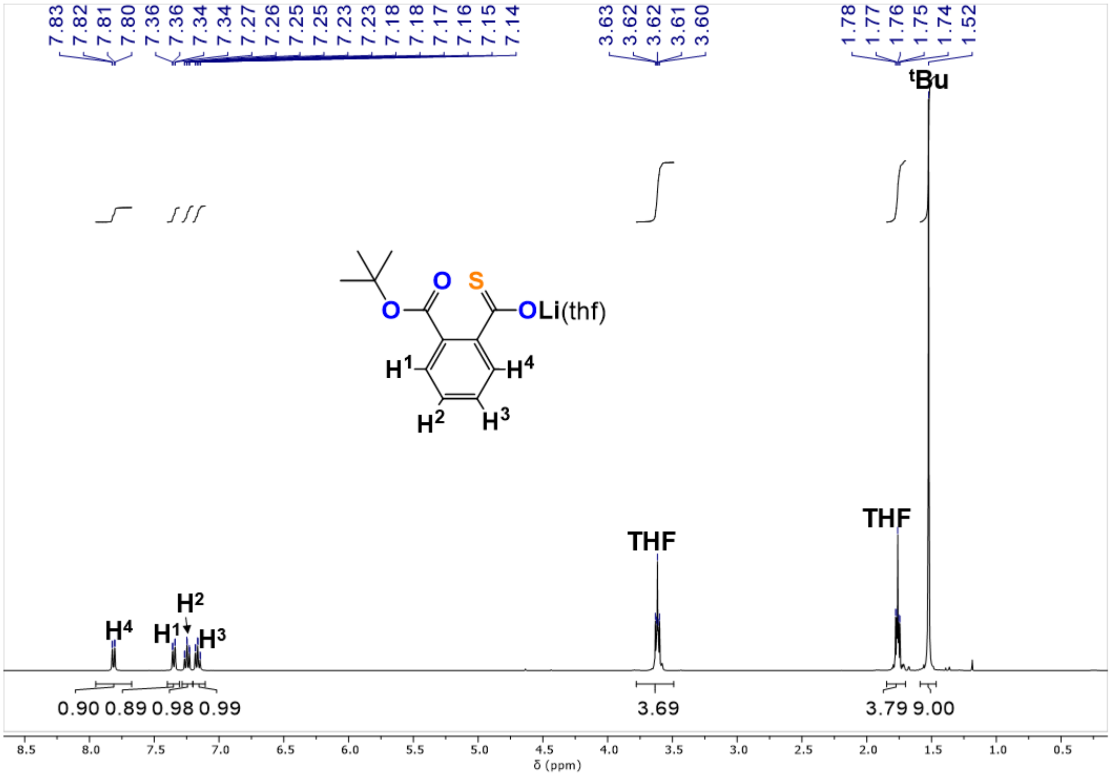

Figure S 28: ^{1}H NMR spectrum (400 MHz, d$_8$-THF, 25°C) of **MTC**·THF.

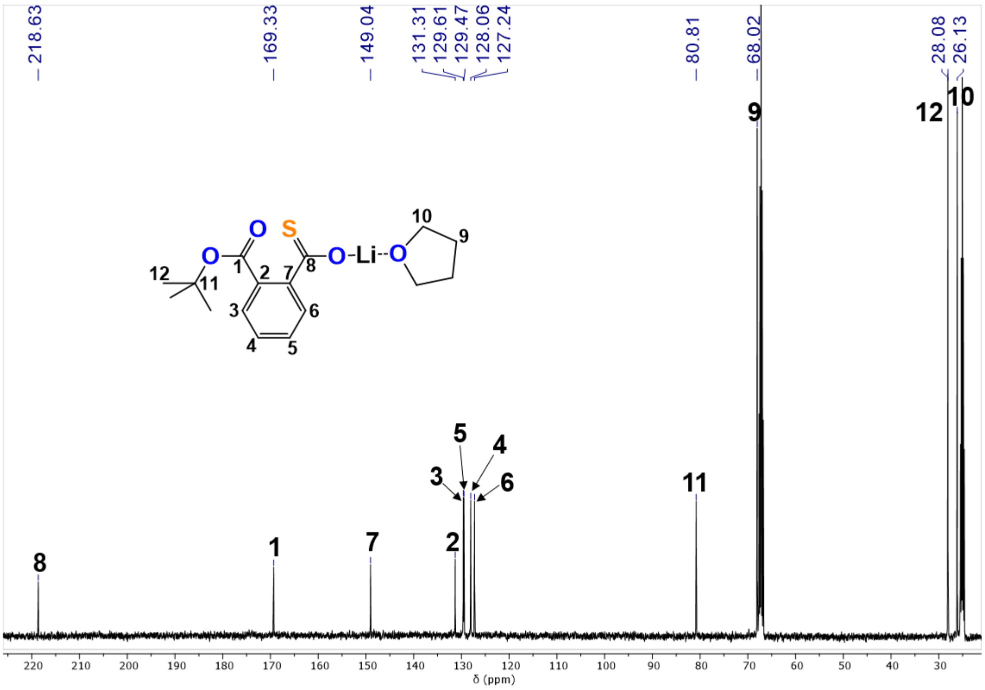

Figure S 29: ^{13}C{^{1}H} NMR spectrum (101 MHz, d$_8$-THF, 25°C) of **MTC**.

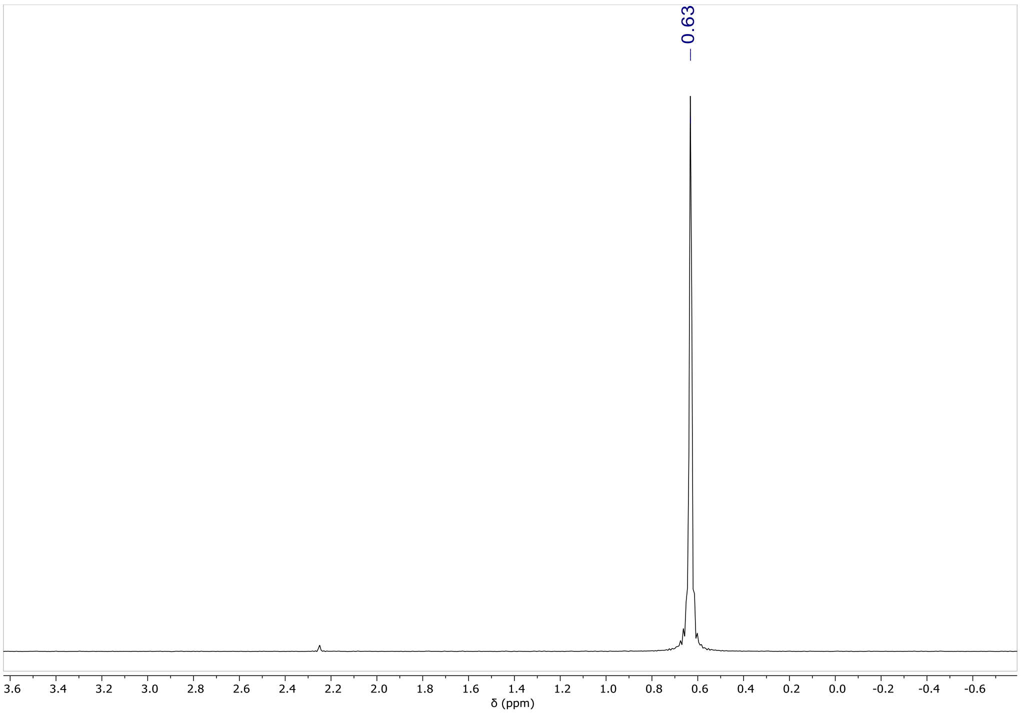

Figure S 30: ^{7}Li NMR spectrum (194 MHz, d_8-THF, 25°C) of **MTC**. Signal at 2.25 ppm corresponds to internal $LiSO_3CF_3$ standard.

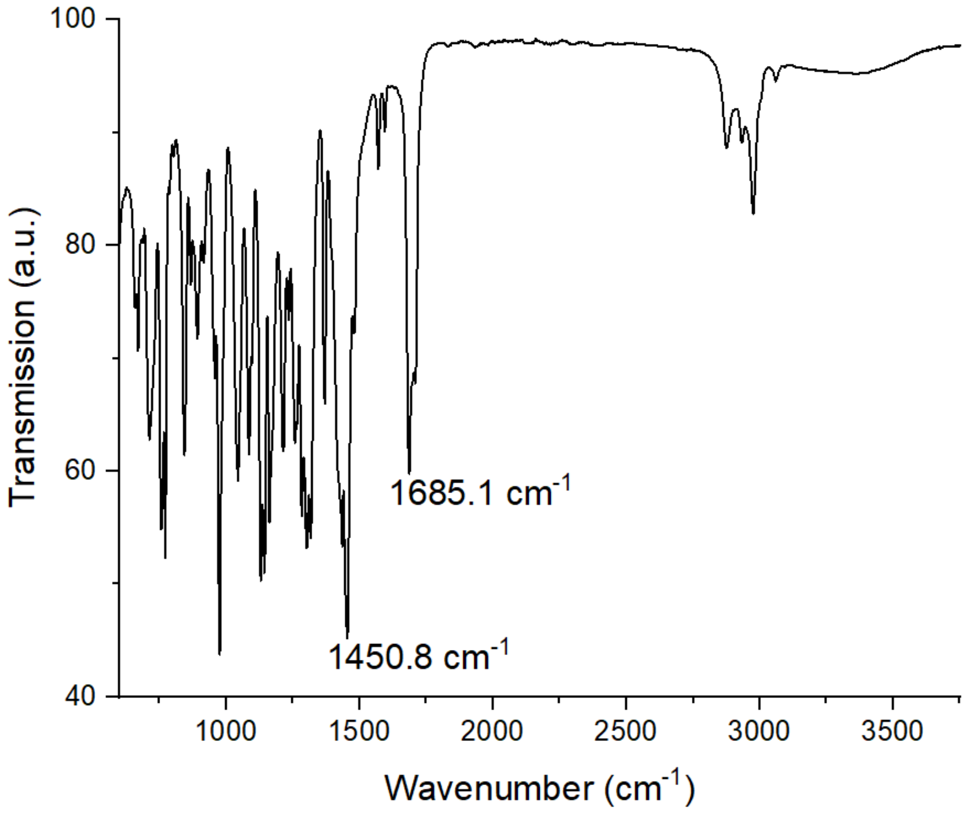

Figure S 31: Solid state FT-ATIR spectrum of **MTC**.

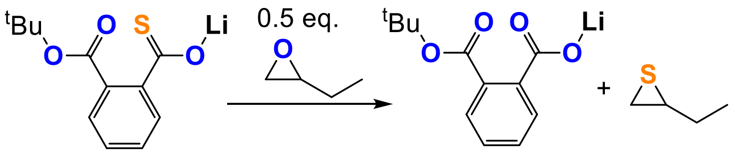

In an oven dried J. Youngs NMR Tube **MTC·**THF (50.0 mg, 0.16 mmol, 1 eq.) dissolved in d_8-THF (0.6 mL) and BO (7.0 µL, 0.08 mmol, 0.5 equiv.) was let react for 10 min and analysed by NMR. The mixture was then let react for another 2h and analysed by NMR. Longer reaction times or more equivalents BO result in the appearance of broad signal under disappearance of the thiiran signals presumably due to competitive formation of polythioether.

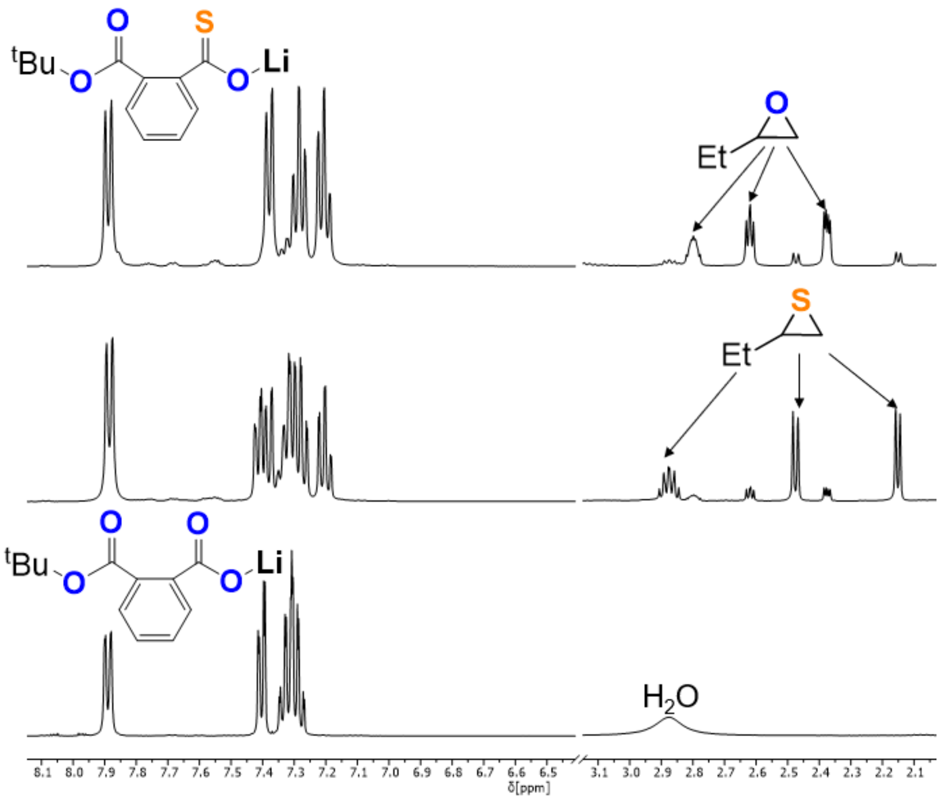

Figure S 32: Overlayed ^{1}H NMR (400 MHz, d_8-THF, 25°C) spectra of BO addition to **MTC** (top) 10 min after addition and (middle) 2h after addition and (bottom) carboxylate derivative of **MTC** prepared from 1 eq. $LiO^{t}Bu$ with phtalic anhydride.

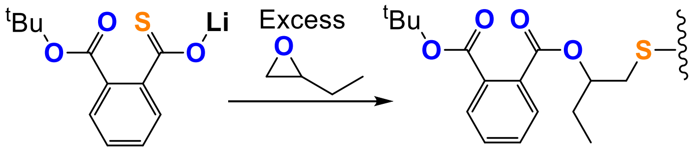

BO insertion from MTC: Excess butylene oxide (1mL) was added to solid **MTC**·THF (50mg). The resulting mixture was stirred for 30 min at room temperature before all volatiles were removed in vacuum prior to spectroscopic analysis.

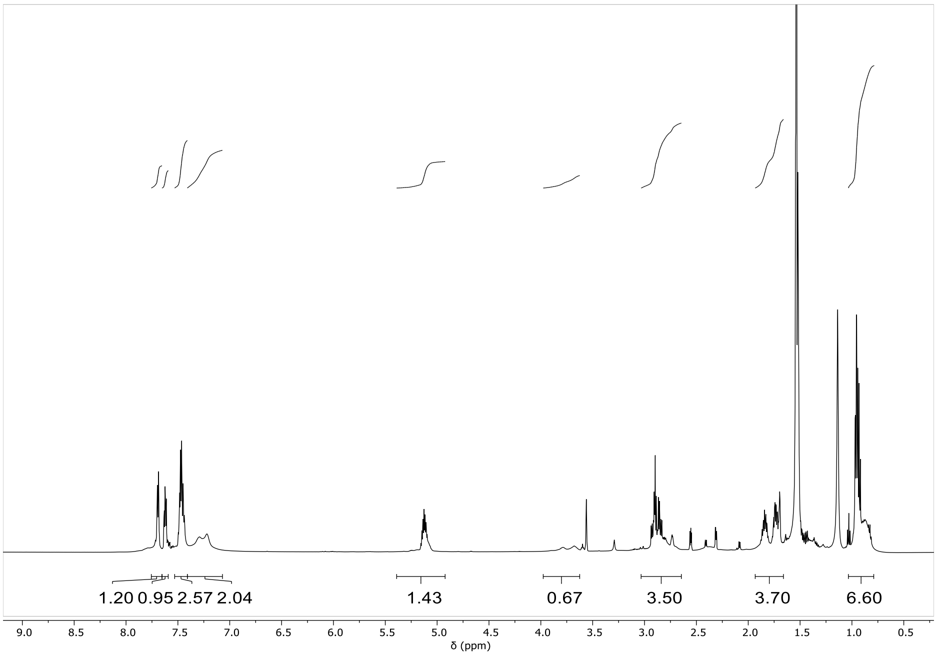

Figure S 33: ^{1}H NMR spectrum (400 MHz, d_8-THF, 25°C) of BO insertion products of **MTC**.

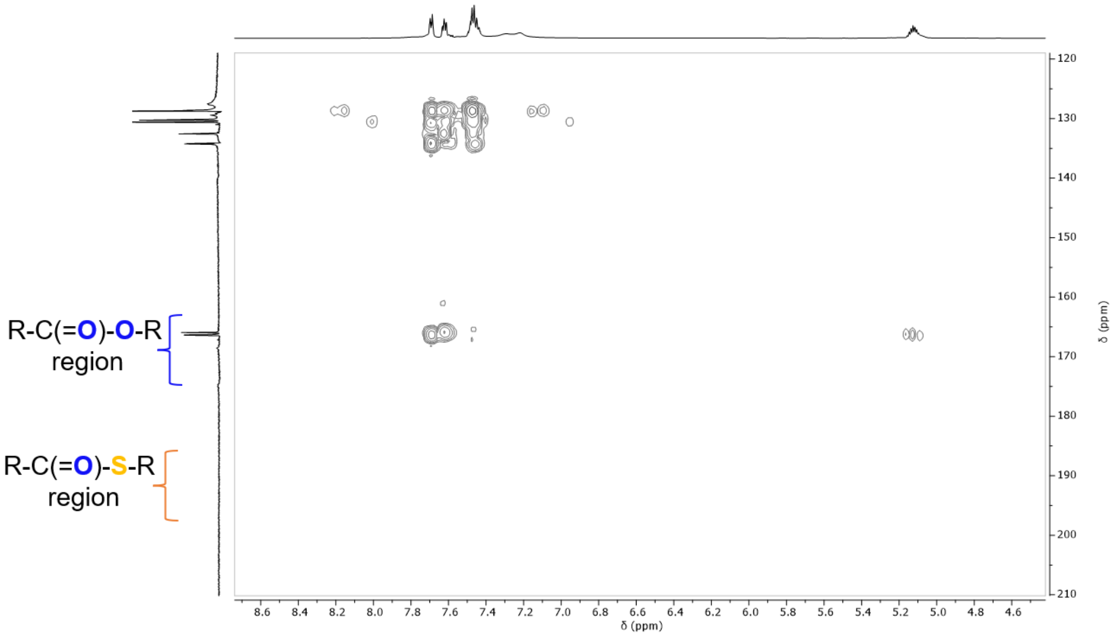

Figure S 34: Zoom into the carbonyl region of the ^{1}H-^{13}C HMBC NMR spectrum (d_8-THF, 25°C) of BO insertion of **MTC**.

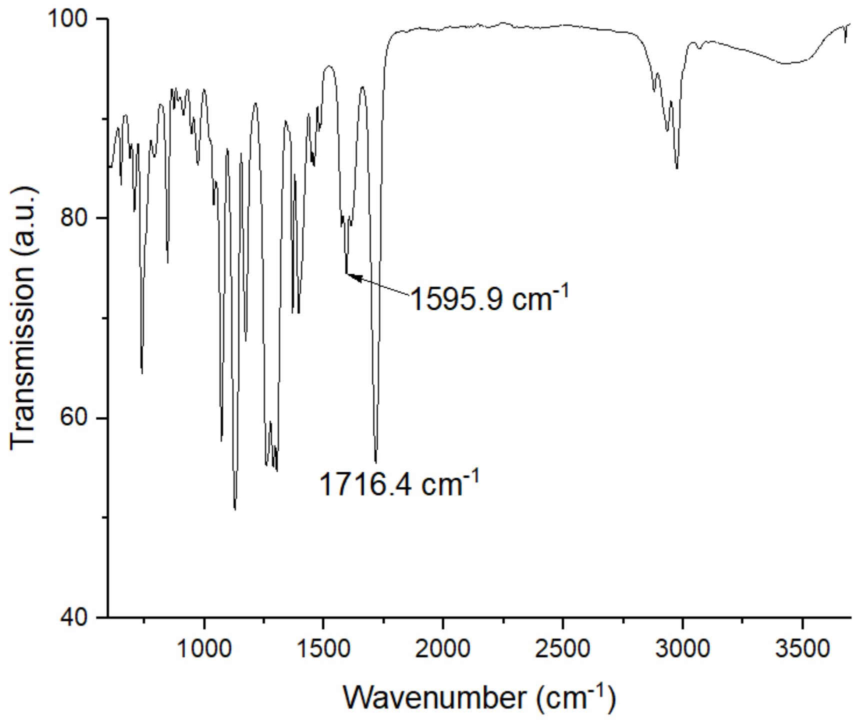

Figure S 35: Solid state FTIR spectrum of BO insertion of **MTC**.

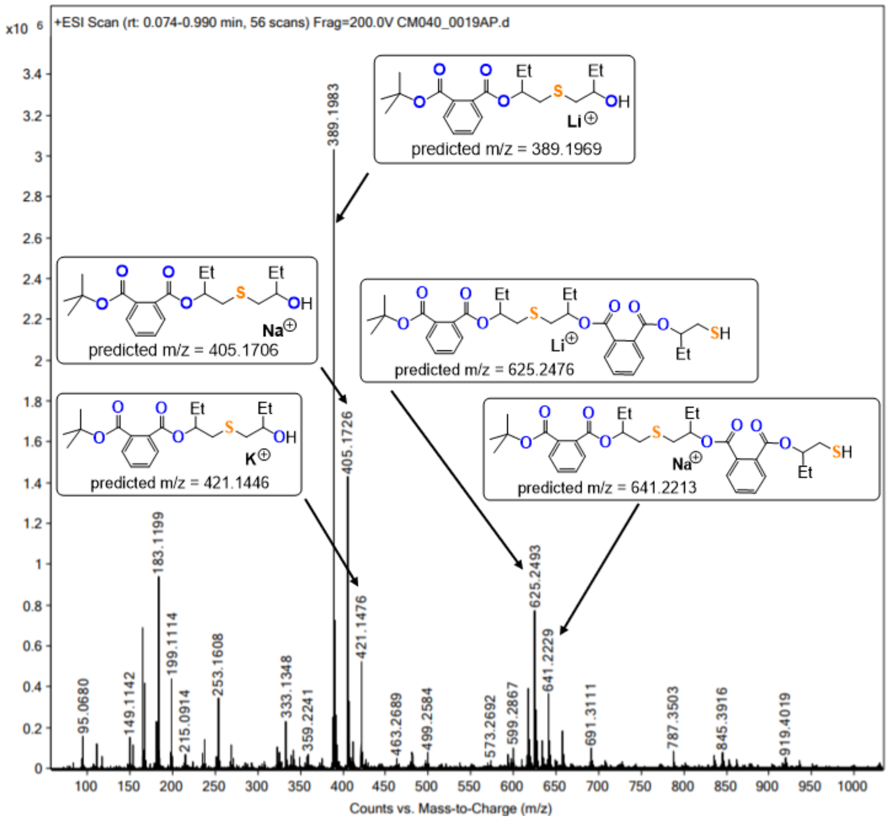

Figure S 36: Positive Mode HR-ESI MS of BO insertion products of **MTC**.

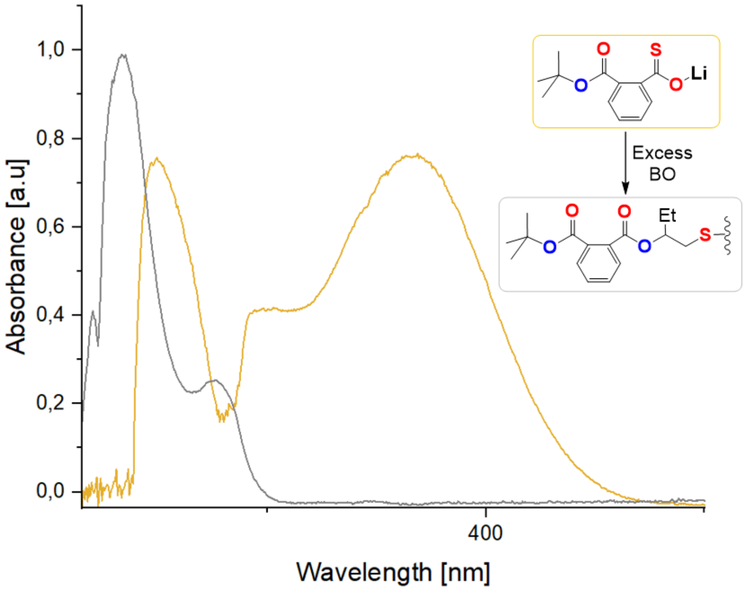

Figure S 37: UVVIS spectra before and after BO insertion of **MTC** in THF.

Reaction between MTC and BO in presence of CS_2: In an oven dried J. Youngs NMR Tube **MTC·**THF (50.0 mg, 0.16 mmol, 1 eq.) dissolved in d_8-THF (0.4 mL) and CS_2 (0.4 mL, 6.6 mmol, 42 equiv.) and BO (7.0 µL, 0.08 mmol, 0.5 equiv.) was added. The mixture was let react for 5 min and analysed by NMR.

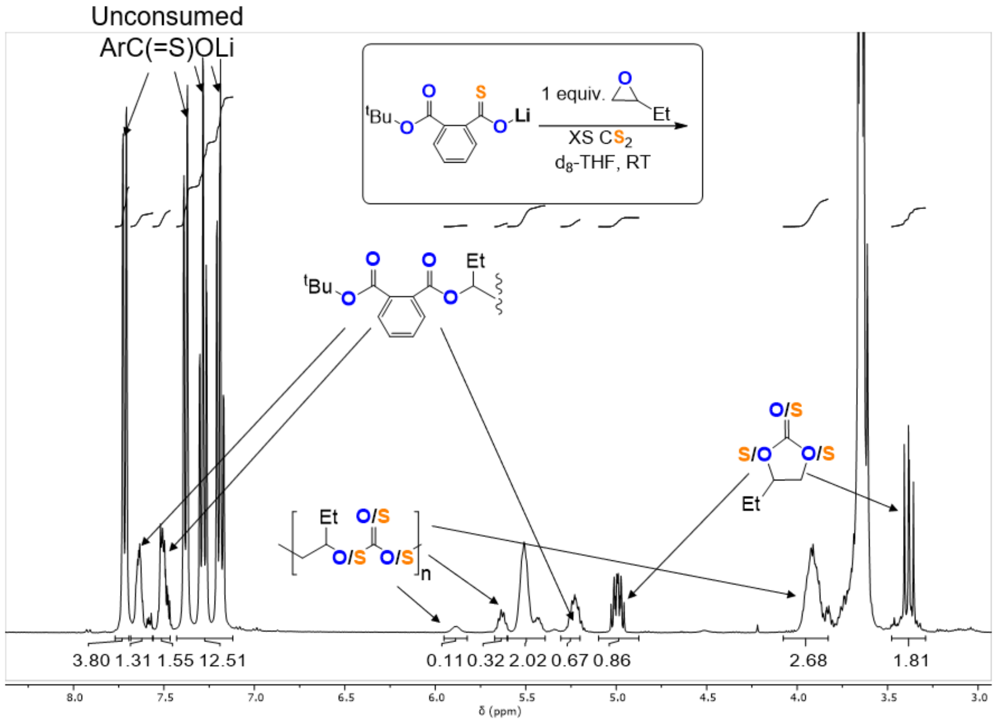

Figure S 38: ^{1}H NMR spectrum (400 MHz, d_8-THF, 25°C) of product mixture produced from the reaction of **MTC** with 1 eq. BO in presence of excess CS_2.

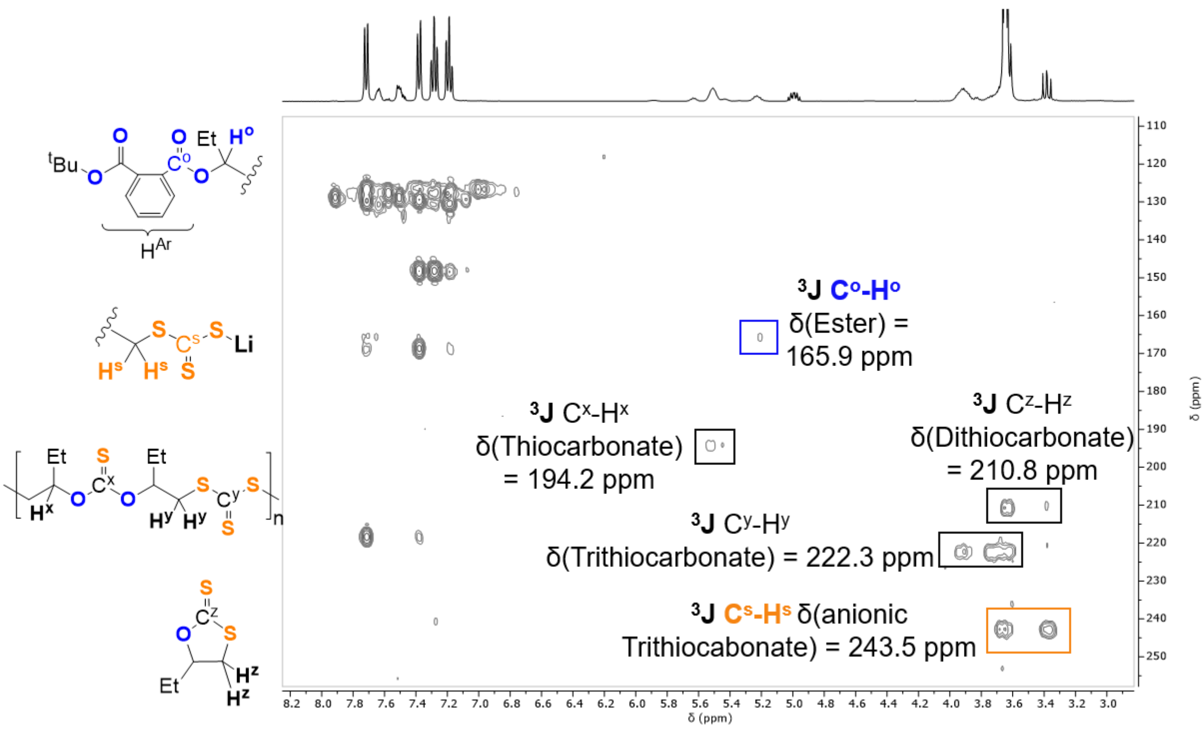

Figure S 39: ^{1}H-^{13}C HMBC NMR spectrum (d_8-THF, 25°C) of product mixture produced from the reaction of **MTC** with 1 eq. BO in presence of excess CS_2.

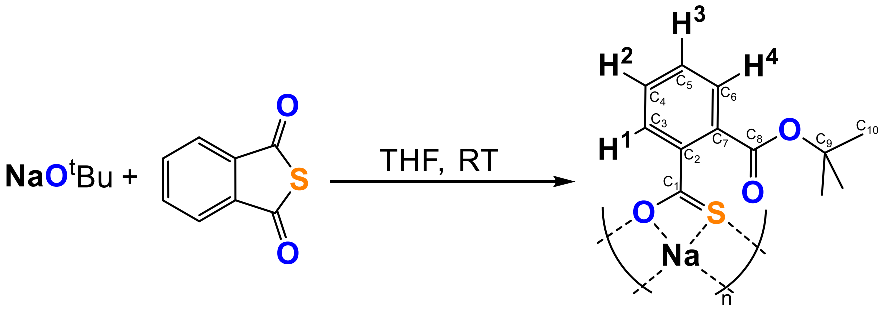

Synthesis of MTC^{Na}·0.75THF: Inside an Argon filled glovebox, phtalic thioanhydride (164.2 mg, 1.00 mmol) and NaO^{t}Bu (96.0 mg, 1.00 mmol) were loaded into an oven-dried Schlenk flask, brought outside the glovebox and connected to an Schlenkline. Dry THF (25mL) was added, and the mixture was stirred overnight giving an orange suspension. All volatiles were removed in vacuum yielding **MTC^{Na}** as a orange solid (298.3 mg, 0.95 mmol, 95%). XRD quality crystals were grown by layering the crude reaction mixture with pentane.

^{1}H NMR (400 MHz, d_8-THF, 25°C); δ(ppm): 7.83 (d, J = 7.5 Hz, 1H, H-1), 7.35 – 7.14 (m, 4H, H-2/H-3/H-4), 3.82 – 3.37 (m, THF), 2.01 – 1.61 (m, THF), 1.48 (s, 9H, ^{t}Bu).

Due to the insolubility of **MTC^{Na}**, ^{13}C data was obtained via ^{1}H-^{13}C HMBC NMR spectroscopy.

$^{13}C\{^{1}H\}$ NMR (101 MHz, d_8-THF, 25°C); δ(ppm): 209.92 (C1), 169.52 (C8), 148.29 (C2), 131.29 (C7), 129.45 (C6), 129.00 (C4). 127.41 (C5), 127.00 (C2), 80.28 (C9), 28.34 (C10)

FTIR [cm^{-1}]: 1684.0 (Ester C=O), 1451.6 (Thiocarboxylate C=O)

HRESI-MS (negative mode): m/z = calculated $[M - Na]^-$ 237.0585. Found 237.0545

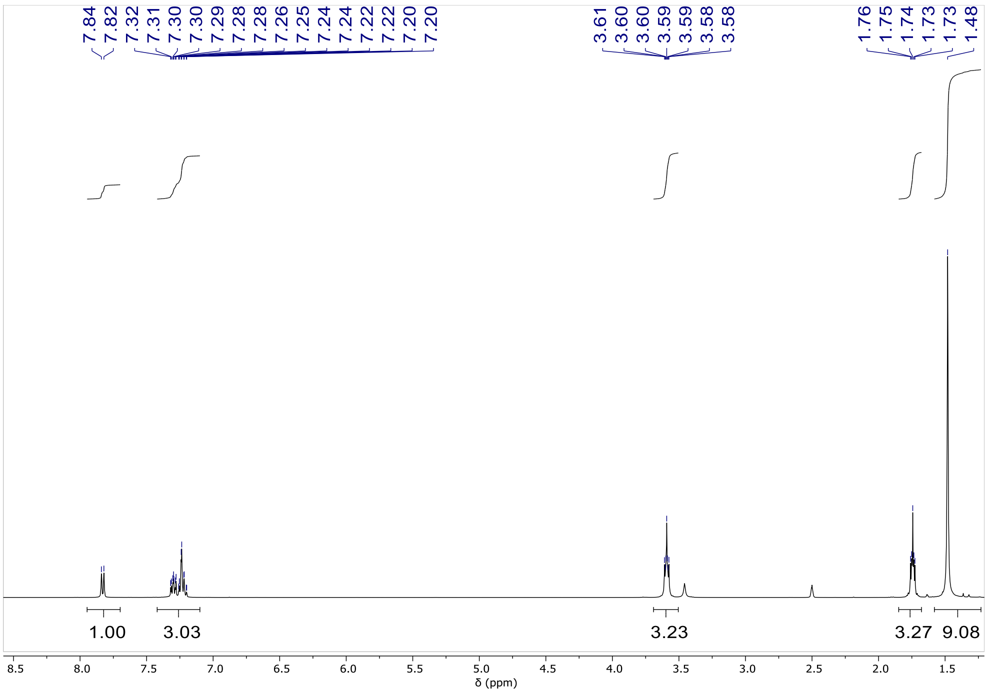

Figure S 40: ^{1}H NMR spectrum (400 MHz, d_6-DMSO, 25°C) of **MTC^{Na}**·0.75THF.

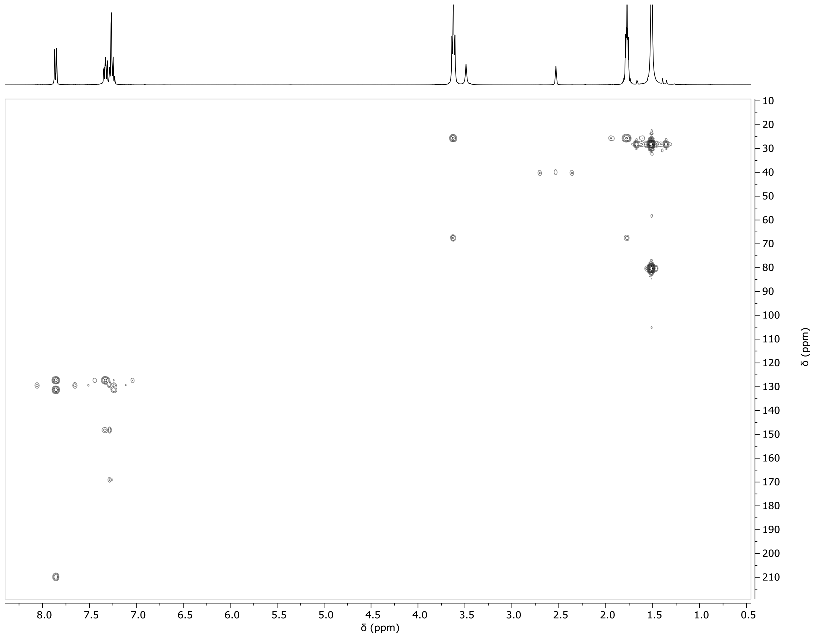

Figure S 41: ^{1}H - ^{13}C HMBC NMR spectrum (126 MHz, $CDCl_3$, 25°C) of **MTC^{Na}**·0.75THF.

Reaction between MTC^{Na} and BO in presence of CS_2: In an oven dried J. Youngs NMR Tube **MTC·**0.75THF (50.3 mg, 0.16 mmol, 1 eq.) dissolved in d_8-THF (0.4 mL) and CS_2 (0.4 mL, 6.6 mmol, 42 equiv.) and BO (7.0 µL, 0.08 mmol, 0.5 equiv.) was added. The mixture was let react for 16 h and analysed by NMR.

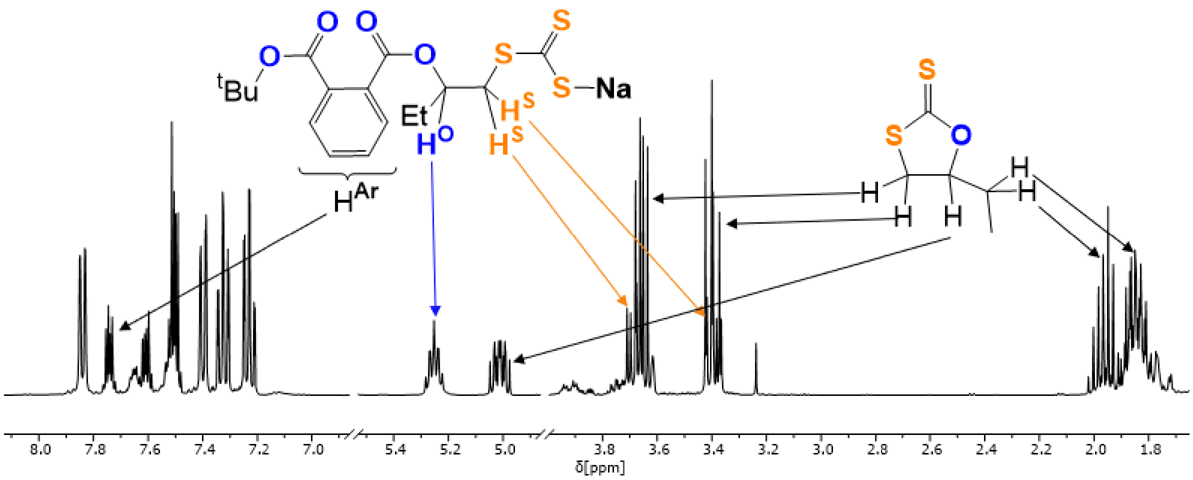

Figure S 42: ^{1}H NMR spectrum (400 MHz, d_8-THF, 25°C) of product mixture product from the stochiometric reaction of

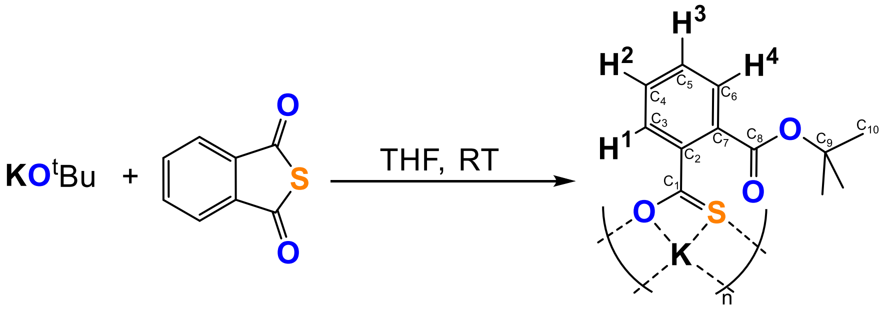

Synthesis of MTC^K: Inside an Argon filled glovebox, phtalic thioanhydride (164.2 mg, 1.00 mmol) and KO^{t}Bu (112.2 mg, 1.00 mmol) were loaded into an oven-dried Schkenk fask, brought outside the glovebox and connected to an Schlenkline. Dry THF (25mL) was added and the mixture was stirred overnight giving an orange suspension. All volatiles were removed in vacuum yielding **MTC^K** as a orange solid (262.6 mg, 0.95 mmol, 95%). XRD quality crystals were grown by layering a crude reaction mixture with Pentane.

^{1}H NMR (400 MHz, d_8-THF, 25°C); δ(ppm): δ 7.79 (ddd, J = 7.7, 1.2, 0.6 Hz, 1H, H^1), 7.27 (ddd, J = 7.7, 6.4, 2.3 Hz, 1H, H^2), 7.23 – 7.15 (m, 2H, H^{3}/^{4}), 1.46 (s, 9H, ^{t}Bu).

Due to the insolubility of **MTC^K**, ^{13}C data was obtained via ^{1}H-^{13}C HMBC NMR spectroscopy.

^{13}C{^{1}H} NMR (101 MHz, d$_8$-THF, 25°C); δ(ppm): 208.10 (C1), 169.31 (C8), 148.84 (C2), 131.10 (C7), 129.24 (C6), 129.00 (C4). 127.23 (C5), 126.96 (C2), 81.50 (C9), 28.90 (C10)

FTIR [cm^{-1}]: 1684.5 (Ester C=O), 1451.9 (Thiocarboxylate C=O)

HRESI-MS (negative mode): m/z = calculated [M - K]$^-$ 237.0585. Found 237.0572

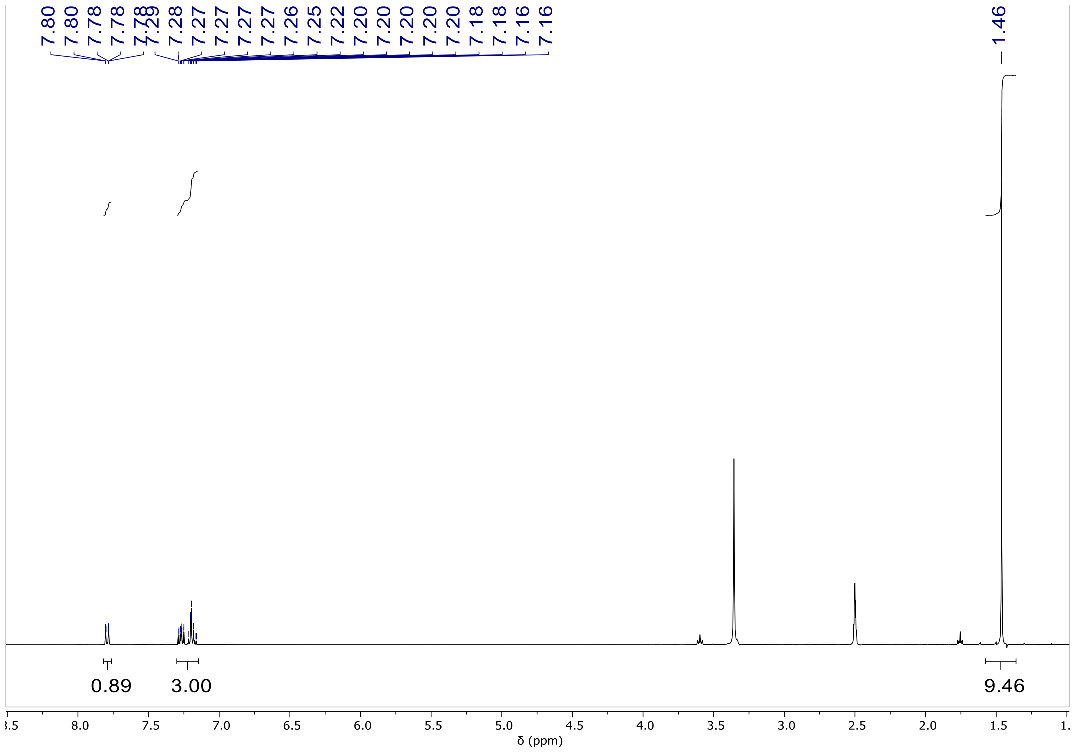

Figure S 43: ^{1}H NMR spectrum (400 MHz, d$_6$-DMSO, 25°C) of **MTC^{K}**.

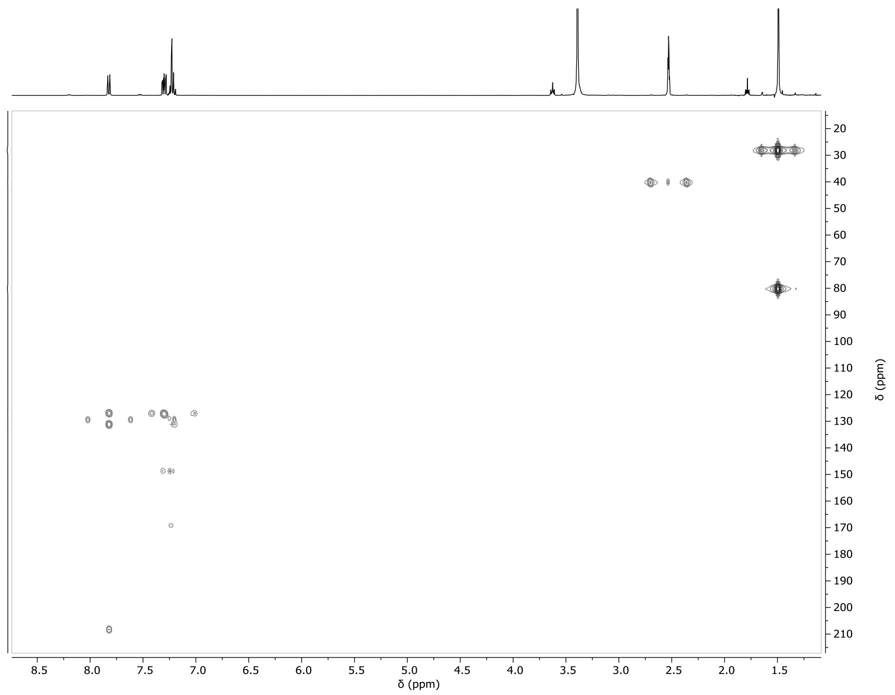

Figure S 44: ^{1}H - ^{13}C HMBC NMR spectrum ($CDCl_3$, 25°C) of $\mathbf{MTC^{K}}$.

Section S6: Switchable Catalysis.

εDL ROP protocol: In an argon-filled glovebox, LiHMDS (6.4 mg, 38.5 µmol, 1 equiv.) was dissolved in butylene oxide (1670.0 µl, 1386.9 mg, 19.2 mmol, 500 equiv.) and benzylalcohol (4.0 µl, 4.2 mg, 36.5 µmol, 1 equiv.) was added. The resulting mixture was stirred for 1 min and afterwards ε-decalactone (εDL) (630.6 µl, 655.5 mg, 3.9 mmol, 100 equiv.) was added. Aliquots were removed at regular time intervals and quenched by addition into $CDCl_3$ containing benzoic acid (10 mg/ml) which were then analysed by ^{1}H NMR and SEC.

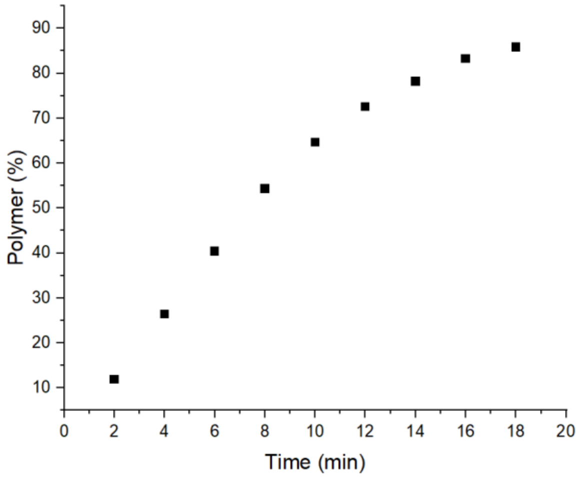

Figure S 45: εDL conversion to PDL polymer versus time plot.

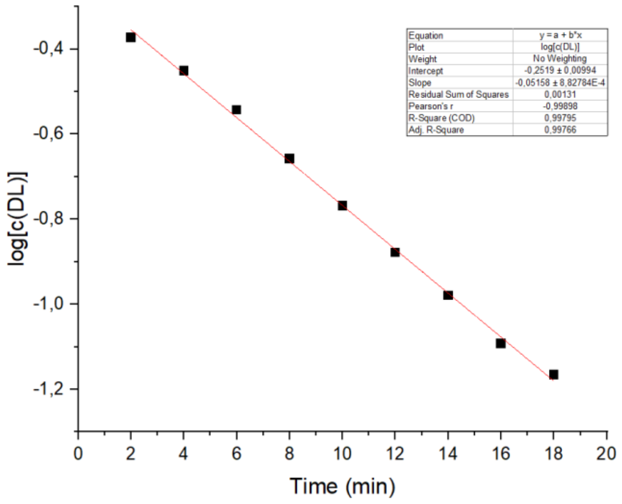

Figure S 46: Logarhitmic εDL conversion to PDL polymer versus time plot.

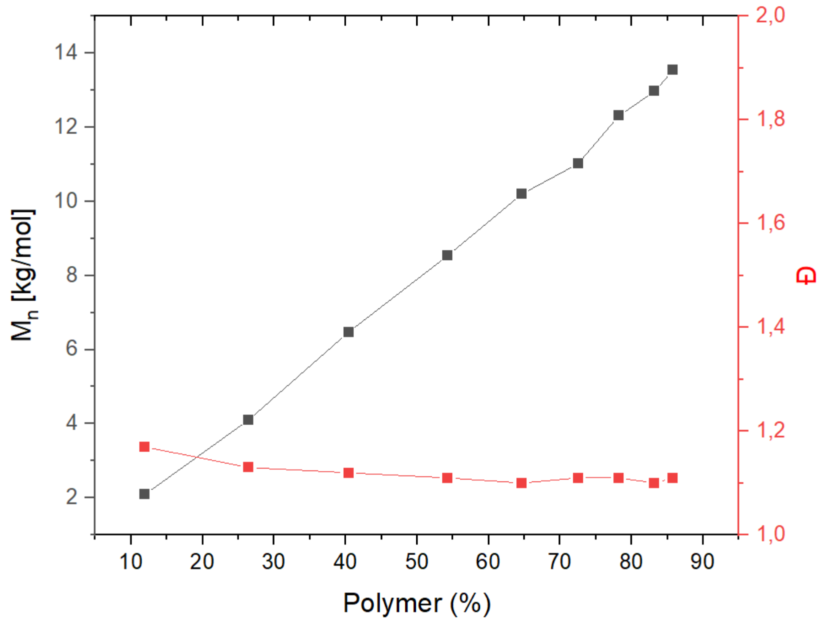

Figure S 47: Plot showing the development of M_n and polydispersity of PDL with respect to εDL conversion.

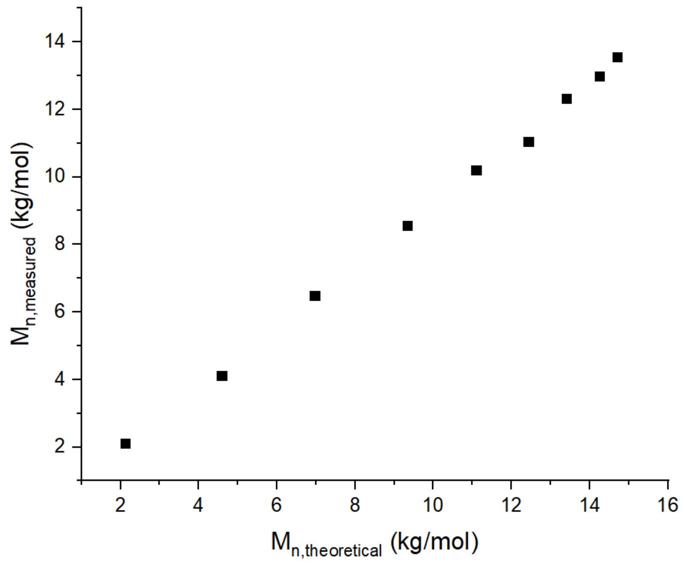

Figure S 48: Correlation of measured and theoretical M_n of PDL.

εDL ROP into PTA/CS₂/BO ROTERP switch protocol: In an argon-filled glovebox, LiHMDS (6.4 mg, 38.5 µmol, 1 equiv.) was dissolved in butylene oxide (835.0 µl, 693.5 mg, 9.6 mmol, 250 equiv.) and benzylalcohol (4.0 µl, 4.2 mg, 36.5 µmol, 1 equiv.) was added. The resulting mixture was stirred for 1 min and afterwards ε-decalactone (εDL) (335.3 µl, 327.3 mg, 19.25 mmol, 50 equiv.) was added and ROP was allowed to occur for 15 min. Afterwards CS_2 (1083.0 µl, 1364.6 mg, 19.3 mmol) and PTA (442.4 mg, 27.0 mmol, 70 equiv.) were added causing an immediate yellow discolouration of the mixture which was then allowed to react for 30 min at 80°C. The polymer was isolated by two consecutive precipitations from DCM/MeOH, followed by isolation by filtration and drying in a vacuum oven set to 60°C overnight.

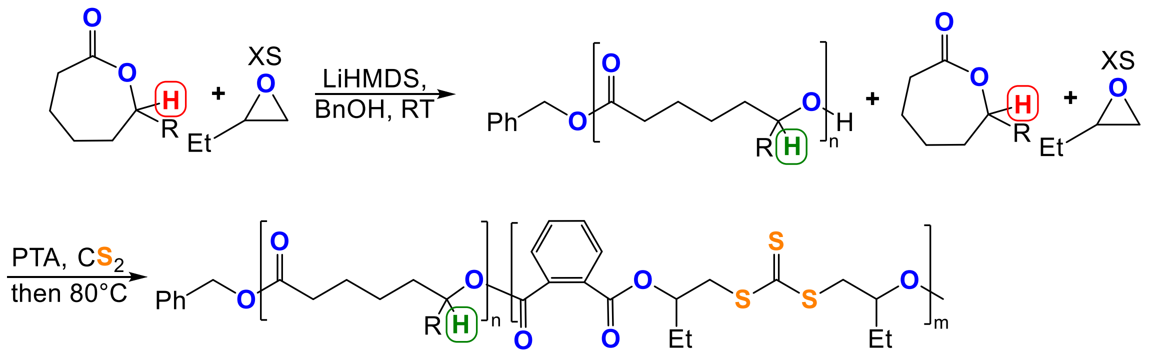

Figure S 49: Schematic showing εDL ROP to PTA/CS_2/BO ROTERP switchable catalysis.

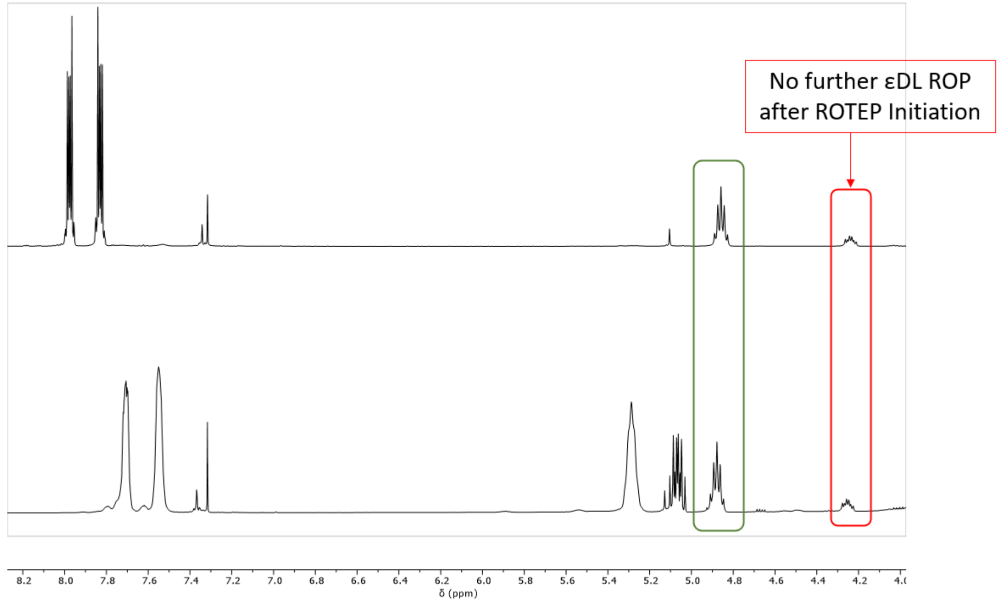

Figure S 50a: ^{1}H NMR spectra (400 MHz, $CDCl_3$, 25°C) of aliquots removed before and after ROTERP phase of switchable catalysis showing that no further ROP occurs after ROTERP initiation.

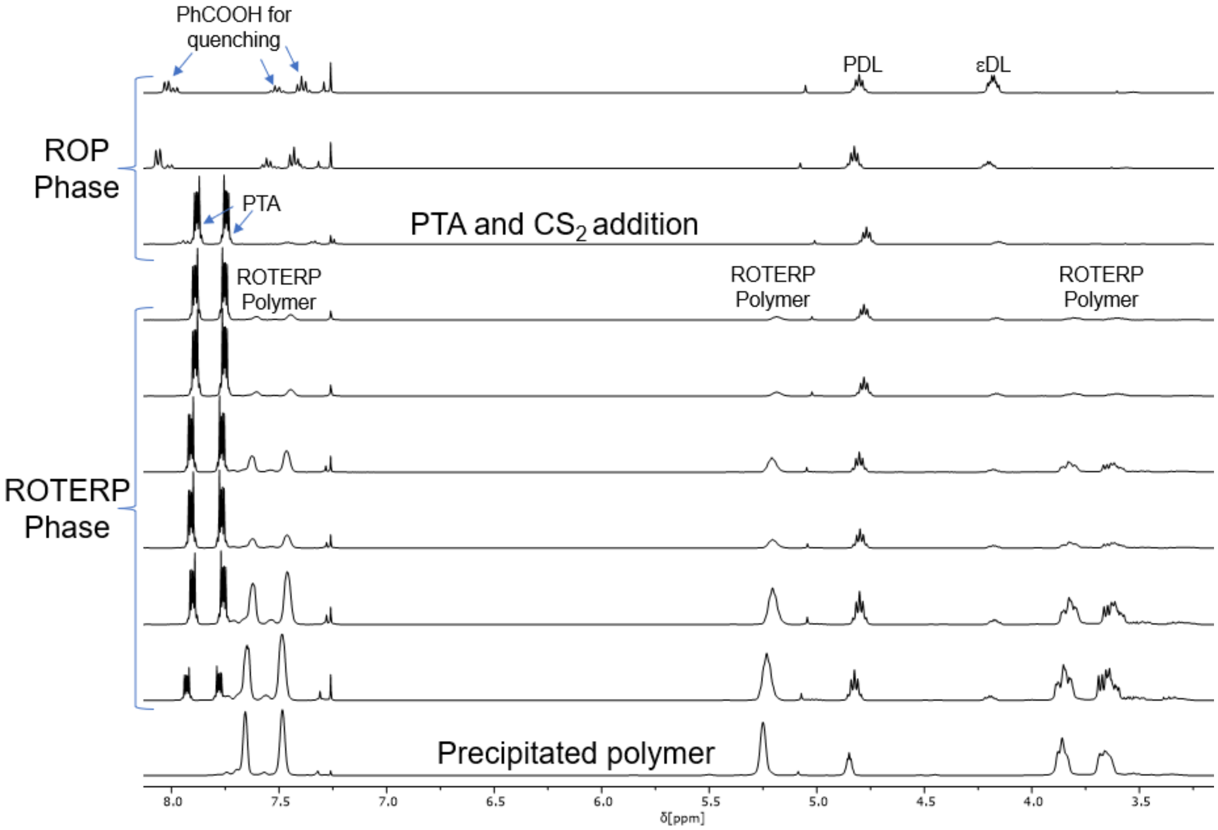

Figure S 51b: ^{1}H NMR spectra (400 MHz, $CDCl_3$, 25°C) of aliquots removed throughout ROP and ROTERP phase of switchable catalysis.

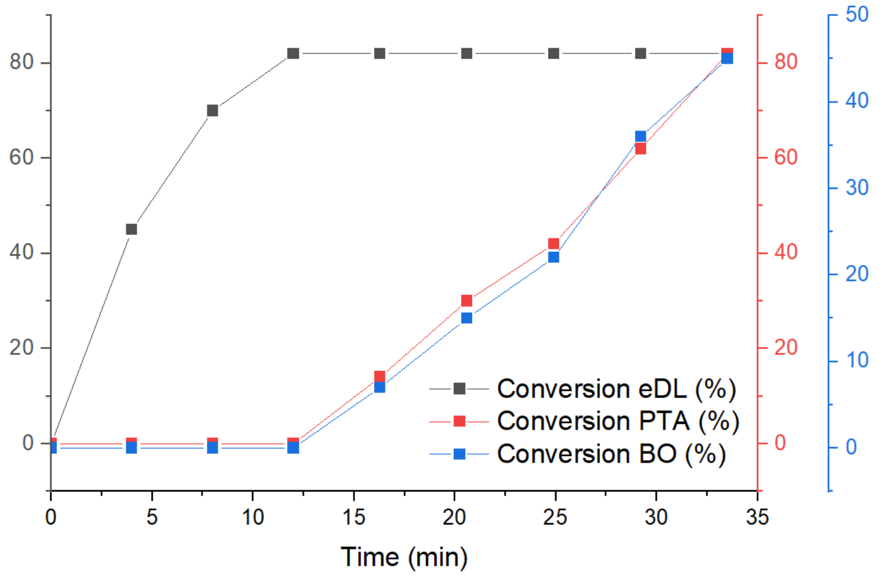

Figure S 52c: Conversion versus time plot (by integration of the ^{1}H NMR spectra) of switchable catalysis corresponding to Figure S 50b. Note that aliquot removal in the ROP phase results in the final BO conversion deviating from theoretical 40%.

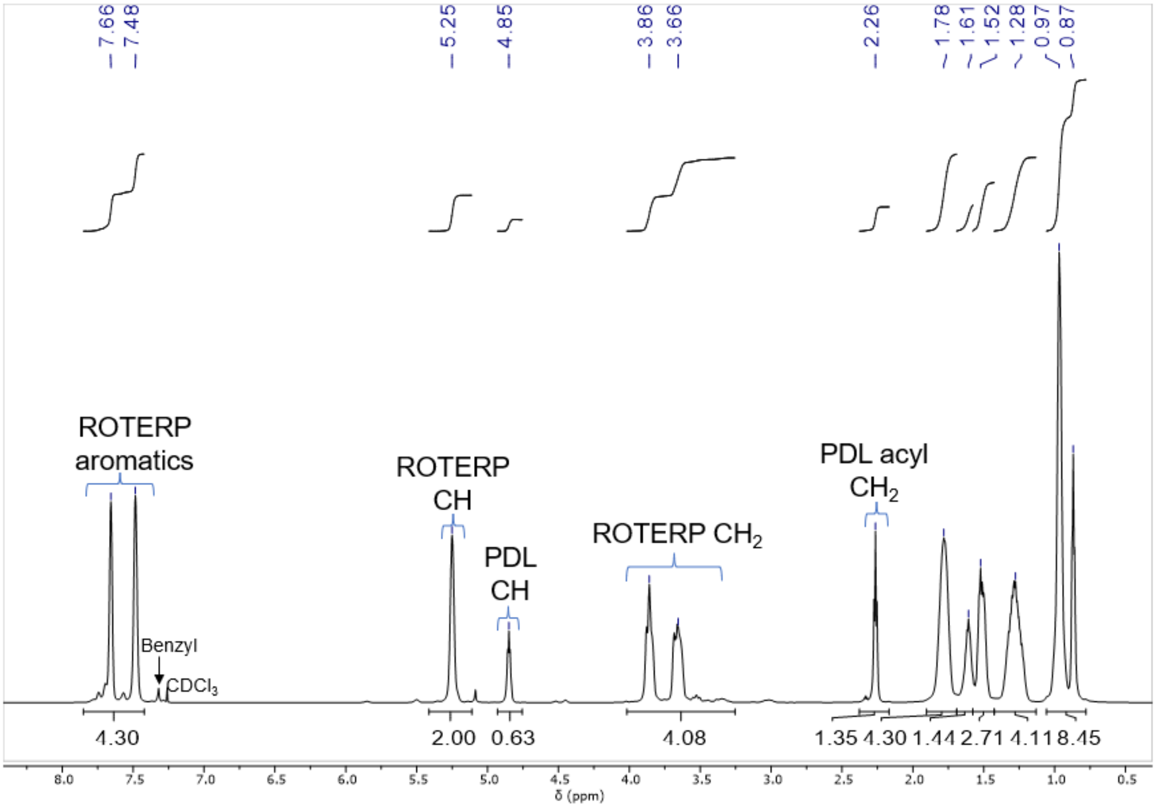

Figure S 53: ^{1}H NMR spectrum (400 MHz, $CDCl_3$, 25°C) of εDL ROP to PTA/CS_2/BO ROTERP polymer. CH_x labels refer to protons adjacent to heteroatoms.

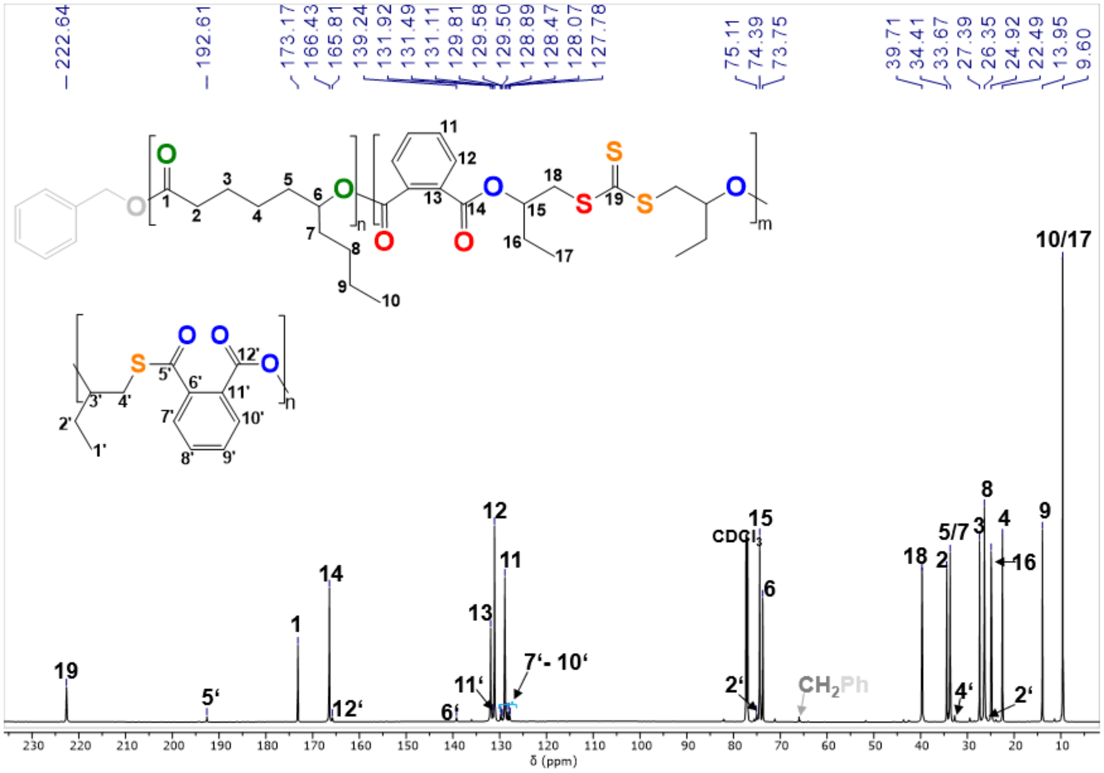

Figure S 54: $^{13}C\{^1H\}$ NMR spectrum (126 MHz, $CDCl_3$, 25°C) of εDL ROP to PTA/ CS_2/BO ROTERP polymer.

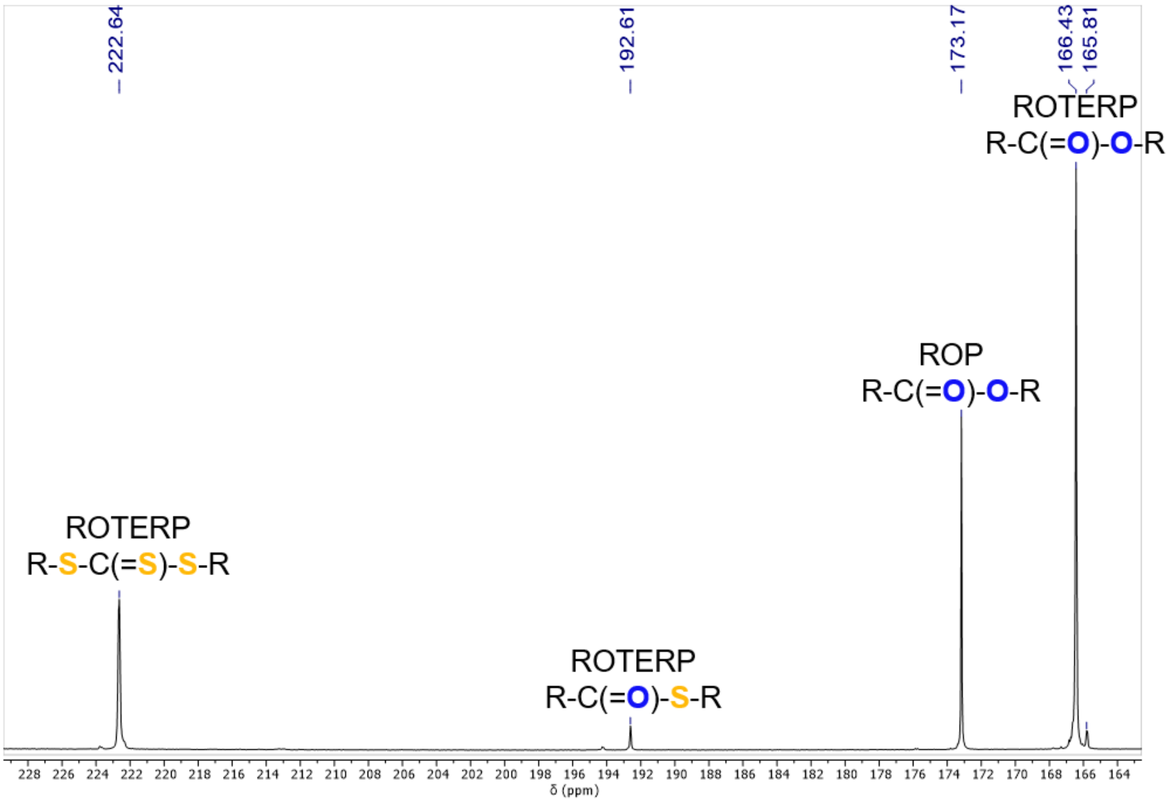

Figure S 55: Zoom into the Carbonyl region of the $^{13}C\{^1H\}$ NMR spectrum (126 MHz, $CDCl_3$, 25°C) of εDL ROP to PTA/CS_2/BO ROTERP polymer.

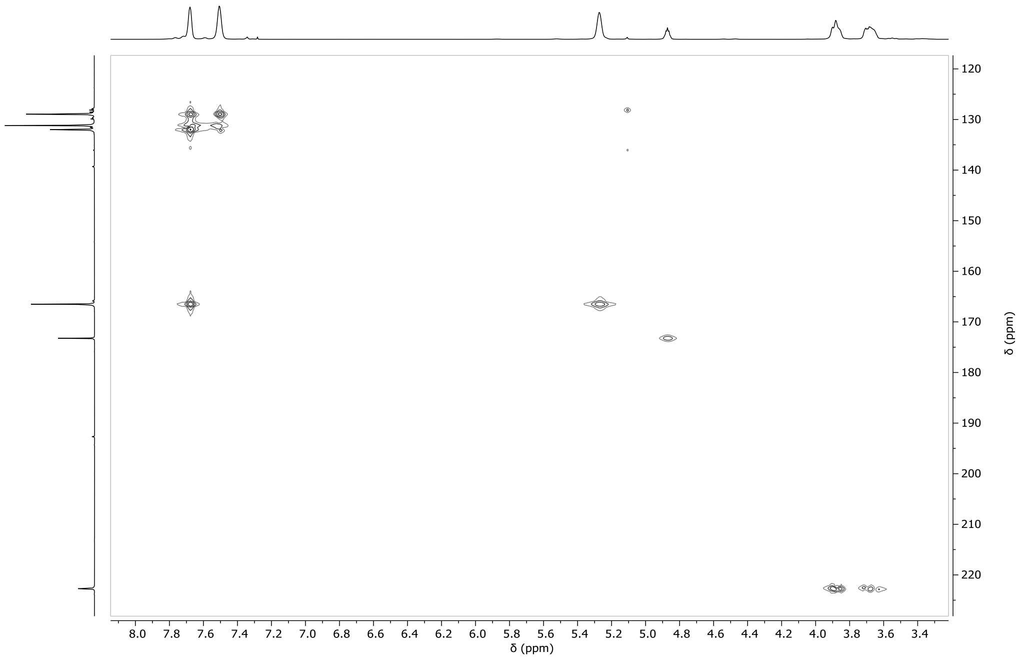

Figure S 56: ^{1}H - ^{13}C HMBC NMR spectrum (126 MHz, $CDCl_3$, 25°C) of εDL ROP to PTA/CS_2/BO ROTERP polymer.

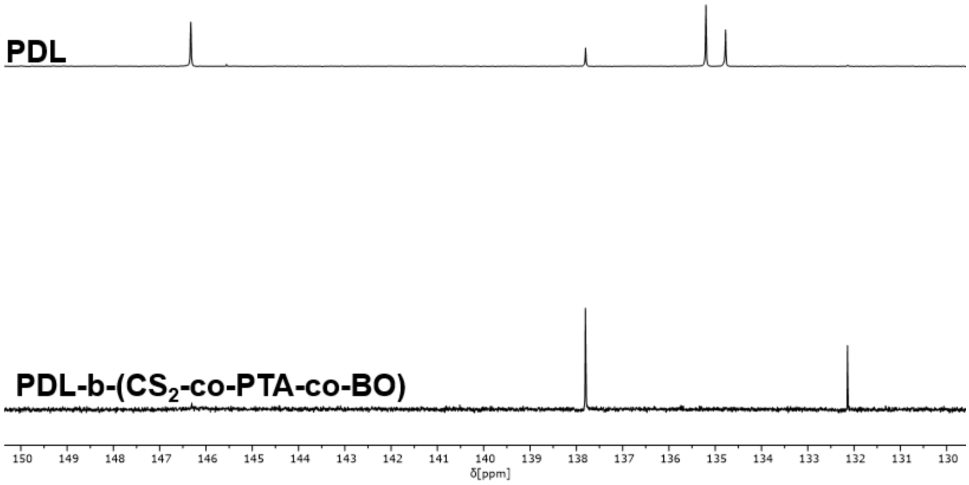

Figure S 57: $^{31}P\{^{1}H\}$ NMR (162 MHz, $CDCl_3$, 25°C) end-group analysis before and after mechanistic switch.

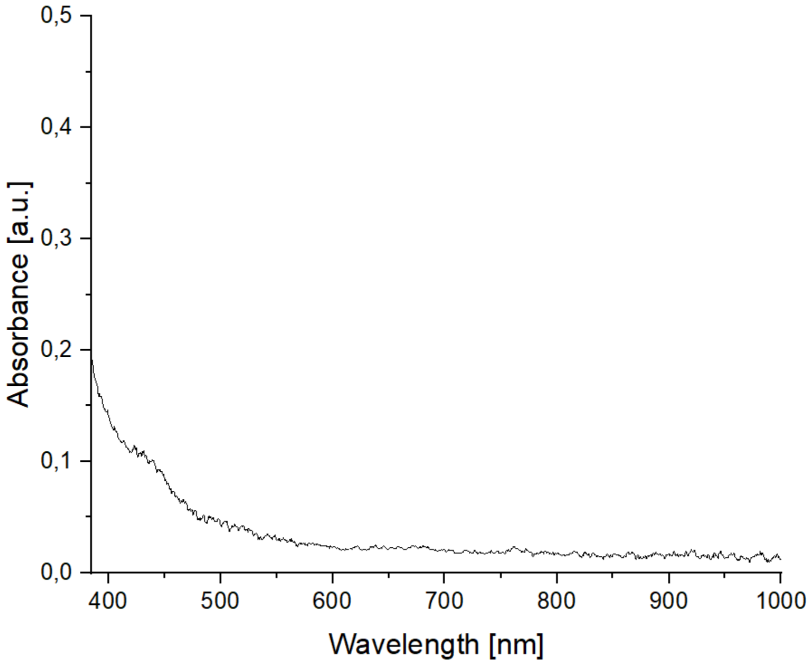

Figure S 58: UV-Vis spectrum of aliquot removed directly after addition of CS_2 and PTA (diluted with appropriate BO/CS_2 mixture by a factor of 10 prior to measurement).

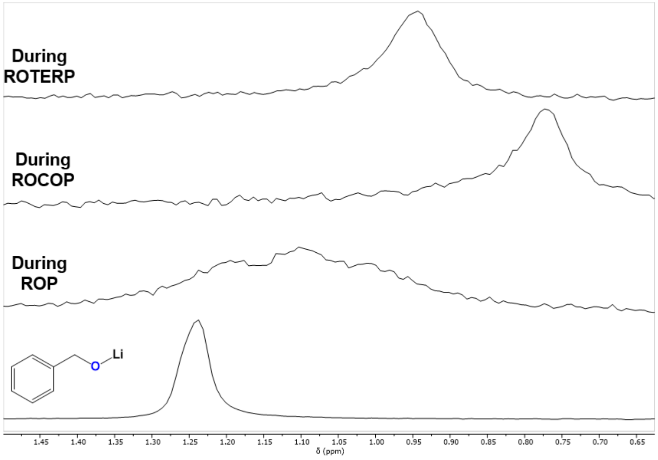

Figure S 59: Overlayed ^{7}Li NMR spectra (194 MHz, d_6-Acetone capillary, 25°C) at different stages of switchable catalysis. ROCOP refers to adding CS_2 alone without PTA.

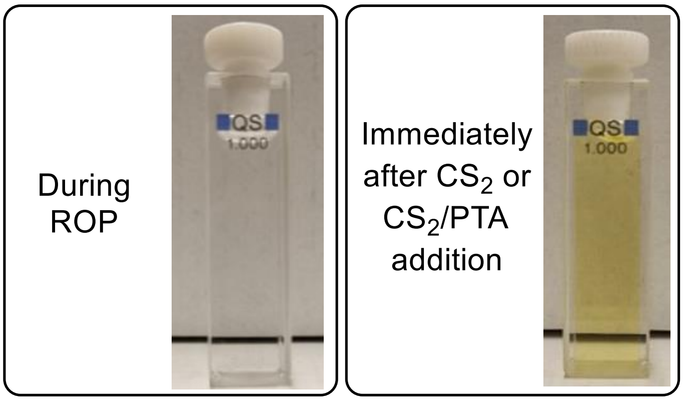

Figure S 60: Reaction colour at different stages of switchable catalysis.

Section S7: Degradation Experiments

UV degradation: The respective polymer (50 mg) was dissolved in $CDCl_3$ (0.75 mL) and irradiated for 16h by a broadband Hg UV 1000W UV lamp inside a J. Youngs NMR tube. Afterwards the sample was analysed by ^{1}H NMR spectroscopy and SEC.

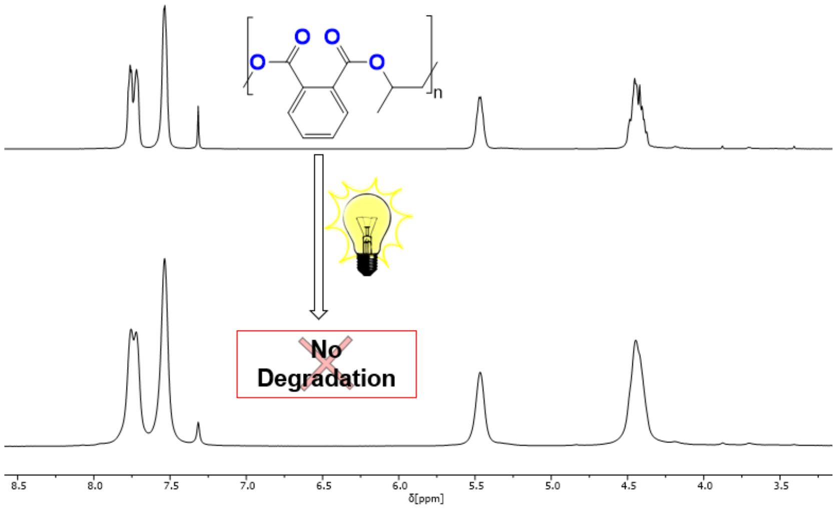

Figure S 61: ^{1}H NMR spectra (400 MHz, $CDCl_3$, 25°C) phtalic anhydride/PO copolymer before and after irradiation with UV-light.

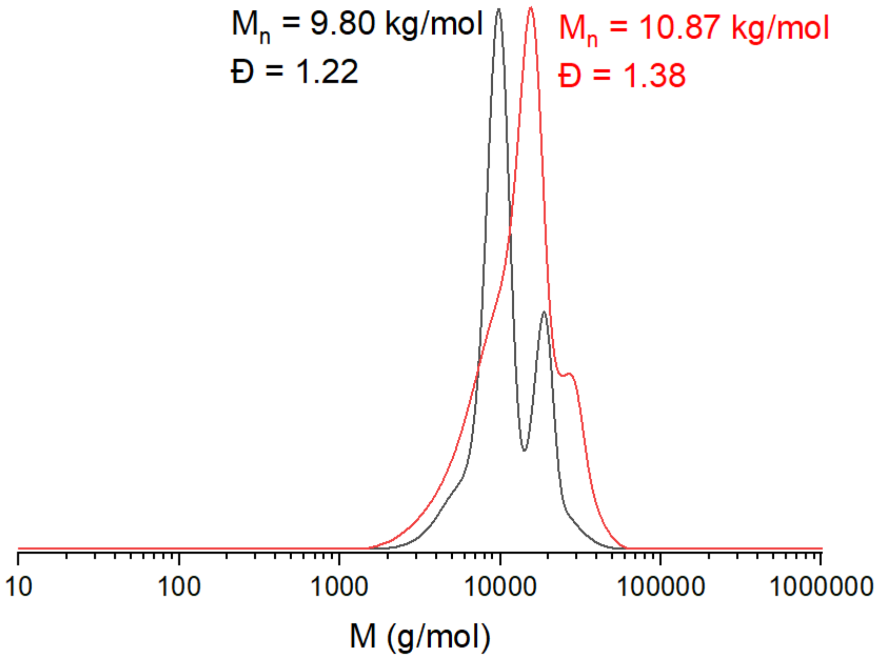

Figure S 62: Overlayed SEC traces of phtalic anhydride/PO copolymer before and after irradiation with UV-light.

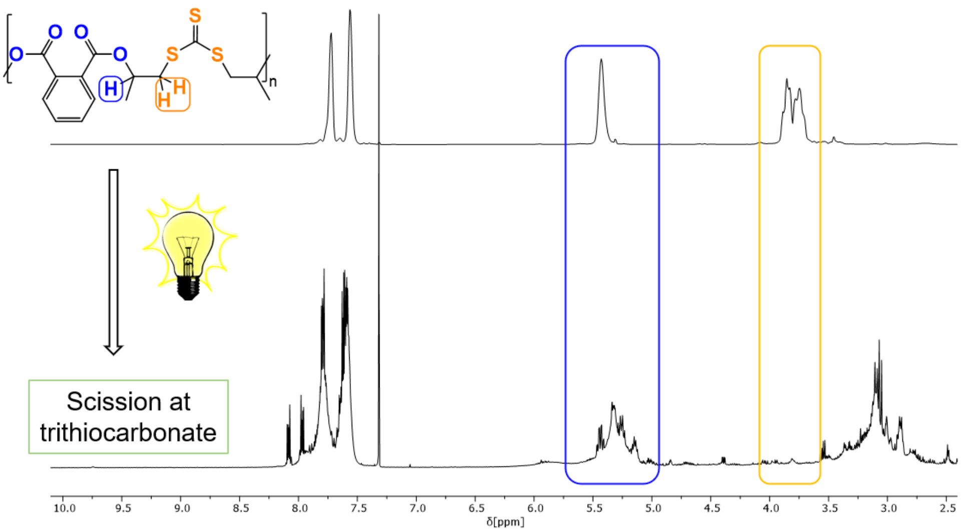

Figure S 63: ^{1}H NMR spectra (400 MHz, $CDCl_3$, 25°C) terpolymer (top) before and (bottom) after irradiation with UV-light.

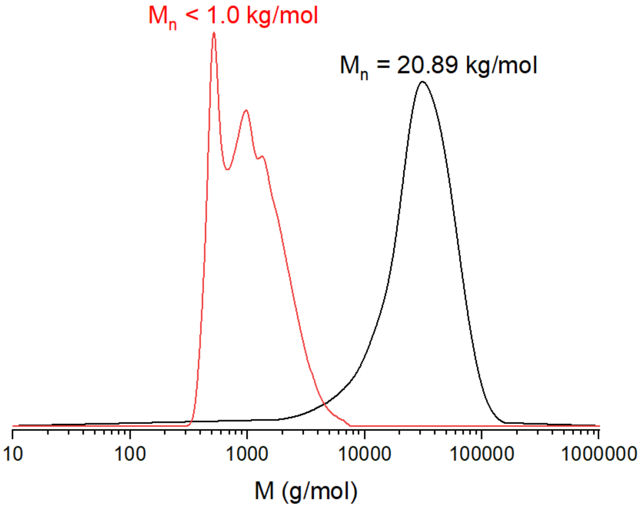

Figure S 64: Overlayed SEC traces before and after UV irradiation of terpolymer before (black) and after (red) irradiation with UV-light

Oxidative degradation: The respective polymer (50mg) was dispersed in aqueous H_2O_2 (3 mL) and stirred for 5d in a glass vial. In case of the polyester, all polymer remained dispersed and SEC as well as NMR remained unchanged. In case of the ROTERP polymer the initial yellow suspension gradually turned into a colourless solution with minor amounts of a colourless precipitate. The suspension was extracted with $CDCl_3$, dried over $MgSO_4$, filtered and analysed by NMR and SEC.

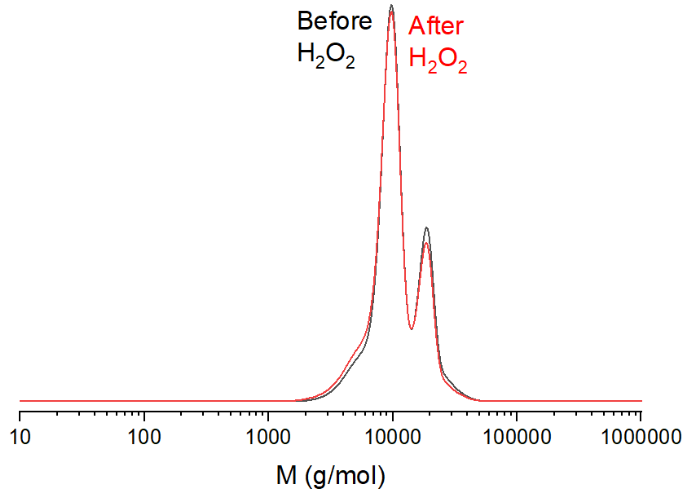

Figure S 65: Overlayed SEC traces of phtalic anhydride/PO copolymer before and after dispersion in H_2O_2. NMR spectra remains unchanged.

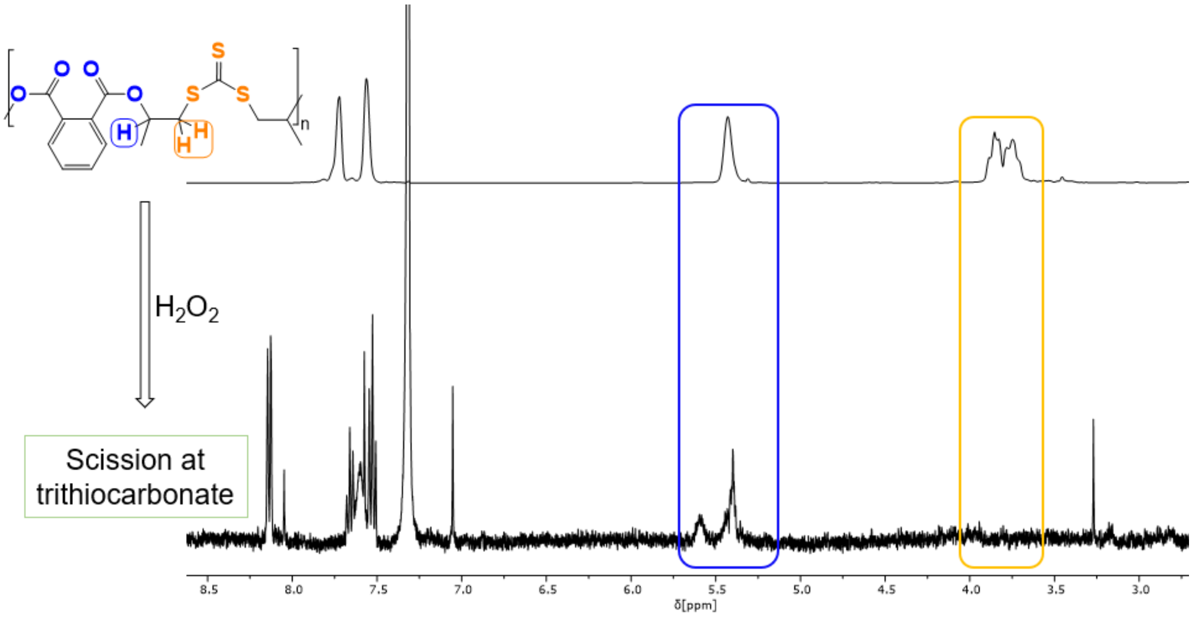

Figure S 66: ^1H NMR spectra (400 MHz, $CDCl_3$, 25°C) of terpolymer (top) before and (bottom) after oxidative degradation. No polymeric material could be detected by SEC.

Section S8: Crystallography

X-Ray data were collected on a BRUKER D8 Venture system. Data were collected at 100(2) K using graphite-monochromated Mo K_α radiation (λ_α = 0.71073 Å). The strategy for the data collection was evaluated by using the Smart software. The data were collected by the standard "*ψ-ω* scan techniques" and were scaled and reduced using Saint+software. The structures were solved by using Olex2,[3] the structure was solved with the XT[4] structure solution program using Intrinsic Phasing and refined with the XL refinement package[5,6] using Least Squares minimization. Deposition number CCDC 2151056, contains the supplementary crystallographic data for this paper. These data are provided free of charge by the joint Cambridge Crystallographic Data Centre and Fachinformationszentrum Karlsruhe Access Structures service www.ccdc.cam.ac.uk/structures.

**Compound**	**MTC**
**Empirical formula**	$C_{40}H_{58}Li_2O_{10}S_2$
**Formula weight**	776.86
**Temperature/K**	100
**Crystal system**	monoclinic
**Space group**	$P2_1/n$
**a/Å**	11.2936(6)
**b/Å**	21.8827(10)
**c/Å**	17.1083(7)
**α/°**	90
**β/°**	94.544(2)
**γ/°**	90
**Volume/Å^3**	4214.8(3)
**Z**	4
**ρ_{calc}g/cm^3**	1.224
**μ/mm^{-1}**	0.179
**F(000)**	1664.0
**Crystal size/mm^3**	0.6 × 0.16 × 0.15
**Crystal shape**	needle
**Radiation**	MoK_α (λ = 0.71073)

**2Θ range for data collection/°**	4.068 to 50.842
**Index ranges**	-13 ≤ h ≤ 13, 0 ≤ k ≤ 26, 0 ≤ l ≤ 20
**Reflections collected**	7771
**Independent reflections**	7771 [R_{int} = 0.0, R_{sigma} = 0.0235]
**Data/restraints/parameters**	7771/0/604
**Goodness-of-fit on F^2**	1.116
**Final R indexes [I>=2σ (I)]**	R_1 = 0.0472, wR_2 = 0.0929
**Final R indexes [all data]**	R_1 = 0.0561, wR_2 = 0.0967
**Largest diff. peak/hole / e $Å^{-3}$**	0.26/-0.24

**Compound**	**MTC^{Na}**	**MTC^{K}**
**Empirical formula**	$C_{12}H_{13}NaO_3S$	$C_{96}H_{104}K_8O_{24}S_8$
**Formula weight**	260.27	2211.07
**Temperature/K**	100	100
**Crystal system**	triclinic	monoclinic
**Space group**	P-1	P2/n
**a/Å**	5.9833(6)	15.1783(13)
**b/Å**	9.3642(10)	6.6961(6)
**c/Å**	11.7461(12)	30.176(3)
**α/°**	90.127(4)	90
**β/°**	101.890(4)	104.412(3)
**γ/°**	97.726(4)	90
**Volume/$Å^3$**	637.85(11)	2970.4(5)
**Z**	2	1
**ρ_{calc}g/cm^3**	1.355	1.236
**μ/mm^{-1}**	0.280	0.492
**F(000)**	272.0	1152.0
**Crystal size/mm^3**	0.24 × 0.24 × 0.11	0.5 × 0.14 × 0.1
**Crystal shape**	Colorless	colorless

**Crystal color**	needle	needle
**ion**	MoKα (λ = 0.71073)	MoKα (λ = 0.71073)
**2Θ range for data collection/°**	4.392 to 53.054	4.18 to 50.904
**Index ranges**	-7 ≤ h ≤ 7, -11 ≤ k ≤ 11, 0 ≤ l ≤ 14	-18 ≤ h ≤ 18, -8 ≤ k ≤ 8, -36 ≤ l ≤ 36
**Reflections collected**	2612	48719
**Independent reflections**	2612 [R_{int} = 0.0, R_{sigma} = 0.0314]	5700 [R_{int} = 0.1002, R_{sigma} = 0.0441]
**Data/restraints/parameters**	2612/0/157	5700/0/314
**Goodness-of-fit on F^2**	1.054	1.150
**Final R indexes [I>=2σ (I)]**	R_1 = 0.0533, wR_2 = 0.1478	R_1 = 0.0819, wR_2 = 0.2408
**Final R indexes [all data]**	R_1 = 0.0598, wR_2 = 0.1521	R_1 = 0.0829, wR_2 = 0.2420
**Largest diff. peak/hole / e Å^{-3}**	0.48/-0.46	1.85/-0.64

Section S9: Bibliography

[1] L.-Y. Wang, G.-G. Gu, T.-J. Yue, W.-M. Ren, X.-B. Lu, *Macromolecules* **2019**, *52*, 2439–2445.
[2] A. Spyros, D. S. Argyropoulos, R. H. Marchessault, *Macromolecules* **1997**, *30*, 327–329.
[3] O. V. Dolomanov, L. J. Bourhis, R. J. Gildea, J. a. K. Howard, H. Puschmann, *J Appl Cryst* **2009**, *42*, 339–341.
[4] G. M. Sheldrick, *Acta Cryst.* **2015**, *A71*, 3–8.
[5] G. M. Sheldrick, *SHELXL Version 2014/7, Program for Chrystal Structure Solution and Refinement*, Göttingen, Germany, **2014**.
[6] G. M. Sheldrick, *Acta Cryst.* **2008**, *A64*, 112–122.
